# Genome-wide mapping of replication stress response factors reveals the management of polycistronic transcription, replication initiation, and responses to replication stress in *Leishmania*

**DOI:** 10.1093/nar/gkag754

**Published:** 2026-08-10

**Authors:** Jennifer A Black, Stela Virgilio, Matheus S Bastos, Gabriel L A Silva, Jeziel D Damasceno, Kathryn Crouch, Craig Lapsley, Richard McCulloch, Luiz R O Tosi

**Affiliations:** Department of Cellular and Molecular Biology, Ribeirão Preto Medical School, University of São Paulo, Ribeirão Preto, 14040-900 SP, Brazil; University of Glasgow Centre for Parasitology, School of Infection and Immunity, Sir Graeme Davies Building, 120 University Place, Glasgow G12 8TA, United Kingdom; Department of Cellular and Molecular Biology, Ribeirão Preto Medical School, University of São Paulo, Ribeirão Preto, 14040-900 SP, Brazil; Department of Cellular and Molecular Biology, Ribeirão Preto Medical School, University of São Paulo, Ribeirão Preto, 14040-900 SP, Brazil; Department of Cellular and Molecular Biology, Ribeirão Preto Medical School, University of São Paulo, Ribeirão Preto, 14040-900 SP, Brazil; University of Glasgow Centre for Parasitology, School of Infection and Immunity, Sir Graeme Davies Building, 120 University Place, Glasgow G12 8TA, United Kingdom; University of Glasgow Centre for Parasitology, School of Infection and Immunity, Sir Graeme Davies Building, 120 University Place, Glasgow G12 8TA, United Kingdom; University of Glasgow Centre for Parasitology, School of Infection and Immunity, Sir Graeme Davies Building, 120 University Place, Glasgow G12 8TA, United Kingdom; University of Glasgow Centre for Parasitology, School of Infection and Immunity, Sir Graeme Davies Building, 120 University Place, Glasgow G12 8TA, United Kingdom; University of Glasgow Centre for Parasitology, School of Infection and Immunity, Sir Graeme Davies Building, 120 University Place, Glasgow G12 8TA, United Kingdom; Department of Cellular and Molecular Biology, Ribeirão Preto Medical School, University of São Paulo, Ribeirão Preto, 14040-900 SP, Brazil

## Abstract

Exposed single-stranded DNA (ssDNA) accumulating at stalled or collapsed replication forks (RFs) can trigger the replication stress response (RSR) to prevent genome instability. *Leishmania* are parasitic eukaryotes whose plastic genomes facilitate rapid adaptations under stress, with intrinsic replication stress (RS) implicated as a source. Little is known about the *Leishmania* RSR. Here, we reveal the global dynamics of the RSR in *Leishmania major* by performing ChIP-seq, focusing on two key RSR proteins, γH2A and RPA1, in the absence and presence of RS. We show that common RS ‘hotspots’ coincide with DNA replication initiation and transcription termination in *Leishmania*. When replication is stalled, RSR factors accumulate at early S-phase origins, with a signal pattern reminiscent of bidirectional RF progression. Under chronic RS conditions, increased accumulation of RSR factors emerges at wider sites of transcription initiation, suggesting *Leishmania* may possess compensatory strategies to limit the effects of RS and ensure DNA replication completion under stress. In contrast, chronic RS enhances RSR factor accumulation at transcription termination sites, highlighting these regions as RS ‘hotspots’ in *Leishmania*. Lastly, variations in replication protein A (RPA) dynamics in Ataxia Telangiectasia and Rad3 Related (ATR)-deficient cells uncover crucial roles of this kinase in managing polycistronic transcription and DNA replication, particularly under RS, in *Leishmania*.

## Graphical Abstract

**Graphical Abstract:**
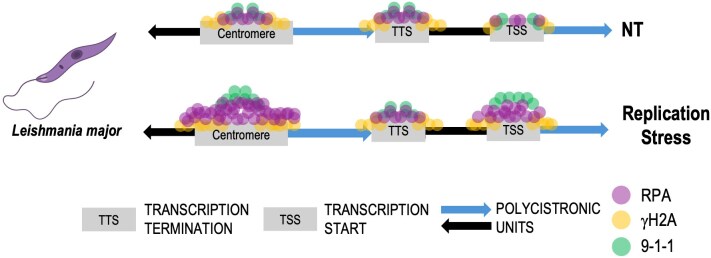

## Introduction

In most eukaryotes, DNA synthesis begins at the onset of S-phase from multiple DNA replication origins per chromosome licensed during late mitosis and G1 phase [1]. At replication forks (RFs), the replicative helicase moves along the leading DNA strand to unwind the double helix, exposing single-stranded DNA (ssDNA). The DNA is then copied through the actions of DNA polymerases. If these activities are uncoupled, exposed ssDNA becomes susceptible to damage and secondary structure formation that can undermine genome stability [2]. To preserve ssDNA integrity, single-stranded binding proteins bind to and protect ssDNA, a role primarily fulfilled in eukaryotes by the heterotrimeric complex replication protein A (RPA) [3, 4]. RPA-coated ssDNA then recruits the kinase Ataxia Telangiectasia and Rad3 Related (ATR) and replication stress response (RSR) factors including, but not limited to, the heterotrimeric clamp 9-1-1 (RAD9-RAD1-HUS1) and ATR’s obligatory interacting partner (ATR-interacting partner; ATRIP) [5, 6]. Activated ATR phosphorylates a myriad of downstream factors to halt cell cycle, stabilize RFs, and slow origin firing, ultimately limiting the formation of harmful double-strand breaks (DSBs) [6–12]: inhibiting ATR or following prolonged periods of replication stress (RS) induces ssDNA–RPA accumulation, increased origin firing, and a rise in stalled and collapsed RFs [5, 7, 9, 10]. Given the importance of RPA during DNA replication and in the RSR, this complex has been instrumental in mapping the genome-wide distribution of collapsed RFs [7, 13], and wider regions of DNA damage [14]. Mapping the dynamics of yH2AX and 9-1-1 during RS has been used previously to locate damaged RFs and DNA breaks [14, 15].

*Leishmania* are parasitic protozoans that cause leishmaniasis, a neglected tropical disease endemic in ∼90 countries across the tropics and subtropics of the world [16–18]. Unusually, *Leishmania* possess remarkably plastic genomes, a phenomenon broadly defined as the capacity of a genome to tolerate rapid rearrangements in composition [19–21]. Genome plasticity can manifest as chromosome and gene copy number variants (CNVs), single nucleotide variants (SNVs), and extrachromosomal DNA molecules. These alterations can be associated with negative consequences, such as the development of diseases like cancer in multicellular eukaryotes [22]; however, for *Leishmania* and related kinetoplastids, the almost complete reliance on polycistronic genome transcription may explain why the *Leishmania* genome endures extensive chromosome and CNVs [20, 23–26] that target gene dose. Additionally, recombination between so-called ‘repeat elements’ promotes the stochastic emergence of selectable extrachromosomal DNA elements as either linear or circular molecules of DNA [27–30], commonly reported alongside the emergence of phenotypic alterations and drug resistance [24, 27, 29, 31–36]. This plastic nature of the *Leishmania* genome strongly supports the presence of a less stringent RSR, particularly given subtelomeric duplication may require components of the 9-1-1 complex in *Leishmania* [37]. However, alterations to the RSR in this parasite may have consequences for polycistronic transcription, potentially posing a unique challenge for the management of replication-transcription conflicts [38].

Marker Frequency Analysis followed by next generation sequencing (MFA-seq) revealed a single early-S replication initiation site per chromosome, located within a strand switch region (SSR). These specific SSRs were enriched for (i) acetylated histone H3 (AcH3), associated with transcription initiation activities [39], (ii) Base J (β-D-glucopyranosyloxymethyluracil), a heavily modified thymidine base required for transcription termination [40, 41], and the kinetochore factor KKT1 [37, 42], suggesting these sites additionally support centromere formation [43]. Moreover, MFA-seq supports the co-orientation of the leading strand with transcription, potentially enabling *Leishmania* to avoid machinery collisions within the chromosome cores [43]. Single Molecular Analysis of Replicated DNA revealed multiple origins per chromosome, but their locations relative to transcriptional activity are unknown [44], whereas Short Nascent Strand (SNS-seq [45]) suggests that *Leishmania* chromosomes possess hundreds of origins, though these sites overlap with features related to messenger RNA (mRNA) maturation, leaving their role in replication initiation unclear. A newly merged picture of replication dynamics in *Leishmania* supports *Leishmania* DNA replication initiation to be mutagenic, with the accumulation of SNVs in the vicinity of initiation sites [42]. Moreover, this newer model supports that DNA replication initiation begins at S-phase onset from a singular site per chromosome and completes from multiple, stochastic initiation sites scattered across the chromosomes.

Despite advances in our understanding, how *Leishmania* manages interactions between DNA replication and transcription machineries is unclear. Genomic regions at the ends of multigenic units, SSRs, are used to regulate both replication and transcription activities [37, 45, 46]. In *Leishmania*, transcription initiation sites are marked by, though not necessarily limited to [47], AcH3: [48–50] and in *L. donovani* H4 (AcH4K10 [51]), and transcription termination sites (TTSs) by Base J [48–50]. However, little is known about the RSR in *Leishmania*. The *Leishmania* genome encodes a complete 9-1-1 complex, RPA complex, and ATR kinase, suggesting core aspects of the RSR are retained, with functional studies of these factors revealing roles in cell cycle progression, RS, and the response to DNA damage [52, 53]. However, *Leishmania* appears to lack a CHK1 homolog (downstream target of ATR) [54, 55] and additionally harbours divergent 9-1-1 [53, 56] and RPA functions, raising questions as to the specific dynamics of their RSR.

RPA1 proteins in higher eukaryotes typically possess four N-terminal oligonucleotide/oligosaccharide-binding fold (OB-fold domains) used for ssDNA binding and protein–protein interactions; one termed 70N and the other three called DNA-binding domains (DBD-A, DBD-B, DBD-C) [57–59]. In contrast, *Leishmania* RPA1 lacks the 70N domain [60] that facilitates key protein–protein interactions including with cell cycle regulatory proteins like p53, ATRIP, and RAD9 of the 9-1-1 complex [61]. Though homologs of ATRIP and p53 are yet to be identified in the *Leishmania* genome, the absence of the 70N domain *Leishmania* RPA1 raises questions as to how RPA and 9-1-1 interactions operate. Instead, *Leishmania* RPA1 is composed of three OB-fold domains, of which only the first domain (OBF1) is required for ssDNA interactions. Unusually, RPA1 in *L. amazonesis* binds G-rich telomeric ssDNA, suggesting RPA1 in trypanosomatids may act during telomere metabolism [61] in addition to canonical functions in the DNA damage response and ssDNA binding [60, 62–64]. Investigations into RPA2 in *T. brucei* reveal functions associated with both DSBs [65] and the RSR [66], whereas in *T. cruzi*, in addition to canonical functions in DNA replication and the response to damage, RPA function has been linked to parasite metacyclogenesis [67], suggesting the RPA complex has broad functions in these parasites. However, given RPA1’s capacity to interact and bind ssDNA in *Leishmania*, and the demonstration of a direct interaction between RPA1 and RPA2 in *T. cruzi* [67], we reasoned that RPA1, as a proxy for the RPA complex, would enable the global profiling of the interactions between a protein that is located directly at RFs and is functionally linked to the RSR in *Leishmania*. Herein, we performed ChIP-seq on *Leishmania major* promastigote cells expressing endogenously tagged RPA1, in the absence and presence of acute and chronic RS conditions induced by hydroxyurea (HU). To assess genotoxic stress and putative sites of DNA damage, we mapped the locations of yH2A, a marker of DNA damage [68, 69]. Additionally, to investigate the process under sustained RS, we analysed RPA1 dynamics in an ATR-deficient mutant.

Altogether, our work highlights key events of replication–transcription conflict or DNA replication initiation found at the boundaries of polycistronic transcription units (PTUs), reflecting the unique organization of gene expression in this and related kinetoplastids. The relative lack of such interactions within the PTUs suggests undetected mechanisms to minimize conflicts between actively moving DNA replication and transcription machineries. Furthermore, variations in peak sizes and signal distribution in ATR-deficient cells reveals key roles for this kinase in the management of multigenic transcription and genome duplication, exemplified by induction of RS, in *L. major* promastigotes.

## Materials and methods

### Lead contact and materials availability

All enquiries regarding additional information/reagents and resources should be directed to and will be fulfilled by the Lead Contact, Prof. Luiz R.O. Tosi. No novel reagents were generated in this work.

### gRNA and donor DNA amplification by polymerase chain reaction

For single guide RNA (sgRNA) amplification: 0.2 mM dNTPs, 2 µM of sgRNA scaffold, and an RPA1-specific forward primer and were mixed in 1× HiFi reaction buffer with MgCl_2_ and 1 unit Phusion High-Fidelity DNA Polymerase (New England BioLabs), 25 µl total volume. Cycling conditions were as follows: 30 s at 98°C followed by 35 cycles of 10 s at 98°C, 30 s at 60°C, and 15 s at 72°C. For donor amplification: 30 ng of the required pPLOT plasmid [70] was used as a template vector and combined with 0.1 mM dNTPs, 1 µM each of RPA1 donor forward and reverse primers, 1 unit Phusion High-Fidelity DNA Polymerase (New England BioLabs), and 1× HF reaction buffer with MgCl_2_. Cycling conditions were as follows: 5 min at 94°C followed by 40 cycles of 30 s at 94°C, 30 s at 60°C, 2 min 30 s at 72°C, followed by a final extension step for 7 min at 72°C. Oligonucleotides sequences are provided in Supplementary Table S1.

### Cell lines and culture

*Leishmania major* LT252 (MHOM/IR/1983/IR) promastigotes were cultured in M199 medium supplemented with 10% heat-inactivated fetal bovine serum (FBS; Sigma–Aldrich) at 27°C as described [53, 71]. Approximately 2 × 10^7^ exponentially growing Cas9-T7-expressing LT252 cells were transformed with ∼4 µg of guide and donor DNA. Cells and DNA were resuspended in 250 μl of Roditi BSF buffer (90 mM sodium phosphate, 5 mM potassium chloride, 0.15 mM calcium chloride, and 50 mM HEPES, pH 7.3) [72] and electroporated using the program X-001 (Amaxa Nucleofector IIb; Lonza). Parasites were recovered overnight in M199 with 10% additional FBS (20% FBS in total). Transformed cells were isolated by limiting dilution in 96-well plates and selected for by the addition of blasticidin (10 μg·ml^−1^). Endogenously tagged RAD9 (RAD9^12myc^)-expressing LT252 parasites were generated prior to this study [73]. Parasite proliferation was quantified by assessing cell density every 24 h using a Neubauer chamber. Parasites were seeded at a density of 1 × 10^5^ cells. ml^−1^, and growth was monitored for 5 days (120 h). Survival curves were performed as follows: cells were seeded at a density of 1 × 10^5^ cells. ml^−1^, then HU (Sigma) was added at the appropriate concentrations. Living cells were then quantified by counting after 96 h.

### Genomic DNA extraction and general polymerase chain reaction protocol

For genomic DNA extractions for polymerase chain reaction (PCR) applications and whole-genome sequencing, the Qiagen Blood and Tissue Extraction Kit ® (Qiagen) was used as per the manufacturer’s instructions. PCRs were performed using either Platinum Taq® (Thermo Fisher) or Phusion® (NEB or Thermo Fisher) as per the manufacturer’s instructions. GC buffer was used for most Phusion® reactions.

### Flow cytometry

DNA content was assessed by propidium iodide (PI) staining and analysed as described [74]. For experiments involving cell synchronization with HU, exponentially growing cells were incubated with 5 mM HU for 8 h. Cells were then washed 1× and recovered in HU-free medium to allow cell cycle progression. To assess the cell cycle under RS conditions, exponentially growing cells were treated with 5 mM HU for 2 and 8 h, and 0.5 mM HU for 8 and 20 h. Cells were harvested by centrifugation, washed once with 1× phosphate-buffered saline (PBS), and fixed in 30% 1× PBS/70% methanol or ethanol overnight at 4°C or −20°C. Fixed cells were washed with 1× PBS and stained in 1× PBS containing PI (10 μg·ml^−1^) and RNase A (10 μg·ml^−1^) at 37°C for 30 min in the dark. Flow cytometry data were collected using a BD FACSCanto or BD FACSFortessa cytometer (BD Biosciences). Data were analysed using FlowJo. HU was prepared fresh in M199, typically to a maximum concentration of 200 mM.

### Immunofluorescence analysis

Cells were harvested by centrifugation (1500 × *g*), washed with 1× PBS, fixed with 4% paraformaldehyde prepared in 1× PBS for 15 min, and then washed with 1× PBS. The cells were settled on 0.01% poly-L-lysine (Sigma)-treated slides, then permeabilized in 1× PBS, 0.2% Triton X-100 (PBS-T) for 5 min. Cells were blocked with 1% bovine serum albumin (BSA) in PBS-T for 1 h, then incubated with anti-myc antibody (Sigma or Merck Millipore) at 1:100 or 1:500 dilution in PBS-T, 1% BSA for 1 h. For yH2A staining, Trypanosome anti-yH2A (provided by McCulloch Lab) was used at a concentration of 1:1000. After, cells were washed in 1× PBS, then incubated for 1 h anti-mouse AlexaFluor488^TM^ (Thermo Fisher) at a 1:1000 dilution and/or anti-rabbit Alex488 Plus (Thermo Fisher) at a dilution of 1:1000 for 1 h. Antibody incubations were performed in a wet chamber in the dark at room temperature (RT). DNA was stained with Hoechst 33 342, and cells were mounted with ProLong^®^ Gold Antifade Reagent (Thermo Fisher), or DNA was stained with DAPI using Fluromount-G containing DAPI (Thermo Fisher). Slides were sealed with nail varnish, and the slides stored in the dark at 4°C until required. Images were captured on a Zeiss Confocal Multiphoton microscope or using a Leica Fluorescent microscope. Images were prepared and analysed in Fiji (ImageJ) [75].

### EdU incorporation by IFA

For DNA replication quantification by immunofluorescence analysis (IFA), promastigote cells were seeded at a density of 8 × 10^5^ cells. ml^−1^ and cultured in the presence or absence of HU (5 or 0.5 mM). Thirty minutes prior to the desired time point, 150 µM ethynyl deoxyuridine (EdU) was added, and the cells incubated in 12-well plates in the dark at 27°C. Afterward, cells were harvested by centrifugation 2300 × *g* for 3 min, and then washed 1× with 1× PBS. Cells were resuspended in 200 µl 1× PBS, and then 200 µl of 2% paraformaldehyde (prepared in 1× PBS) was added. Cells were gently homogenized, and then incubated at RT for 20 min. Afterward, cells were harvested by centrifugation for 10 min at 1000 × *g*. The supernatant was then discarded, the cells were washed twice in 1× PBS, and then the cells were resuspended in 200 µl 1× PBS and stored at 4°C until required. Cells were permeabilized in 200 µl 1% Triton X-100 in 1× PBS for 10 min (room temperature), centrifuged for 10 min at 1000 × *g*, and then washed twice in 500 µl 3% BSA in 1× PBS. Cells were resuspended in 100 µl 1% BSA in 1× PBS, and then settled for 30 min at RT on poly-L-lysine (0.01%; Sigma)-treated slides. Wells were then washed twice with 3% BSA in 1× PBS, and the EdU Click-IT reaction cocktail was prepared as per the manufacturer’s instructions. Cells were incubated for 1 h 30 min with the Click-IT reaction, and then the wells washed 6× for 5 min each with 3% BSA in 1× PBS. For yH2A staining, trypanosome anti-yH2A was diluted in 1% BSA 1× PBS at 1:1000, and the wells were incubated for 1 h. Afterward, wells were washed with 1% BSA in 1× PBS twice for 5 min, and then anti-rabbit Alex488 Plus (Thermo Fisher) added for 2 h. Afterward, wells were washed for 5 min 3× with 1% BSA in 1× PBS. Five microliters of DAPI Fluromount-G were added to each well to stain nuclear and kinetoplast DNA, and then the slide was sealed with nail varnish. Slides were stored in the dark at 4°C until required. Images were captured using a Leica DMI6000B Fluorescent microscope. Images were prepared and analysed in Fiji (ImageJ) [75].

### ssDNA quantification by IFA

For ssDNA quantification by IFA, 5 × 10^5^ cells. ml^−1^ were grown for 16 h at 27°C in 250 µM IdU (Sigma–Aldrich; protected from light). Cells were then harvested by centrifugation (1400 × *g*, 5 min) at the time points described in the absence and presence of HU treatment. After, cells were fixed in 4% paraformaldehyde (500 µl prepared in 1× PBS) for 15 min (agitating, RT), washed in 1× PBS as before then stored in 1× PBS at 4°C in the dark until required. Cells were settled for 30 min at RT on poly-L-lysine (Sigma)-treated slides, then permeabilized in 1× PBS + Triton X-100 (5%) for 20 min at RT. Cells were then washed 5× with 1× PBS then blocked in 1× PBS + BSA (1%) for 1 h, RT. After blocking, cells were incubated for 2 h RT with anti-BrdU (mouse; BD Biosciences) antiserum (1:100) diluted in 1× PBS. After, cells were washed 8× in 1× PBS. Cells were then incubated for 30 min with anti-mouse AlexaFluor488 (1:500) at RT. After, cells were washed 8× in 1× PBS then mounted in a DAPI-containing mounting medium (Southern Biotech). Slides were sealed with nail varnish then stored at 4°C in the dark until required. To confirm uniform labelling with IdU, controls were denatured using 2N HCL at RT for 30 min, washed 5× in 1× PBS then neutralized in phosphate buffer 0.2M (0.2M Na_2_HPO_4_, 0.2M KH_2_PO_4_, pH 7.4) for 10 min. Cells were then washed 5× in 1× PBS then stained as above. Images were captured on a Zeiss Multiphoton Confocal microscope, and the fluorescence intensity was quantified using Fiji (ImageJ) [75].

### Image processing

For images captured on a Leica DMI6000B Fluorescent microscope, a 100× magnification lens (oil) was used, and images acquired using the LAS X software platform (Leica). For images captured on a Zeiss Multiphoton Confocal microscope, a 63× magnification lens (oil) was used, and images were acquired using Zen software (Zeiss). For images captured on a Leica Super Resolution Stellaris 8, a 63× magnification lens (oil) was used, and images were acquired using the LAS X software platform (Leica). All fluorescent channels for each image captured within each experiment were taken using the same fluorescence intensity and gain. Z-stacks were not captured. Fiji [75] was used to remove background signal, to calculate intensity, and to count cells. Brightness and contrast were set relative to the appropriate unstained control. Fluorescent channels were assigned false colours, and signal was enhanced to improve visualization. Scale bars are as indicated.

### Cell fractionation

Soluble and chromatin-bound proteins were fractionated as described before with the following modifications [53]. Briefly, ∼1 × 10^8^ cells were washed with 1× PBS and incubated with buffer A (10 mM Tris–HCl, pH 9.0, 100 mM NaCl, 0.1% Triton X‐100, 300 mM sucrose, 10 mM MgCl_2_, 10 mM Na_3_VO_4_, 10mM β‐glycerophosphate disodium; 3× SIGMAFAST protease inhibitor cocktail [Sigma]) for 10 min on ice. Samples were then centrifuged (5 min; 3900 × *g*; 4°C), and the supernatant was collected (the soluble fraction; Soluble I). The precipitated insoluble material was treated with buffer A once more, centrifuged as before, then the second soluble fraction (Soluble II) was collected. The remaining pellets were treated with DNase I (Thermo Fisher) (100 units for 5 × 10^7^ cells) for 1 h at 37°C. The sample was then centrifuged (5 min; 5000 × *g*; 4°C), and the supernatant (containing chromatin) was collected. Proteins were separated as detailed below.

### Immunoblotting

Sodium dodecyl sulfate–polyacrylamide gel electrophoresis (SDS–PAGE) was used to resolve proteins. Gels were prepared in-house, and typically 10% gels were prepared and ran at 100 V for 3 h. Proteins were then transferred to polyvinylidene difluoride (PVDF) membrane or nitrocellulose membrane. Before probing for specific proteins, membranes were blocked with 10% (w/v) non-fat dry milk in PBS. ECL Prime Western Blotting Detection Reagent (GE Life Sciences) was used for band detection and visualized with Hyperfilm ECL (GE Life Sciences) or using a GE ImageQuant LAS-4000 Multi-Mode Imager.

### Southern blotting

For Southern blotting, DNA was extracted as described in [71]. Genomic DNA was initially digest with NotI, and the digested products were separated by gel electrophoresis using the following conditions (0.6% agarose in 1× TAE buffer, 20 V for 16 h). DNA was transferred to a Hybond-N+ membrane (GE Life Science). Probes were generating via a PCR: probe for ATR gene (419 bp, FW-GACGAGCTGGACCGTTACAT, RV-CTCGCCTCGTAGATGCTCTG) and one for the mutated allele, in this case regions from the Puromycin gene (426 bp, FW-GTCACCGAGCTGCAAGAACT, RV-GTCCTTCGGGCACTCGAC). The hybridization was performed at 65°C. For probe labelling, the AlkPhos Direct Labelling and Detection system was as per the manufacturer’s instructions, and signal was visualized using CDP-Star developing reagent (GE Life Sciences).

### Chromatin immunoprecipitation (myc-tagged proteins)

ChIP for RPA1 and RAD9 was performed as detailed [50] with the following modifications. Exponentially growing promastigotes were harvested in the presence or absence of HU treatment. Cells were fixed in paraformaldehyde (1%; Sigma, prepared in 1× PBS) for 40 min (agitating), quenched in 125 mM Glycine (Sigma) for 5 min (agitating) at room temperature, and then samples were centrifuged at 1400 × *g* for 5 min. Next, the supernatant was discarded, the pellets were resuspended in 1× PBS/125 mM Glycine solution, and then left at room temperature (with agitation) for 5 min. Samples were centrifuged and washed as before, and then the pellets lysed in Lysis Buffer 1 (HEPES-NaOH 50 mM, pH 7.5, NaCl 140 mM, ethylenediaminetetraacetic acid (EDTA) 1 mM, glycerol 10%, NP-40 0.5%, Triton X-100 0.25%, 1× protease inhibitors [SIGMAFAST^TM^ Protease Inhibitor Cocktail Tablets, Sigma]) for 10 min (agitating) at 4°C. The samples were centrifuged again (at 4°C) and then resuspended in Lysis Buffer 2 (Tris–HCl 10 mM, pH 8.0, NaCl 200 mM, EDTA 1 mM, EGTA [ethylene glycol-bis(β-aminoethyl ether)-*N,N,N*′,*N*′-tetraacetic acid]0.5 mM, 1× protease inhibitors) for 10 min (agitating) at room temperature. Next, samples were centrifuged as before, then Lysis Buffer 3 (Tris–HCl 10 mM, pH 8.0, NaCl 100 mM, EDTA 1 mM, EGTA 0.5 mM, Na-Deoxycholate 0.1%, N-lauroylsarcosine 0.3%, 1× protease inhibitors) was added, and the samples were kept on ice prior to sonication. Sonication was performed using a QSonica Sonicator Model Q125 under the following conditions (30 cycles, 45 s ON/59 s OFF pulsing, amplitude 60%). Triton X-100 (1.1%) was then added, and the samples centrifuged at 10 000 × *g* for 2 min (4°C). For immunoprecipitation of myc-bound chromatin, Dynabeads^TM^ (Sheep anti-mouse IgG, Thermo Fisher) were washed with blocking buffer (1× PBS, BSA 0.5%, EDTA 2 mM), and then 5 µl of anti-myc antiserum (in blocking buffer) and the sonicated samples were added to the beads and left rotating for 5 h (4°C). Afterward, the beads were washed in TSE 150/500 buffer (Triton X-100 1%, SDS 0.1%, EDTA 2 mM, Tris–HCl 20 mM (pH 8.0), NaCl 150 mM/500 mM, 1× protease inhibitors), then LiCl wash buffer (LiCl 0.25 M, NP-40 1%, Na-Deoxycholate 1% EDTA 1 mM, Tris–HCl 10 mM, pH 8.0, 1× protease inhibitors), and finally in TE buffer (Tris–HCl, pH 8.0, EDTA, 1× protease inhibitors). After TE buffer was removed, the beads were resuspended in 200 µl Elution Buffer (Tris–HCl 50 mM, pH 8.0, EDTA 10 mM, SDS 1%) and incubated at 65°C for 30 min (with agitation every 5 min). Next, the elute was separated from the beads and incubated at 65°C overnight to reverse crosslinks. Afterward, 4 µl of RNAse A (20 mg/ml) was added for 2 h at 37°C, and then 4 µl of Proteinase K (20 mg/ml) was added for 2 h at 55°C. DNA was purified using the Illustra^TM^ GFX^TM^ PCR DNA and Gel Band Purification Kit (GE Healthcare) as per the manufacturer’s instructions. DNA quantification was performed using a Qubit^®^ 2.0 Fluorometer (Invitrogen).

### Chromatin immunoprecipitation (yH2A)

Suboptimal immunoprecipitation of yH2A occurred using the above protocol; therefore, a modified protocol based on Wedel and Siegel, 2017 [76] was used. Briefly, cultures were prepared and treated with HU as described earlier. Cells were harvested by centrifugation at 1000 × *g* for 10 min, the resulting pellet resuspended in M199 medium (lacking serum), and then crosslinked in using formaldehyde (50 mM HEPES-NaOH, pH 7,5, 100 mM NaCl, 1 mM EDTA, 0,5 mM EGTA, 11% (v/v) paraformaldehyde prepared in 1× PBS) for 20 min (with agitation). The reaction was quenched by the addition of 125 mM Glycine (Sigma) for 5 min at room temperature (while agitated). Afterward, samples were centrifuged at 2000 × *g* for 20 min (4°C), the supernatant was removed, and the pellets washed by centrifugation in 1× PBS. Cells were then resuspended in permeabilization buffer containing protease inhibitors (100 mM KCl, 10 mM Tris, pH 8.0, 25 mM EDTA, pH 8.0, 1× protease inhibitors [SIGMAFAST^TM^ Protease Inhibitor Cocktail Tablets, Sigma]). Fifty microlitres of Digitonin (4 mM) were added, and the samples incubated for 15 min (with agitation) at room temperature. Samples were subjected to centrifugation at 1400 × *g* for 5 min (4°C), the supernatant was discarded, the pellet was resuspended in ice-cold NP-S buffer (0.5 mM spermidine, 0.075% (v/v) IGEPAL, 50 mM NaCl, 10 mM Tris–HCl, pH 7.5, 5 mM MgCl_2_, 1 mM CaCl_2_, 1× protease inhibitors [SIGMAFAST^TM^ Protease Inhibitor Cocktail Tablets, Sigma]), and the samples were vortexed. Next, samples were centrifuged at 1400 × *g* for 5 min (4°C), the previous step repeated, and then the pellets were resuspended in ice-cold NP-S containing 4U MNase (New England Biolabs) for 10 min at 25°C. After 0.5 mM EDTA was added, the samples were centrifuged at 10 000  ×* g* for 10 min (4°C), and the supernatant with the mononucleosome fraction was transferred to a new tube. The remaining pellets were resuspended in ice-cold NP-S buffer as before containing 2 µl 10% SDS, and then subjected to sonication, on ice, for 10 cycles of 30 s on/30 s off pulses (20% amplitude; QSonica Sonicators Model Q125). Next, samples were centrifuged at 10 000 ×* g* for 10 min (4°C), and the supernatant was removed and combined with the mononucleosome fraction. The immunoprecipitation was performed as detailed for RPA1 and RAD9. Dynabeads^TM^ M-280 Sheep anti-Rabbit IgG (Thermo Fisher) were used to capture 5 µl of anti-yH2A antiserum raised against the *T. brucei* peptide [68, 69] for each sample.

### Library preparation

ChIP samples were prepared for next-generation sequencing using the TruSeq ChIP Library Preparation Kit (Illumina) at the University of Glasgow. Agencourt AMPure XP beads (Beckman Coulter) were used to size-select for 300-bp fragments (including adaptor sequence). All samples were sequenced at Glasgow Polyomics (University of Glasgow) in a NextSeq 500 Illumina machine. Paired-end read or single-end reads were generated. For the second replicate of the yH2A ChIP-seq, single-ended libraries were prepared by BGI and sequenced using DNB-seq technology, with ∼20 million single-end reads per sample (Phred + 33). ChIP-seq of RPA1, yH2A, and RPA1 in ATR-deficient cells was performed as biological duplicates. ChIP-seq experiments of RAD9 and HUS1 were performed once and are provided as supplementary data (Supplementary Data 1). Whole-genome sequencing samples were prepared for next-generation sequencing using the QIAseq FX DNA library, compatible with Illumina sequencing technology, at the University of Glasgow. Samples were sequenced as paired-end reads at Glasgow Polyomics (University of Glasgow) in a NextSeq 500 Illumina machine. Paired-end reads of 100 nt for replicate 1 (DL samples) were generated. For replicate 2 (GL samples), paired-end reads of 150 nt were generated.

### ChIP-seq data analysis

Sequence read quality was examined first by FastQC [59], and coverage depth was assessed by Samtools Depth [77]. Adapter sequences were trimmed off using TrimGalore (default setting), and the trimmed reads were mapped to the *Leishmania* LmjF.b41 reference genome (TriTrypDB.org) using Bowtie2 [78] with very-sensitive parameters. Reads were filtered to exclude reads below MapQ of 30, and duplicate reads were marked using MarkDuplicates (Picard). ‘Shade-regions’ were removed from the downstream analysis (regions of genes with high copy number as described [45]), and chromosome 31 (a super numeric chromosome) was excluded during data normalization. ChIP tracks were visualized in IGV v2.14 [79], and, due to the broad nature of the signal, replicates were merged for RPA in ATR-competent and ATR-deficient cells. Input and IP samples were normalized using DeepTools [80] bamCompare tool using SES, and the ratio of the reads was computed, with reads binned into 50-bp bins. Fingerprint plots were used to evaluate IP enrichment over INPUT. Peaks were called using MACS2 [81] broad peak calling settings with the following conditions: - - gsize 32 900 000, - - extsize 200, - - nomodel, - -shift 250, - - qvalue 0.01. Peaks were called on merged replicates. Peaks within 2000 bp were merged to form the final peak list due to the broad nature of the signal using BedTool MergeBed. Due to differences in coverage between some replicates, some samples were downsized using SamtoolsView for subsequent plotting. Metaplots and heatmaps were generated using DeepTools ComputeMatrix and plotProfile or plotHeatmap. Unless otherwise stated, metaplots and heatmaps show data from merged replicates. An elbow plot was generated in R Studio on the output of ComputeMatrix (DeepTools) to select the optimal number of *k*-mean clusters by which to group the ChIP-seq signals (Fig. 2C and Supplementary Fig. S3E). For *Z*-scores, values were calculated per chromosome in R Studio using a custom script (available upon request). Normalized signal across all chromosomes is shown as supplementary data (Supplementary Data 2 and 3).

### Whole genome sequencing analysis

Sequence read quality was examined first by FastQC (Andrews, S (2010). https://www.bioinformatics.babraham.ac.uk/projects/fastqc/), and coverage depth was assessed by Samtools Depth [77]. Adapter sequences were trimmed off using fastp [82] (default setting), and the trimmed reads mapped to the *Leishmania* Lmj.F 67 reference genome (TriTrypDB.org) using Bowtie2 [78] –verysensitive – parameters. Duplicate sequences were ignored (MarkDuplicates Samtools), and reads were filtered to exclude reads below MapQ [83] of 30 (“-q 30 -f 0 × 2 -F 0xf04”). ‘Shade-regions’ were removed from the downstream analysis (regions of genes with high copy number as described [45]). Principle Component Analysis (PCA) plots and Spearmen correlations were performed to examine replicate similarity and reproducibly. Two independent biological replicates were performed. Coverage files were generated using DeepTools [80] bamCoverage function (–binSize 1000 –normalizeUsing RPKM –scaleFactor 1.0 –extendReads –ignoreDuplicates –centerReads –minMappingQuality ‘30). Variants were called using FreeBayes v.1.3.1 [84] using the following parameters: –min-mapping-quality 30, –min-base-quality 20, –min-supporting-allele-qsum 0, and –genotype-variant-threshold 0. All other settings were default. Variants were filtered using VCFtools [85] VCFfilter as follows: -f ‘PAIRED > 0.8’ -f ‘ODDS > 10’ -f ‘QUAL > 30’ -f ‘DP > 10’ -f ‘DP > 10’ -f ‘MQM > 30’ -f ‘AO > 5’ -f ‘AF > 0.01’). VCF–VCF intersect was then used to find variants unique to each sample to isolate unique variants that result from exposure to HU or after serial passage. VCFfilter was then used to separate SNPs and INDELs. The number of SNVs or INDELs was first normalized to chromosome size, and then SNVs/INDELs were calculated per kb. Two independent experiments were performed.

### Data presentation, statistical analysis, and software usage

Figures were organized in MS PowerPoint® and BioRender (biorender.com). Data were visualized using Prism v10 (GraphPad), R, and RStudio (www.r-project.org; R Development 2010). ChIP-seq and whole genome data were examined in IGV as stated. All statistical analysis was performed in R or using GraphPad Prism. Statistical tests used are as described in the corresponding figure legends. MS Excel (Microsoft®) was used to prepare data tables. Some sequence data analysis was performed using the Galaxy server (usegalaxy.org/usegalaxy.eu) [86], with the remainder performed on internal servers. Flow cytometry data were analysed using FlowJo v.10 (https://www.flowjo.com). Raw data underlying each figure (where applicable) are shown in Supplementary Table S2.

## Results

### *Leishmania* RPA1 forms nuclear foci under replication stress

**Figure 1.**
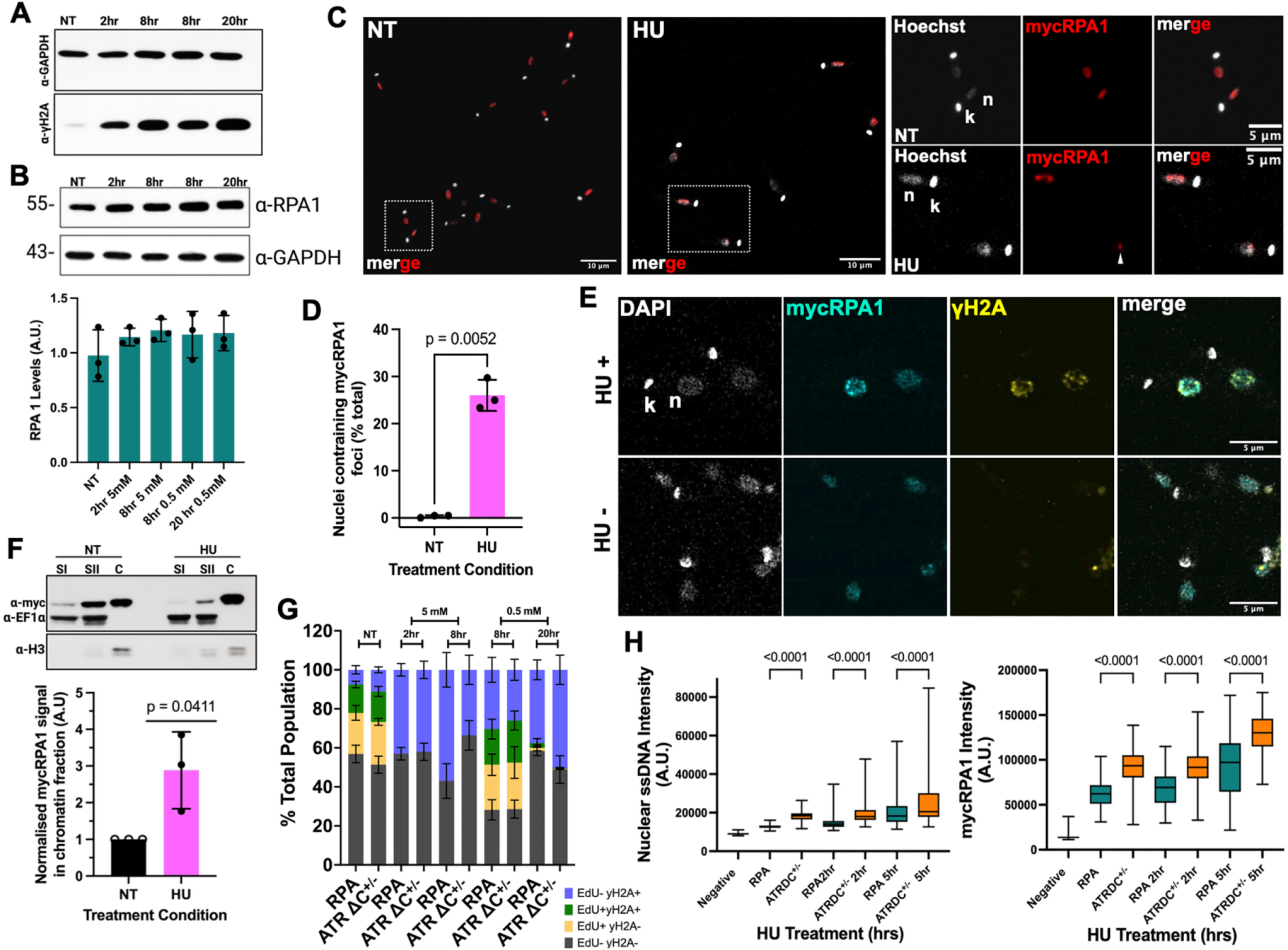
RPA1 forms nuclear foci in the presence of replicative stress. (**A**) Immunoblot showing yH2A signal after exposure to HU at two concentrations (0.5 and 5 mM) for 2 and 8 h (5 mM) and 8 and 20 h (0.5 mM). The membrane was probed with Trypanosome anti-yH2A antiserum and anti-GADPH (loading control), *n* = 2 independent biological experiments. (**B**) Immunoblot showing RPA1 signal after exposure to HU at two concentrations (0.5 and 5 mM) at the times shown in the figure. The membrane was probed with anti-LmRPA1 and anti-GADPH (loading control). The graph below shows the RPA1 signal quantified relative to the loading control (displayed in arbitrary units; AU), *n* = 3 independent biological experiments. (**C**) Representative indirect immunofluorescence images of *L. major* parasites expressing mycRPA1 in the absence (left) or presence (right) of RS induced by the addition of 5 mM HU for 10 h. mycRPA1 was probed for using anti-myc antiserum, and Hoechst was used to visualize the nuclear and kinetoplastid DNA. NT = non treated, dashed white boxes indicate the regions enhanced from the field of view. *k* = kinetoplast, *n* = nucleus. Scale bar as detailed. Images were captured on a Zeiss Multiphoton confocal microscope. (**D**) The percentage of mycRPA1 cells with nuclear RPA1 foci in the absence and presence of RS (5 mM HU for 10 h). Error bar = ± SD, *n* = 3 independent biological experiments. Significance was measured using Welch’s *t*-test *P* = .0052 (**E**) Representative indirect immunofluorescence images of *L. major* parasites expressing mycRPA1 in the presence (above) or absence (below) of RS following exposure for to 5 mM HU for 10 h. Trypanosome anti-yH2A antiserum was used to mark regions of DNA damage and/or RS. mycRPA1 was probed for using anti-myc antiserum, yH2A signal, and DAPI was used to visualize the nuclear and kinetoplastid DNA. *k* = kinetoplast, *n* = nucleus. Scale bar = 5 μm. Images were captured on a Leica Stellaris microscope. (**F**) Chromatin extraction was performed on mycRPA1 cells ± 5 mM HU for 4 h. Histone H3 (H3) was used to confirm isolation of the chromatin fraction, and EF1alpha was used as a loading control for all other fractions. Anti-myc antiserum was used to detect mycRPA1. The intensity of the myc signal from chromatin fraction was quantified in the absence and presence of HU. The graph shows the relative intensity of Myc signal in the HU-treated cells relative to controls (NT). Signals are shown as AU. Error bars = ± SD, *n* = 3 independent biological experiments. Significance was measured using a one sample *t*-test *P* = .0411. (**G**) Cells in the presence or absence of HU for 2 and 8 h (5 mM) and 8 and 20 h (0.5 mM) were pulsed EdU for 30 min and co-stained with yH2A. Cells were then categorized as stated in the figure. The graph shows the percentage of the total population for each category. Significance was measured using a two-way analysis of variance using Šídák’s multiple comparisons test. Error bars = ± SEM, *n* = 3 independent biological experiments, >200 cells counted per time point. (**H**) Quantification of nuclear ssDNA intensity (left) and mycRPA1 (right) intensity in mycRPA1 and ATRΔC ± cells. Intensity is expressed as AU. Significance was measured using a Kruskal–Wallis test followed by Dunn’s multiple comparisons test, *P* < .0001. Representative of *n* = 2 independent biological experiments, >150 cells per condition. Signal intensity was quantified using Fiji (ImageJ), and untagged Cas9T7 cells were used as negative controls (∼50 cells quantified). Created in BioRender. Black J.A. (2026) https://BioRender.com/y9vaxkr

To investigate whether RPA1 responds to RS in *L. major*, we performed immunoblotting on total protein extracts from cells exposed to either acute (5 mM; 2 and 8 h) or chronic (0.5 mM; 8 and 20 h) exposure to HU, which inhibits dNTP synthesis and induces RS [87], and probed for RPA1 (using *L. major* anti-RPA1) and yH2A (using *T. brucei* anti-yH2A) [69]. Both acute and chronic HU treatments increased yH2A levels relative to controls (Fig. 1A and Supplementary Fig. S1A), suggesting enhanced DNA damage due to RS. However, the total protein levels of RPA1 remained unchanged compared with controls (Fig. 1B and Supplementary Fig. S1B). We hypothesized that under RS, the RPA complex reorganizes into nuclear foci, akin to the behaviour of RPA after exposure to DNA damage in *T. brucei* [65, 66, 69] and *T. cruzi* [67]. To investigate RPA1 foci formation in *L. major* cells, we generated a 3×Myc-RPA1 cell line (mycRPA1) by fusing three copies of the Myc epitope to the N-terminus of RPA1 (Supplementary Fig. S1C). PCR confirmed integration of the tag into the endogenous locus (Supplementary Fig. S1C), and immunoblotting with anti-Myc (Supplementary Fig. S1D; left) and anti-RPA1 (Supplementary Fig. S1D; right) antibodies verified the expression of the fusion protein at the expected molecular mass (∼56 kDa). mycRPA1 cells did not show any significant growth or cell cycle defects relative to controls (Supplementary Fig. S1E and F), suggesting expression of the fusion protein does not perturb parasite proliferation.

In unperturbed mycRPA1 cells, indirect IFA revealed a diffuse Myc signal localizing exclusively at the location of the larger DAPI signal from the cell nucleus (Supplementary Fig. S1C; left panel; Supplementary Fig. S1G shows untagged controls), confirming that *L. major* RPA1 is a nuclear protein. Following exposure to HU (5 mM) for 10 h, we observed distinct nuclear foci of Myc signal in ∼25% of the cells (Fig. 1D, *P* = .0052), compared to <1% in untreated cells (Fig. 1D), suggestive of enhanced recruitment of RPA1 to the chromatin. We additionally confirmed these RPA1 foci localize in proximity to foci of yH2A in the nucleus of *Leishmania* parasites treated with 5 mM HU for 10 h to induce RS (Supplementary Fig. S1E and H shows Cas9 T7 untagged cells). To confirm the RPA1 fusion protein becomes chromatin-enriched under RS, we performed chromatin fractionation with and without HU treatment. Following acute HU treatment, we observed a ∼3-fold increase (*P* = .0411) in Myc signal within the chromatin fraction, whilst signal in other soluble fractions decreased (Supplementary Fig. S1F and I). These data reveal that RPA1 in *L. major* becomes enriched on chromatin and forms nuclear foci under RS.

### ATR-deficiency enhances replication stress in *Leishmania*

Accumulation of RPA-coated ssDNA promotes ATR activation in response to RS [2, 6]. Given ATR inhibition leads to excessive origin firing and accumulation of ssDNA in human cells [9], we investigated the dynamics of RPA1 in an ATR-deficient mycRPA1 cell line. One allele of ATR was truncated in mycRPA1 cells by deleting the C-terminal proportion of the ORF encoding the kinase domain, generating the cell line 3×MycRPA1ATRDC± (referred to as ATRΔC±) (Supplementary Fig. S2A). Truncation of one allele of ATR in the ATRΔC± cells was confirmed by PCR (Supplementary Fig. S2B) and Southern blotting (Supplementary Fig. S2C). We found no significant alteration to parasite proliferation in ATR-deficient cells, nor enhanced sensitivity to HU (Supplementary Fig. S2D and E). We next tested for alterations to DNA replication in ATRΔC± cells in the presence and absence of RS induced by HU. mycRPA1 and ATRΔC± cells were grown in the absence or presence of HU for 2 and 8 h (5 mM) or 8 and 20 h (0.5 mM). Cells were then pulsed for 30 min with EdU. EdU was detected using Click-IT® technology, and RS and DNA damage were detected using anti-Trypanosome yH2A (Supplementary Figs S1G and S2F). Cells were then categorized as follows: ‘EdU- yH2A-’ (no DNA replication detected, damage and RS negative), ‘EdU + yH2A-’ (actively replication, damage and RS negative), ‘EdU + yH2A+’ (actively replication, damage and RS positive), and ‘EdU- yH2A+’ (no DNA replication detected, damage and RS positive). No significant differences were observed between mycRPA1 and ATRΔC± cells except after an 8 h treatment with 5 mM HU, in which the number of yH2A-positive cells were significantly reduced in ATRΔC± cells (*P* < .0001), suggesting that ATR-deficient cells may fail to sustain a response to RS after prolonged exposure. To support these data, we next asked if ATRΔC± cells accumulate ssDNA by assessing ssDNA levels by IFA (Supplementary Figs S1H and S2G) through the detection of incorporated 5-iodo-2-deoxyuridine (IdU; a thymidine analogue) under native conditions. Additionally, we examined for alterations to RPA1 dynamics in ATRΔC± cells. In the absence of HU-induced RS, we detected a significant increase in both nuclear ssDNA (left graph) and RPA1 (right graph) intensity in ATRΔC± cells relative to mycRPA1 cells (Supplementary Fig. S1H; left). After HU exposure, the intensity of both ssDNA and RPA1 signal significantly increased (*P* ≤ .0001) in ATRΔC± cells compared with controls at 2 and 5 h of 5 mM HU (acute). Altogether, these findings suggest that impaired ATR function enhances RS in *L. major*, even in the absence of HU.

### Genome-wide mapping of yH2A, RPA1, and RPA1 under ATR deficiency in unperturbed *L. major* reveals RPA1 at sites of DNA replication initiation

To delineate the RSR in *Leishmania*, we first mapped the genome-wide distribution of RPA1 in asynchronously dividing promastigotes by ChIP-seq, targeting the 3×MycRPA fusion protein expressed in both mycRPA1 and ATRΔC± cells using anti-Myc antiserum. We also investigated the global dynamics of yH2A using anti-yH2A antiserum, aiming to identify sites of pronounced RS and/or DNA damage [15]. Additionally, we examined the location of the 9-1-1 complex to identify damaged RFs and DNA breaks under RS conditions [14, 88]. HUS1 and RAD9 were used as proxies for the 9-1-1 complex. For RAD9 and HUS1, ChIP-seq experiments were performed in a cell line expressing 12×Myc epitope fused to the C-terminus of RAD9 or HUS1 (RAD9myc and HUS1myc [73]). Two independent biological experiments were performed for each 3×Myc-RPA1-expressing cell line and for the yH2A ChIP-seq, with good reproducibility between replicates in untreated cells (Supplementary Fig. S3A–C). Data pertaining to RAD9 and HUS1 are provided in Supplementary Data 1. Whole chromosome plots for untreated cells are provided in Supplementary Data 2.

**Figure 2.**
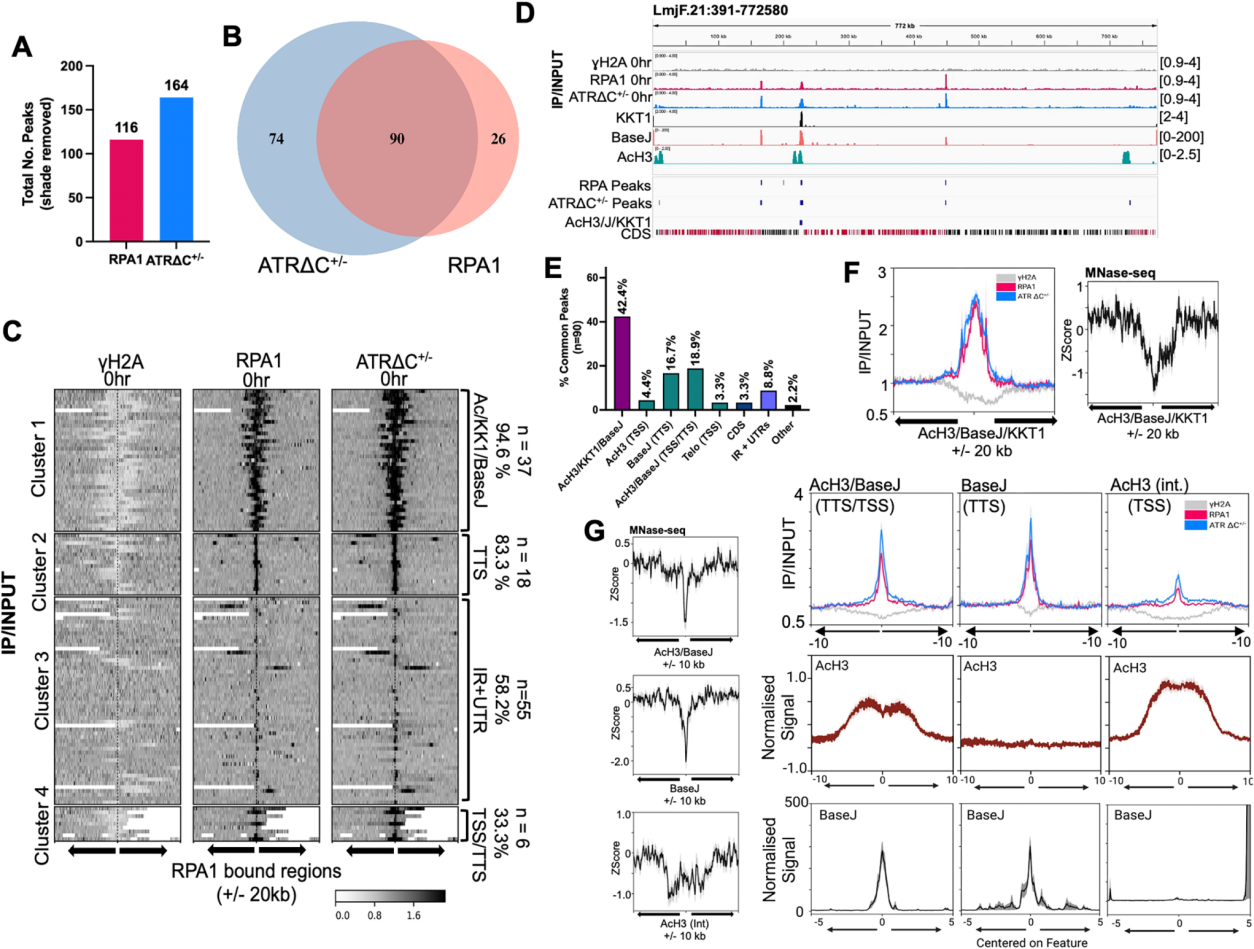
Genome-wide mapping of yH2A and RPA1 dynamics in unperturbed cells. (**A**) The total number of peaks for RPA1 identified using MACS2. Peaks were filtered for quality (>20) and removed if associated with regions of high coverage (‘shade regions’). (**B**) RPA1, in ATR-competent and ATRΔC± cells, co-occupy 90 genome regions in unperturbed cells. (**C**) RPA1-bound sites were identified using MACS2, and then the signals for yH2A, RPA1, and RPA1 (in ATRΔC±) were centered on these sites ±20 kb up- and downstream of the regions. The colour white on the heatmap indicates no signal. *K*-means clustering was used to group sites of similar signal together, and Bedtools intersect was used to locate the genomic features associated with these RPA1-bound sites. (**D**) Location of yH2A, RPA1, and RPA1 in ATRΔC± cells. ChIP-seq signals shown are mapped to a representative chromosome, LmjF.23. Enriched signals are scaled as stated. Tracks were viewed using IGV. The AcH3/KKT1/BaseJ site is denoted by a blue box, and CDSs by red (+ve strand) or black (−ve strand) boxes. Peaks from MACS2 for RPA1 and RPA1 in ATRΔC± are shown below (blue boxes). (**E**) The genomic features associated with these 90 common peaks are shown as a percentage of the total number of common peaks. (**F**) Metaplot analysis depicts enriched signal for yH2A and RPA1 signals centered on AcH3/KKT/BaseJ sites ±20 kb. Immunoprecipitated samples were normalized to their corresponding input samples. Nucleosome signal (as a normalized *Z*-score) at AcH3/KKT/BaseJ sites is shown in the metaplot on the right. One representative experiment for yH2A is shown. For RPA1, the signal reflects merged replicates. (**G**) Metaplot analyses depict enriched signal for yH2A and RPA1 signals centered on sites enriched for AcH3 (transcription initiation - chromosome cores), BaseJ (transcription termination), and AcH3/BaseJ (transcription initiation and termination) ±10 kb. The BaseJ signal is shown as ±5 kb. One representative experiment for yH2A is shown. For RPA1, the signal reflects merged replicates. TTS/TSS = transcription termination and start site, TTS = transcription termination site, TSS = transcription start site. Created in BioRender. Black J.A. (2026) https://BioRender.com/y5ux810

RPA1 ChIP signals from mycRPA1 and ATRΔC± cells strongly correlated (Pearson coefficient: 0.88; Supplementary Fig. S3D), and additionally with RAD9 ChIP signal from RAD9myc cell line (Pearson coefficient: RAD9 with RPA1: 0.88, RAD9 with ATRΔC±: 0.92; Supplementary Data 1), suggesting a similar enrichment pattern. Peaks called using MACS2 were then filtered to exclude regions of high coverage (‘shade areas’ [45]), resulting in the identification of 116 RPA1 peaks in mycRPA1 cells and 164 RPA1 peaks in ATRΔC± cells (Fig. 2A). The ~1.4-fold increase in RPA1 peaks in ATRΔC± cells compared to the mycRPA1 cell line aligns with the increased ssDNA signal observed in earlier data (Fig. 1H and Supplementary Fig. S2G), indicating heightened RS and/or damage when ATR is deficient. We next centered the ChIP signal from RPA1-enriched regions in mycRPA1 (defined by MACS2) and analysed the profile of yH2A and RPA1 in ATRΔC± cells in relation to these RPA1-bound sites using *k*-means clustering to group regions with similar signal patterns for γH2A and RPA1. Consequently, we identified four distinct signal clusters (Fig. 2C and Supplementary Fig. S3E). In cluster 1, which exhibited the strongest and broadest RPA1 enrichment, ~94% of the regions overlapped with the previously mapped early-S phase DNA replication initiation sites (AcH3/BaseJ/KKT1 sites) [37, 42], suggesting that one of the major sites of RPA accumulation is coincident with single DNA replication origins (early-S origins) detected in S-phase in *L. major*. These sites also showed co-enrichment with RAD9 and HUS1 (Supplementary Data 1), indicating RS and/or DNA damage at DNA replication initiation sites. We found no evidence of yH2A accumulation upstream or downstream of the early-S origins (Supplementary Fig. S2C and D). Enriched regions in cluster 2 primarily matched TTSs, with ~83% of the enriched regions overlapping with sites decorated with BaseJ alone (TTSs) or by both BaseJ and AcH3 (transcription termination and initiation sites TSS/TTS). SNS-seq predicted DNA replication initiation at TTSs that was not detected by MFA-seq [45]; since these sites accumulate both RPA1 and RAD9 (Supplementary Data 1), it is more likely that RS, and not DNA replication initiation, is being detected, suggesting that TTSs are pronounced sites of RS. In cluster 3, which exhibited the weakest enrichment signals, ~59% of the regions overlapped with various other genomic features, including inter-CDS sites and UTRs, indicating wider sites of DNA instability and/or repair, which could be associated with trans-splicing activity and/or polyadenylation. These regions also accumulate RNA:DNA hybrids (R-loops) in both *Leishmania* [89] and *T. brucei* [90], and likely experience elevated levels of ssDNA formation. Lastly, in cluster 4, ∼33% of these regions coincided with BaseJ and AcH3 (transcription termination and initiation sites/TSS/TTS), appearing broader than regions assigned to cluster 2.

Consistent with the data in Fig. 2C, nearly all the common peaks (90) identified in both mycRPA1 and ATRΔC± cells (Supplementary Figs S2E and S3F) were located at the boundaries of PTUs. Specifically, 42% of these peaks (38) were associated with early-S origins. Additionally, 35% (32) were found at sites of transcription termination (head-to-tail and convergent SSRs). Only ~7% of the common peaks localized to sites of transcription initiation, either within the interior of chromosomes (divergent SSRs, 4.4%; Fig. 2E) or at subtelomeres (AcH3 Telo, 3.3%; Fig. 2D). Unique RPA1 peaks were identified in mycRPA1 (26 peaks) and ATRΔC± cells (74 peaks). In support of the increase in ssDNA signal associated with ATR deficiency, we examined yH2A and RPA1 signal enrichment across the unique peaks from this dataset (Supplementary Fig. S3G). Consistent with enhanced ssDNA formation, the metaplots and heatmaps shown an enrichment of RPA1 at these regions in ATR-deficient cells when compared with RPA1 in ATR-competent cells. Unique RPA1 peaks were primarily associated with intergenic regions (IR; Supplementary Fig. S3F); however, in ATRΔC±, these unique peaks were associated with various genomic features (Supplementary Fig. S3H), including intergenic regions and gene coding sequences, potentially reflecting spontaneous genome instability events across the genome. The majority (∼46%) coincided with CDSs, suggesting that ATR signalling is required to prevent damage accumulation in gene coding regions. A further ∼22% were associated with transcription initiation regions (TSS).

Metaplot analyses confirmed the broad enrichment of each protein at the early-S origins (Fig. 2F and Supplementary Data 1), with RPA1 signal from mycRPA1 and ATRΔC± cells largely overlapping, suggesting that ATR deficiency does not affect RPA1 accumulation at early-S DNA replication initiation sites in unperturbed cells. We noted a reduction in yH2A ChIP signal at the center of these sites, and little accumulation ±20 kb of the regions, likely because these DNA replication initiation sites are nucleosome-depleted (Fig. 2F; right), preventing the generation of yH2A. In contrast, metaplot analyses of the other SSRs revealed sharp peaks for RPA1 and RAD9 (Supplementary Data 1), but not yH2A, at sites where transcription initiates and/or terminates (AcH3/BaseJ or BaseJ only sites), with markedly reduced peak sizes at transcription initiation sites (marked by AcH3) (Fig. 2G). No significant accumulation of either RPA1 or RAD9 was detected across the subtelomeres (Supplementary Fig. S3H and I; Supplementary Data 1). However, a modest increase in RPA1 in ATRΔC± cells and RAD9 signal was observed at subtelomere-proximal transcription initiation sites (Supplementary Fig. S3I; Supplementary Data 1).

These data reveal that in the absence of exogenous RS, RPA1 and RAD9 predominantly accumulate at PTU boundaries, widely at early-S origins and more focused at TTSs, highlighting these regions as the primary locations of RS and/or DNA damage and repair that are clearly detectable in *Leishmania*. Additionally, whilst ATR deficiency had a minimal impact on asynchronously dividing cells, the marked increase of RPA1 at transcription initiation sites in ATRΔC± cells underscores these regions as damage ‘hotspots’ when ATR function is impaired. Given that ATR functions as a dimer [91], the observed defect in heterozygous cells suggests that the mutant protein likely exerts a dominant-negative effect, potentially by forming non-productive complexes with the wild-type counterpart.

### RPA1 accumulates bidirectionally from early-S origins under acute replication stress in *L. major*

**Figure 3.**
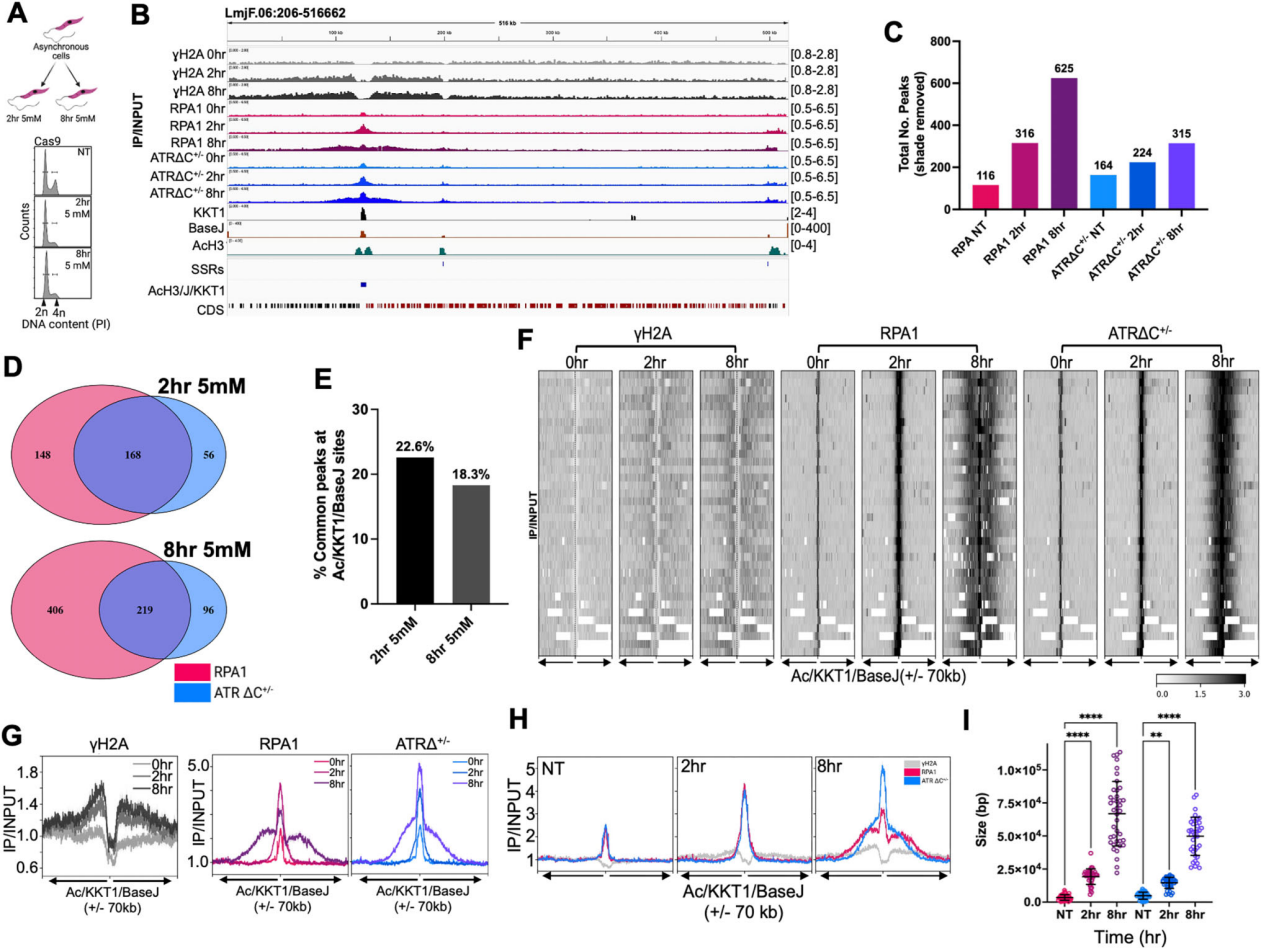
RPA1 accumulates at early-S origins in synchronized cells. (**A**) Schematic illustrating the experiment performed to synchronize cells. (Below) FACS analysis showing the DNA content of *Leishmania* in unperturbed cells and cells collected post-exposure to HU (5 mM) for 2 and 8 h. (**B**) Location of yH2A, RPA1, and RPA1 in ATRΔC± in the presence and absence of HU. ChIP-seq signals shown are mapped to a representative chromosome (LmjF.06) and shown in descending order of NT (untreated), 2 h 5 mM, and 8 h 5 mM. ChIP-seq samples were normalized to their corresponding input controls. Scale is as shown. Tracks were viewed in IGV. Polycistronic unit negative strand (black), polycistronic unit positive strand (red), early-S origin (blue box). Merged replicates are shown, except for yH2A, for which one representative experiment is shown. (**C**) Total number of peaks identified by MACS2 for each ChIP-seq dataset in the presence and absence of HU. Peaks overlapping with shade regions were removed. (**D**) RPA1 in ATR-competent and ATRΔC± cells co-occupy 168 genomic regions in cells 2 h post-HU exposure and 219 genomic regions at 8 h post-HU exposure. (**E**) The percentage of the common peaks associated with AcH3/KKT1/BaseJ (early-S origin sites). (**F**) Heatmap shows the enriched signal for yH2A, RPA1, and RPA1 in ATRΔC± centered on AcH3/KKT1/BaseJ sites ±70 kb for each chromosome. ChIP-seq samples were normalized to their corresponding input. Merged replicates are shown, except for yH2A, for which one representative experiment is shown. (**G, H**) Metaplot analyses depicting enriched signal for yH2A, RPA1, and RPA1 in ATRΔC± centered on early-S origin sites ±70 kb. Below shows the signal for each protein separately. ChIP-seq samples were normalized to their corresponding input controls. (**I**) The sizes of peaks associated with the early-S origin were quantified. Significance was calculated using a Kruskal–Wallis test followed by Dunn’s multiple comparisons test, ns = not significant, *** = 0.0038, **** = < 0.0001. Created in BioRender. Black J.A. (2026) https://BioRender.com/i0s97yo

In untreated *L. major* promastigotes, where most of the population is in G1 phase, approximately half of sites enriched with RPA1 signal localized to the previously mapped single site of DNA replication initiation on each chromosome [37, 42, 92], suggesting much of this observed signalling response primarily coincides with DNA replication activities. We next examined the localization of yH2A and RPA1 following HU treatment. Additionally, we examined RAD9 and HUS1 under these conditions. We first treated mycRPA1 and ATRΔC± cells with 5 mM HU for 2 and 8 h (Fig. 3A), which induces a cell cycle stall during G1/early-S. Under these conditions, we were unable to detect DNA synthesis by EdU labelling (Fig. 1G). We detected a strong correlation between replicates (Supplementary Fig. S4A and B). As in untreated cells, normalized ChIP signals from mycRPA1 and ATRΔC± after 2 and 8 h of HU exposure were highly correlated, indicating a consistent enrichment pattern (Pearson coefficient: 0.62–0.97; Supplementary Fig. S4C). The RAD9myc signal also strongly correlated with the RPA1 signal in ATR-competent and ATR-deficient cells after 2 and 8 h, supporting a similar pattern of enrichment (Pearson coefficient: 2 h RPA1 0.91, 2 h ATRΔC± 0.92, 8 h RPA1 0.74, 8 h ATRΔC± 0.91; Supplementary Data 1). Full chromosome signal plots are shown in Supplementary Data 3, and INPUT tracks are shown in Supplementary Fig. S4D.

Visual examination of the normalized ChIP-seq signal for yH2A and RPA1 revealed a broad accumulation of both H2A and RPA1 signal around predicted sites of DNA replication initiation in early-S (AcH3/KKT1/baseJ; Fig. 3B). By centring the ChIP-seq signals on RPA1-bound regions identified 2 and 8 h after HU exposure in mycRPA1 cells, we found that the ChIP signals largely remain in proximity to the RPA1-bound regions for 2 h, whereas at 8 h post-HU treatment, we detected a wider enrichment of RPA1 around the early-S origins in WT and ATRΔC± cells (Supplementary Fig. S4E). 9-1-1 complex signal remained more proximal to the early-S origins (Supplementary Data 1). At 2 h, MACS2 identified 316 peaks in mycRPA1 cells and 224 RPA1 peaks in ATRΔC± cells (Fig. 3C), after filtering. This approximately threefold increase in RPA1 peaks in mycRPA1 cells compared to untreated controls (Fig. 3C) indicates a significant enhancement of RS following HU exposure. In contrast, ATRΔC± cells showed a smaller increase (∼30%) in RPA1 peaks. After 8 h of HU exposure, we observed 625 RPA1 peaks in mycRPA1 cells and 315 RPA1 peaks in ATRΔC± cells (Fig. 3C). The ∼50% increase in RPA1 peaks in mycRPA1 cells further highlights the active RSR in these cells.

We then analysed ChIP signals associated with previously mapped early-S origin regions. After 2 h of HU exposure, we identified 168 common RPA1 peaks in both mycRPA1 and ATRΔC± cells (Fig. 3D). After 8 h, 219 common RPA1 peaks were observed between these cells (Fig. 3D). Approximately 20% of these common peaks colocalized with early-S origins (Fig. 3E), as nearly all early-S origins were enriched for RPA1 in ATR-competent and ATR-deficient cells. We also noted an enrichment of 9-1-1 signal at these early DNA replication initiation sites (Supplementary Data 1). When compared to untreated cells (Fig. 2F), HU treatment induces a broader enrichment of factors to locations beyond the known sites of DNA replication initiation activity, which themselves robustly recruit these two RSR factors. A more detailed analysis of signal enrichment at the early-S origins (Supplementary Fig. S3B and F–H, Supplementary Data 1) revealed several key observations. First, as HU treatment progressed from 2 to 8 h, the RPA1 signal significantly extended from all early-S origins in both mycRPA1 and ATRΔC± cells. This increase in RPA1 signal on both sides of the predicted early-S origin indicates that RS, a known consequence of HU exposure, is occurring. Second, RAD9 signal was also present around the early-S origins, but a modest broadening of the RAD9 signalling was only evident after 8 h of HU exposure, supporting that this accumulation of RPA may stimulate the activation of RAD9 and promote RAD9 recruitment to ssDNA in these parasites (Supplementary Data 1). Finally, whilst untreated cells exhibited no definable yH2A signal accumulation in proximity to the early-S origins (Fig. 2F), HU treatment led to a modest increase in signal accumulation distal to both sides of the early-S origins, mirroring that of the RPA1 signal and showing that HU treatment results in DNA damage around the early-S origins (Fig. 3B and F–H).

We next examined RPA1 signal in ATRΔC± cells under acute replication stress. First, we examined RPA1 signal at the early S-phase origins following 2 h exposure to HU (Fig. 3G and H). In ATRΔC± cells, we found that the distribution of RPA1 signal mirrored that of control cells, with a sharp peak localized to the center of the early S-phase origin. At 8 h post-HU exposure (Supplementary Fig. S3B, G, and H), similar to controls cells, the RPA1 signal in ATRΔC± cells formed a peak at the center of the early-S origin that expanded outwards bidirectionally. However, when compared visually to RPA1 signal from mycRPA1 cells under the same conditions, we noted that the RPA1 signal in ATRΔC± cells appeared less expansive on either side of the early S-phase origin. In all, though ATR is not crucial for DNA replication initiation, it is likely necessary for maintaining fork progression and/or stability (Fig. 3F–H). Analysis of early-S origin-associated peaks (Fig. 3I) revealed a significant increase in the average size of the RPA1 peaks (****P ≤ .0001). After 8 h of HU treatment, RPA1 peaks were ∼19 times broader compared to untreated cells and ∼3.5 times broader than at 2 h of HU treatment. Though a similar pattern emerged in ATRΔC± cells, with a significant enrichment in peak size (****P < .0001 and **P = 0.0038), the overall broad nature of the peak was reduced when compared to ATR-competent cells with ∼3 times broader at 2 h and ∼10 times broader at 8 h, when compared to untreated cells, highlighting a deficiency in RPA1 recruitment to ssDNA upon ATR deficiency. Altogether, we show that the single early-S origin previously identified by MFA-seq [37, 92] and DNAscent [42] is a major site of RPA and, putatively 9-1-1 accumulation, suggesting it is the most pronounced site of RS in each chromosome.

### Acute replication stress drives RPA1 accumulation at transcription initiation sites

**Figure 4.**
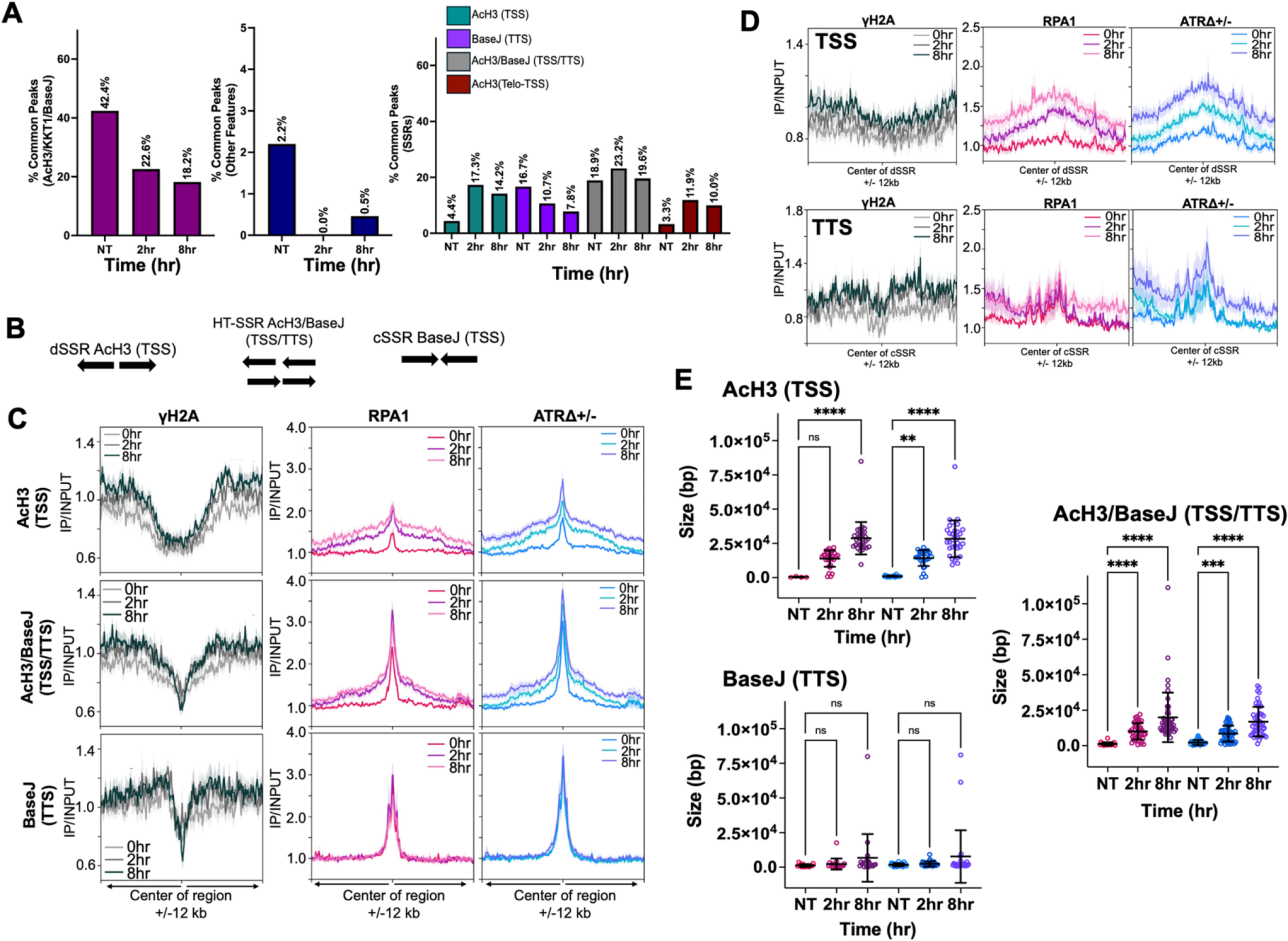
ATR-deficient cells accumulate RPA1 at SSRs associated with transcription initiation. (**A**) The percentage of the common peaks at 2 and 8 h associated with annotated genomic features. (**B**) Schematic illustration of SSR regions depicting the orientation of transcription. (**C**) Metaplot analyses depicting enriched signal for yH2A, RPA1, and RPA1 in ATRΔC± centered on sites enriched for AcH3 (transcription initiation - chromosome cores), BaseJ (transcription termination), and AcH3/BaseJ (transcription initiation and termination) ±12 kb in untreated (NT), 2, and 8 h. Merged replicates are shown, except for yH2A, for which one representative experiment is shown. (**D**) Metaplot analyses depicting enriched signal for yH2A, RPA1, and RPA1 in ATRΔC± centered on SSRs (dSSR and cSSR) as identified using nascent RNA sequencing (PRO-seq) ±12 kb in untreated (NT), 2, and 8 h. Merged replicates are shown, except for yH2A, for which one representative experiment is shown. TSS = transcription start site, TTS = transcription termination site. (**E**) The sizes of peaks associated with transcription-associated genomic regions were quantified. Significance was calculated using a Kruskal–Wallis test followed by Dunn’s multiple comparisons test, ns = not significant, **** = < 0.0001, ** = 0.0039, *** = 0.0005.

The chromatin binding profile of RS markers has been utilized previously to study events at unstable genomic regions, including sites at which DNA replication and transcription machinery collide [13]. We show that acute HU treatment leads to a broader localization of RPA1 compared to untreated cells. In untreated cells, RPA1 peaks were enriched at early-S sites of DNA replication initiation and at TTSs (Fig. 2). Fewer peaks of RPA1 were found at transcription initiation sites (Fig. 2). We next explored the basis of the broader signals detected following acute HU treatment (Fig. 3). Our analysis of MACS2 peak localization revealed that 2 h of HU exposure resulted in a 30-fold increase in peaks at transcription initiation sites compared with untreated cells, accounting for ~30% of the total common peaks. Additionally, around 23% (39 peaks) were found at both transcription initiation and termination sites, while only ~11% (18 peaks) were at transcription termination SSRs (Fig. 4A). A similar pattern emerged at 8 h post HU exposure, with ∼24% of common peaks at transcription initiation sites, ∼20% at both initiation and termination sites, and ∼8% at transcription termination-only sites (Fig. 4A). Overall, ∼52% of the peaks at 2 h and ∼44% at 8 h were associated with transcription initiation activity. The reduction in common peaks at TTSs after HU treatment likely reflects HU-induced slowing of DNA replication, leading to amelioration of transcription-replication conflicts at these locations. Conversely, HU exposure induces widespread RS, increasing the probability of unscheduled interactions between the DNA replication and transcription machineries [93]. In keeping with this, HU exposure increased the number of RPA1 peaks across multiple genomic regions (Supplementary Fig. S5). Though an indirect measure of replication-transcription conflicts, these data are suggestive of the widespread RS that could arise from these encounters.

Metaplot analyses (Fig. 4B–D and Supplementary Fig. S5) revealed pronounced RPA1 peaks at TTSs (locations of BaseJ or those mapped by Precision Run-On sequencing; PRO-seq [94]), with a modest accumulation of yH2A flanking the center of these regions. However, at transcription initiation sites (AcH3; Supplementary Fig. S4C and D), the peaks of RPA1 extended outward from a modest peak at the center (Supplementary Figs S4C, D and S5; RAD9 is shown in Supplementary Data 1). Minimal yH2A signal was observed flanking the center of these sites. At sites with both transcription initiation and termination activities (AcH3/BaseJ; middle panel), we observed broader peaks of RPA1 and RAD9 (Supplementary Data 1). Overall, the RPA1 peak sizes at TTSs (BaseJ) remained consistent across all samples and HU treatment time points (Fig. 4E). In contrast, RPA1 peaks at transcription initiation sites (AcH3) showed an increase in size at 2 h post-HU treatment; this increase in size became significant after 8 h of HU treatment compared to unperturbed cells (****P ≤ .0001). A similar, though less pronounced, pattern was observed at sites with both transcription initiation and termination activities, at which RPA1 signal in both control and ATR-deficient cells increased significantly relative to controls (****P = .0001, ***P = .0005). In ATRΔC± cells, some RPA1 peaks were broader than those identified in control cells (Fig. 4E), supporting enhanced DNA damage and/or increased RS at these regions in these cells. At subtelomeric sites, both 2- and 8-h 5 mM HU exposure induced an accumulation of RPA1 at chromosome ends (Supplementary Fig. S6). As these sites are nucleosome-depleted [45], we consequently noted a reduction of yH2A in proximity to the subtelomeric region. Little difference was observed between RPA1 signals across ATR-competent and ATR-deficient cells at 2 h; however, we noted a modest increase in RPA1 signal in ATR-deficient cells 8 h post-HU exposure (Supplementary Fig. S6B). When examining ChIP signal for RPA1 and yH2A across subtelomeric-associated transcription initiation sites (AcH3-Telo), we detected an accumulation of RPA1 with a modest accumulation of yH2A emanating from the region center outwards bidirectionally (Supplementary Fig. S6C). A similar pattern was observed for RAD9 (Supplementary Data 1). From 2 to 8 h the peaks of RPA1 significantly broadened relative to untreated cells (Supplementary Fig. S6D, *P* ≤ .0001), and that in ATRΔC± cells many RPA1 peaks were overall broader.

Taken together, whilst transcription initiation sites are not major stress loci in untreated cells, they exhibit broad and bidirectional accumulation of RPA1 and RAD9 in HU-treated cells, resembling the pattern seen at early-S origins of replication. Under conditions of acute DNA replication stalling, the increased levels of RPA1 and RAD9 accumulation indicate that sites of transcription initiation are also putative sites of RS, in common with the early-S origins and TTSs. Thus, all PTU boundaries in *Leishmania* appear to be locations of RS.

### During mid S-phase, an origin-like pattern of RPA1 accumulation emerges at transcription initiation sites

**Figure 5.**
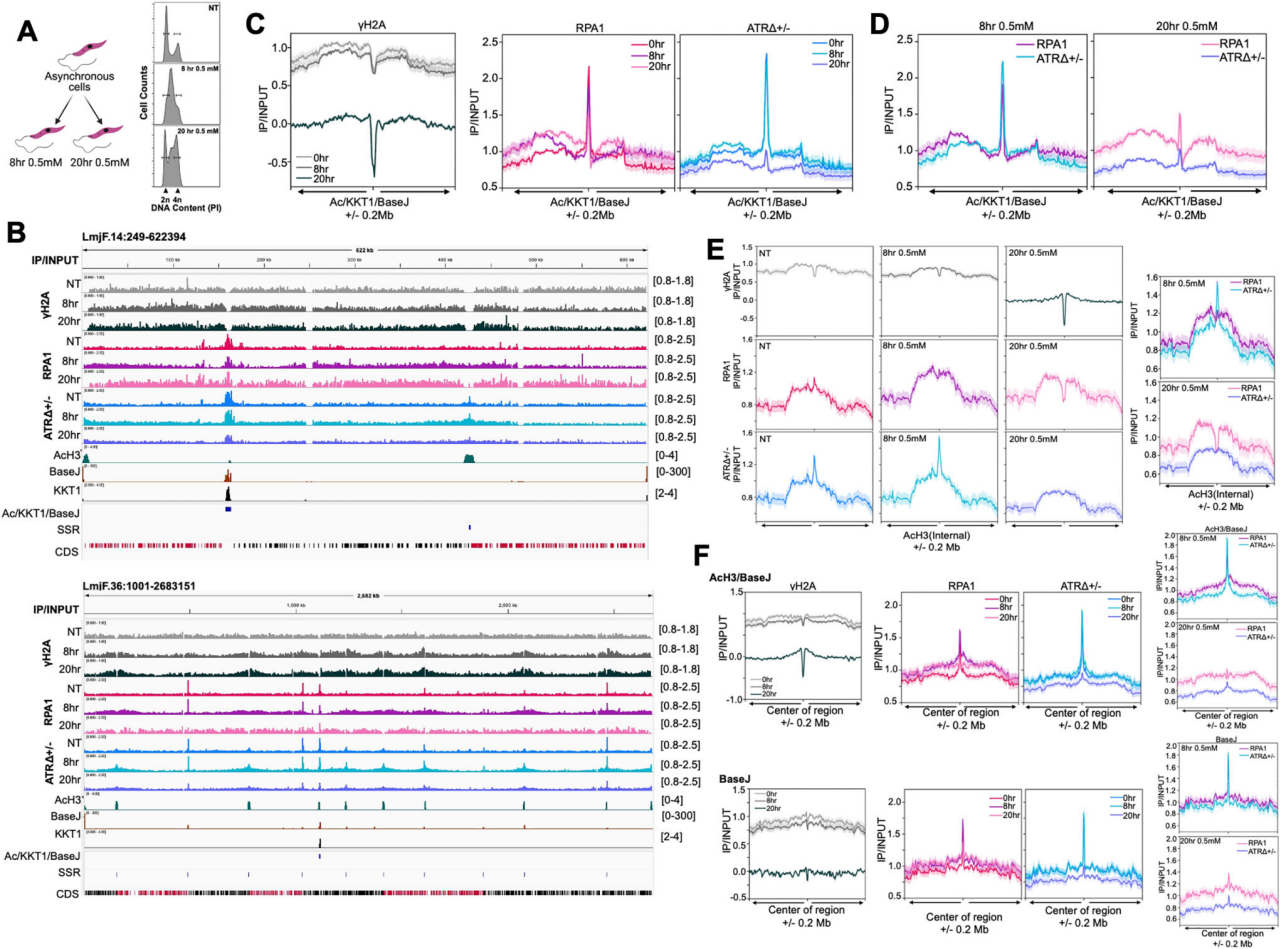
Low-dose HU exposure slows DNA replication and reveals ‘hotspots’ of RS at initiation and termination sites for transcription. (**A**) Schematic representation of the chronic HU treatment protocol. Cytometry plots below show representative FACS analysis of the DNA content of promastigotes treated with 0.5 mM HU for 8 and 20 h. (**B**) Location of yH2A, RPA1, and RPA1 in ATRΔC± cells. ChIP-seq signals shown are mapped to two representative chromosomes, LmjF.14 and LmjF. 36. Enriched signals are scaled as stated. Tracks were viewed using IGV. AcH3/KKT1/BaseJ site is denoted by a blue box, CDSs with red (+ve strand) or black (−ve strand) boxes. (**C**) Metaplot analyses depicting enriched signals for yH2A, RPA1, and RPA1 in ATRΔC± at early-S origins (±0.2 Mb). ChIP-seq samples were normalized to their corresponding input controls. One representative experiment for yH2A is shown. For RPA1, signal reflects merged replicates. (**D**) Metaplot analysis depicting enriched signals RPA1 and RPA1 in ATRΔC± cells centered on the early-S origins ±0.2 Mb. (**E**) Metaplot analyses depicting enriched signal for yH2A, RPA1, and RPA1 in ATRΔC± cells centred on sites enriched for AcH3 (transcription initiation; TSS; chromosome cores ± 0.2 Mb. Untreated cells (NT; left panel), 8 h (middle panel), and 20 h (right panel). One representative experiment for yH2A is shown. For RPA1, signal reflects merged replicates. ChIP-seq samples were normalized to their corresponding input controls. Individual metaplots are shown for each protein. Right metaplots depict enriched signals for RPA1 and RPA1 mapped in ATRΔC± centred on different AcH3 TSS sites as detailed—±0.2 Mb. Signal reflects merged replicates. ChIP-seq samples were normalized to their corresponding input controls. (**F**) Metaplot analyses depicting enriched signal for yH2A, RPA1, and RPA1 in ATRΔC± cells centred on sites BaseJ (TTS; transcription termination) and AcH3/BaseJ (TSS/TTS; transcription initiation and termination) sites. One representative experiment for yH2A is shown. For RPA1, signal reflects merged replicates. ChIP-seq samples were normalized to their corresponding input controls. Individual metaplots are shown for each protein. Right metaplots depict enriched signals for RPA1 and RPA1 mapped in ATRΔC± centred on AcH3/BaseJ (TSS/TSS) sights (above) and BaseJ (TTS) sites (below) as detailed—±0.2 Mb. Signal reflects merged replicates. ChIP-seq samples were normalized to their corresponding input controls. Created in BioRender. Black J.A. (2026) https://BioRender.com/6txbo06

Acute RS induced a distinct pattern of RSR factor accumulation at transcription initiation sites, like the pattern observed at early-S origins. Therefore, we sought to investigate what happens under mild RS conditions that are permissive for DNA replication and cell cycle progression (Fig. 1G). *Leishmania major* promastigotes were exposed to 0.5 mM HU to induce chronic RS for 8 and 20 h. We confirmed by FACS analysis an accumulation of cells in S phase after 8 h and in G2/M after 20 h (Fig. 5A). Under these conditions, where DNA replication from early-S origins will have progressed, we can explore the dynamics of the cell’s response to RS. ChIP-seq signal across each chromosome is shown in Supplementary Data 3. High reproducibility between most replicates was observed (Supplementary Fig. S7A and B) in treated cells. At 8 and 20 h, we observed a poor correlation between the RPA1 signal in mycRPA1 cells and ATRΔC± cells (Supplementary Fig. S7C and Supplementary Data 1). Taken together, this suggests that the pattern of RPA1 enrichment is altered in ATRΔC± cells exposed to chronic HU treatment (Supplementary Fig. S7B).

Centring the ChIP-seq signals on early-S origins revealed that after exposure to 0.5 mM HU for 8 h, the yH2A, RPA1, and RAD9 signals were primarily enriched in a relatively narrow region around the early-S origins, as observed in untreated cells. Additionally, the early-S origin-proximal bi-directional enrichment of RPA1 signal, within 70 kb upstream or downstream, seen at the 5 mM HU treatment, was not detected in cells treated for 8 h with 0.5 mM HU (Supplementary Fig. S7D and E). Instead, these cells exhibited a more distal, wider, and less intense enrichment of RPA1, within 0.2 Mb from the early-S origin site (Supplementary Fig. S5B and C). At 20 h (0.5 mM), we could detect a modest accumulation of RPA1 in ATR-competent cells only upstream and downstream of the early-S origin (Supplementary Fig. S5B and C; Supplementary Fig. S7D and E). Metaplots confirmed these similarities and differences around the mapped early-S origins (Fig. 5C), suggesting that the primary effect of chronic HU exposure is an accumulation of RPA at RFs that eventually progress bidirectionally from putative DNA replication initiation sites but are likely perturbed due to reduced levels of nucleotides. We found little difference in RPA1 signals between ATR-competent and ATR-deficient cells at 8 h; however, at 20 h, the accumulation of RPA1 in the ATR-deficient cells was notability reduced (Supplementary Fig. S5C and D).

Analysis of ChIP mapping beyond the early-S origins revealed that chronic HU treatment led to widespread accumulation of RPA1 across the genome, with a significant portion of the signal concentrated at PTU boundaries (Fig. 5B, E, and F; Supplementary Fig. S8A–C). A similar pattern was seen for 9-1-1 complex (Supplementary Data 1). At TTSs (BaseJ only), sharp peaks of RPA1 were detected at 8 h, indicating these regions are a consistent hotspots for RS and/or DNA damage (Supplementary Fig. S7F). As before, at 20 h, less signal was detected across all proteins. At transcription and termination sites (AcH3/BaseJ), sharp peaks of RPA1 were detected, along with a broadened signal extending towards the base of the peak across all cell lines (Supplementary Fig. S7G). These peaks became less pronounced at 20 h post-HU exposure. Notably, we detected broad peaks of RPA1 and yH2A, which were observed upstream and downstream, extending approximately equivalent distances from the transcription initiation sites (AcH3 or TSS; Fig. 5E; Supplementary Fig. S7H). Similarly, at 20 h, broad peaks of RPA1 and yH2A were detected; however, they were notably reduced when compared with peaks at 8 h, suggesting a reduced retention of each factor at the level of the chromatin. This pattern is reminiscent of the broad peaks identified after acute HU treatment at these AcH3-enriched sites, particularly at early-S origins (Fig. 3). Moreover, in ATR-deficient cells, we observed a very modest decrease in peak amplitude at 20 h compared to RPA1 in ATR-competent cells, suggesting that ATR could play a role in maintaining RF stability at these sites (Fig. 5E). At subtelomeric regions, we identified an accumulation of RPA1 under chronic RS conditions, albeit at a more modest level when compared to acute RS conditions, with a reduction in RPA1 signal in ATR-deficient cells (Supplementary Fig. S8). We found little accumulation of RAD9/HUS1 at these sites (Supplementary Data 1) and limited yH2A signal, likely due to the reduced nucleosome content within these regions. Though RPA is recruited to ssDNA at these sites, the limited recruitment of RAD9 suggests mild RS exposure may be insufficient to trigger the RSR.

Altogether, we suggest that slowing DNA replication via the addition of a low concentration of HU reveals that SSRs in *Leishmania* are sites of transcription-replication conflicts.

## Discussion

In *Leishmania*, and indeed all kinetoplastids, virtually all genes are transcribed from polycistrons. Consequently, RNA Polymerase must traverse long distances and, if transcription overlaps with S-phase, it is especially at risk from being impeded by DNA replication. This is the first study to provide a global map of RSR proteins in the absence and presence of RS in *Leishmania*, uncovering putative interactions between DNA replication and known sites of transcriptional activity in these organisms.

Our global map of RPA1 dynamics in the absence of HU highlighted sites of DNA replication initiation during early S-phase as pronounced sites of RS. In the immediate early-S origin centre, sharp peaks of RPA1 and RAD9 were detected, indicative of localized DNA damage. Under condition of acute RS, the sharp peaks of RPA1 and RAD9 persisted within the early-S origin centre and, additionally, bi-directional expansions of yH2A and RPA1 emerged, spread outwards from the early-S origin centre. For yeasts and metazoans, and *Leishmania*, the early-S origin region is commonly nucleosome-depleted [45, 95–97]; therefore, we were unable to map yH2A signal within the central portion. However, at 8 h (5 mM HU), the loss of yH2A immediately proximal to the early-S origin region expanded, indicating the chromatin is more open. These signal patterns began accumulating at 2 h post-HU exposure, suggesting this response is dynamic. The accumulation of yH2A and RPA1 suggests the presence of expanded ssDNA tracks, and therefore wider stalling of RFs:ssDNA accumulation is a common consequence of impaired DNA replication during which the DNA double strand is unwound but uncoupled from synthesis [2]. Under these conditions, RAD9 and HUS1 in *Leishmania* primarily localizes to early-S origins (Supplementary Data 1); however, expansion of the RAD9 signal is suggestive of 9-1-1 recruitment to the immediate vicinity of the early-S origin, perhaps stimulated by the increased RPA1 accumulation within the region as a consequence of enhanced RS (Fig. 3). The recruitment of RSR factors to the early-S origin, even in normally replicating promastigotes, suggests DNA replication initiation here induces particularly pronounced levels of RS in *Leishmania*, which is exacerbated after HU treatment. Likely, the prominence of RSR factors at these single sites in each chromosome reflects their constitutive activation during early S-phase and explains why they are so readily detected by both MFA-seq [37, 92] and DNAscent [42] as major sites of DNA replication initiation. As these are also sites of both transcription initiation and termination activities, their constitutive use as early-S origins will impose especially focused encounters between the transcription and replication machineries. Though leading DNA strand replication in *Leishmania* was inferred to move co-directionally with transcription [37], during lagging strand duplication, head-on collisions between the replisome and the transcription machinery may become more common, inducing DNA breaks; however, we lack strand-specific information from our ChIP-seq analyses to confirm this hypothesis.

Efficient, early origins in human cells are associated with enhanced mutagenesis [98], and mutagenic DNA replication initiation coincides with genome evolution, influencing gene expression. Indeed, a recent study in *Leishmania* confirms enhanced SNP accumulation at early-S origins, with later-replicating sites accumulating SNPs, but to a lesser extent [42]. As gene expression regulation in *Leishmania* is primarily post-transcriptional, the consequences of enhanced RS and likely DNA breaks, within early-S origins and other sites of DNA replication initiation, on the regulation of *Leishmania* gene expression are unknown. Could early-S origin instability explain in part the frequency of aneuploidy in these parasites, by altering the behaviour of the kinetochore interaction, thereby inducing missegregation of chromosomes? Enhanced levels of DNA breaks within early-S origins could increase the likelihood of chromosome fusions, recombination events, and genome instability, as reported frequently in DNA repair-defective *Leishmania* mutants [56, 99, 100]. In the ATR-deficient mutants, RPA1 tracks were highly reduced in length. Though under these conditions, ssDNA becomes exposed, our data suggest that these cells are defective in RPA1 recruitment to ssDNA and therefore subject to enhanced RF collapse. We directly tested for alterations to ploidy in Cas9 T7 parasites exposed to chronic RS (20 h 0.5 mM HU) after 10 serial passages; however, we were unable to detect significant ploidy alterations (Supplementary Figs S9 and S10A). We, however, noted that serially passaging cells following HU exposure significantly increased the number of SNVs and INDELS (insertions and deletions), relative to untreated controls, supporting our observations that HU exposure, and thus RS conditions in *Leishmania*, enhances genome instability (Supplementary Fig. S10B and C). Additionally, under acute HU-induced RS, we uncovered an enhanced accumulation of RPA1 peaks at SSRs, detecting a sharp peaks RPA1 confined to TTSs. The peak amplitude and shape remained largely consistent at 2 and 8 h post-HU exposure, suggesting these sites are constitutive sites of RS, irrespective of exposure length. In contrast, at sites of transcription initiation/termination or initiation only, we noted a sharp peak of RPA1 and RAD9 at the centre, which broadened outwards bidirectionally at 8 h. At sites of initiation only, the sharp peak at the centre reduced in amplitude when compared to termination and initiation sites. In both instances, the lack of yH2A signal accumulation and the broad loss of yH2A at the central region outwards suggests a more open chromatin environment (Fig. 4). We have not directly measured DNA replication under these conditions; however, these patterns of RSR enrichment at transcription initiation sites are reminiscent of the accumulation of these factors at the early-S origin, albeit more modestly. Moreover, increasing reduction of the yH2A signal at the immediate centre outwards suggests increasingly open chromatin, a feature of sequence consensus-free DNA replication initiation sites [97]. However, equally, these data could be explained by the fact that the activity of RNA polymerase is not inhibited by HU treatment. Thus, under RS, the transcription machinery can collide with HU-stalled DNA polymerase wherever it remains in the genome. As transcription start sites are the major sites of RNA polymerase loading, the accumulation of RSR factors to these regions may reflect this. Moreover, other modulatory factors can influence RPA accumulation. For instance, the local formation of R-loops can perturb the smooth progression of the replication machinery [101], locally inhibiting the generation of RPA-coated ssDNA gaps at the stalled forks. Thus, the observed RPA loading events in our study likely reflect the culmination of these interconnected regulatory networks rather than a simple consequence of dNTP exhaustion.

In contrast, at sites of transcription termination, the lack of expanded RPA1 and RAD9 signals suggests localized damage (discussed below in more detail). When we examined the RPA1 signal in ATR-deficient cells, we noted, in contrast to the early-S origin, an increase in peak size at both 2 and 8 h relative to RPA1 in ATR-competent cells. Thus, ATR may maintain the stability of the sites to prevent excessive ssDNA formation, in agreement with our recent data [102]. A limitation of our study is the inference of transcription initiation and termination activity from genome annotations derived from the independent datasets [39, 41, 49, 94]. Though we have not directly evaluated the impact of HU on transcription dynamics herein, our data demonstrates clear correlations between the accumulation of at least RPA1 signal at constitutive sites of validated transcription activity.

Chronic RS (8 h 0.5 mM HU) slowed but did not impede the progression of DNA replication (Fig. 1G) [103]. No broad enrichment of yH2A, RPA1, or RAD9 at the early-S origins was detected, only a sharp central peak at 8 h, which notably reduced at 20 h, suggestive of persistent RS within these sites during S-phase. Instead, RSR factors accumulate broadly and extend outwards from transcription initiation-exclusive SSRs. This pattern was absent at transcription termination-associated SSRs. Instead, sharp RPA1 were detected, and in keeping with this observation, we additionally found sharp RAD9 peaks (Supplementary Data 1) irrespective of HU exposure, indicating these sites are constitutive regions of instability and RS. In contrast to initiation sites of transcription, RNA polymerase is unloaded at sites of transcription termination and therefore should not be affected by HU treatment; however, in human cells, DNA replication terminates at sites of transcription termination [104], perhaps suggest that the instability detected within *Leishmania* could reflect the same process, though more work is required to test this. Given these cells are primarily mid-S phase at 8 h post-HU exposure (0.5 mM), the early-S origins have likely fired and progressed, explaining the loss of expanded, bidirectional peaks of yH2A and RPA1 at these sites. In contrast, following 20 h HU exposure (0.5 mM), the cells are primarily in G2/M, with RPA1 and yH2A signal markedly less defined compared with 8 h, appearing more broadly spread across the genome. Thus, most DNA synthesis has completed prior to cell division, and indeed, we detected limited DNA synthesis after cells were exposed to 20 h 0.5 mM HU (Fig. 1G). Therefore, we propose signals associated with DNA replication activities should diminish. The reduced RPA1 signals may suggest less ssDNA and RFs are present, and at this timepoint, we are likely detecting unresolved RS and DNA damage that may persist into the next cell cycle. In accordance, we observed persistent yH2A signal by western blotting (Fig. 1A) and IFA (Fig. 1G).

RS is associated with the firing of dormant origins in other eukaryotes to ensure DNA replication completion and compensate for stalled RFs if the constitutive origin firing becomes inadequate. Thus, the accumulation of RSR factors largely at transcription initiation sites under chronic RS conditions might provide further evidence that these loci could act as dormant origins, ensuring DNA replication completion under stress. Indeed, evidence for the firing of a single putative dormant origin under RS has been shown in the related pathogen *T. brucei* [105]. One possible benefit of placing a dormant origin at a transcription initiation site is the reduced possibility of head-on collisions with the transcription machinery, thereby limiting the formation of DNA damage at these sites [2]. The continuous presence of RAD9 and HUS1 (Supplementary Data 1) at these sites could be suggestive of enhanced RS and DNA breaks, suggesting these sites may coincide with enhanced mutagenesis. We found a mild reduction in peak amplitude in ATR-deficient cells, suggesting ATR is likely required to maintain the stability of these regions and highlights the continual importance of ATR in *Leishmania*.

*Leishmania* subtelomeric duplication appears compartmentalized, operating throughout the cell cycle and driven by RS-associated factors [37]. The clear accumulation of at least RPA1 signal within subtelomeric regions in both the presence and absence of RS is suggestive of a direct relationship between enhanced RS and subtelomeric regions. Moreover, subtelomere-proximal sites of transcription initiation show similar patterns of RPA1 that are suggestive of DNA replication initiation activity. Commonly, expanded gene families like the antigenic genes of pathogens are often located within the subtelomeres [106, 107]; thus, enhanced RS within subtelomeric regions may diversify multigene families; however, *Leishmania* subtelomeres are devoid of such genes, questioning the importance of enhanced RS within such sites in these parasites.

In conclusion, we provide the first global map of the RSR in *Leishmania*. We propose two major findings. First, the main sites of RS are PTU boundaries, where RNA polymerase is loaded and unloaded. Second, we observe limited accumulation of RSR factors within the PTUs, even following HU treatment. The alterations in RPA1 and yH2A signals at SSRs post-HU may suggest increased collisions resulting from DNA polymerase stalling, while RNA polymerase continues its loading and movement; however, these changes might also relate to DNA replication processes, such as the presence of putative ‘dormant’ origins at transcription initiation sites. One possible explanation for having a primary main origin per chromosome could be to minimize collisions between DNA replication and transcription at the few sites where their assembly and disassembly are synchronized. Furthermore, our findings may indicate the existence of previously unrecognized pathways that rapidly resolve conflicts between DNA replication and transcription within PTUs, ensuring continuous RNA polymerase movement and efficient gene expression.

## Supplementary Material

gkag754_Supplemental_Files

## Data Availability

Raw sequence reads for KKT1 ChIP were acquired from [43]. BaseJ and AcH3 coordinates were provided by J.D. Damasceno and originally acquired from [37, 39, 41, 108]. PRO-seq data from *L. major* were acquired from [94]. Nucleosome data were acquired from [45]. ChIP-seq and whole-genome sequence data generated in this study are accessible via the European Nucleotide Archive (ENA), accession number PRJEB83580 (ERP167172). This study would not have been possible without TriTrypDB.org [109, 110].

## Notes

Present address: Department of Biochemistry and Immunology, Ribeirão Preto Medical School, University of São Paulo, Brazil
