# Supplementary material for: Genome-wide mapping of replication stress response factors reveals the management of polycistronic transcription, replication initiation, and responses to replication stress in Leishmania
Source: Nucleic Acids Res. 2026 Aug 10;54(15):gkag754. doi: 10.1093/nar/gkag754 (PMC13454844; doi:10.1093/nar/gkag754)
Supplement: Supplementary file 1 [file gkag754_supplemental_files.zip › SupplementaryData1.pdf]

Data relating to Figure 2: RAD9 and HUS1

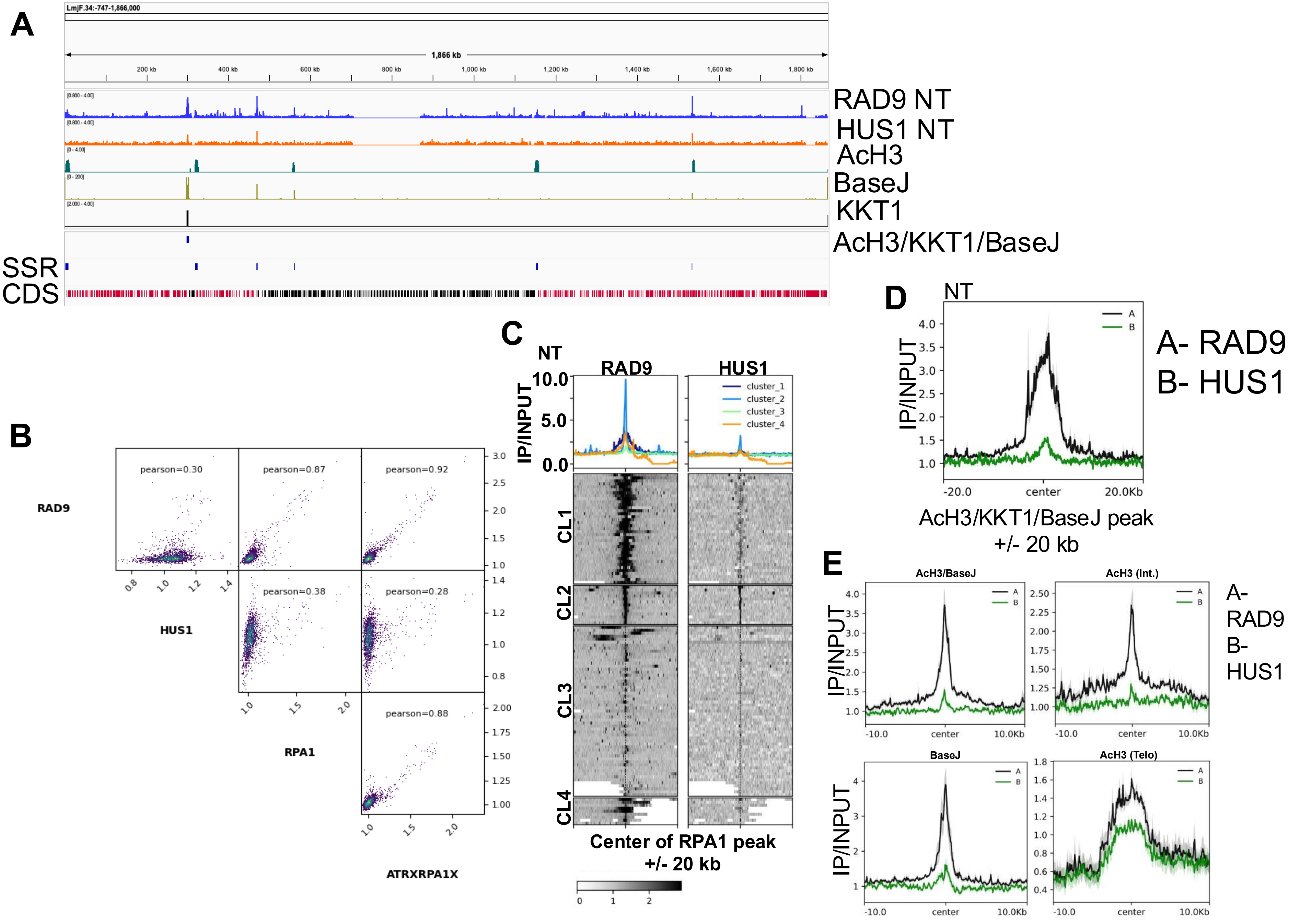

Data relating to Figure 2: RAD9 and HUS1

Legend

(A) IGV plot showing normalised ChIP-seq signal for RAD9 and HUS1 in untreated cells. IP samples were normalized to their corresponding INPUT control samples. Scale 0.8-4.0 for HUS1 and RAD9, 0-4 AcH3, 0-200 BaseJ and 2-4 KKT1. LmjF.34 is shown as a representative chromosome.
(B) Spearman coefficient correlation analysis to assess the global comparison of RAD9, HUS1, RPA1 in ATR-competent cells and ATRΔC+/- cells ChIP-seq signals
(C) Heatmaps show the enriched signals for RAD9 and HUS1 centered RPA1 peaks identified by MACS2 and clustered as per Figure 2 +/- 20 kb.
(D) Metaplot analysis showing the enriched signals for RAD9 and HUS1 centered on AcH3/KKT1/BaseJ (early origin) +/- 20kb. ChIP-seq samples were normalised to their corresponding input controls
(E) Metaplot analysis showing the enriched signals for RAD9 and HUS1 centered on AcH3 (internal), AcH3 (telo), AcH3/BaseJ and BaseJ sites +/- 10kb. ChIP-seq samples were normalised to their corresponding input control.

Data relating to Figure 3&4: RAD9 and HUS1

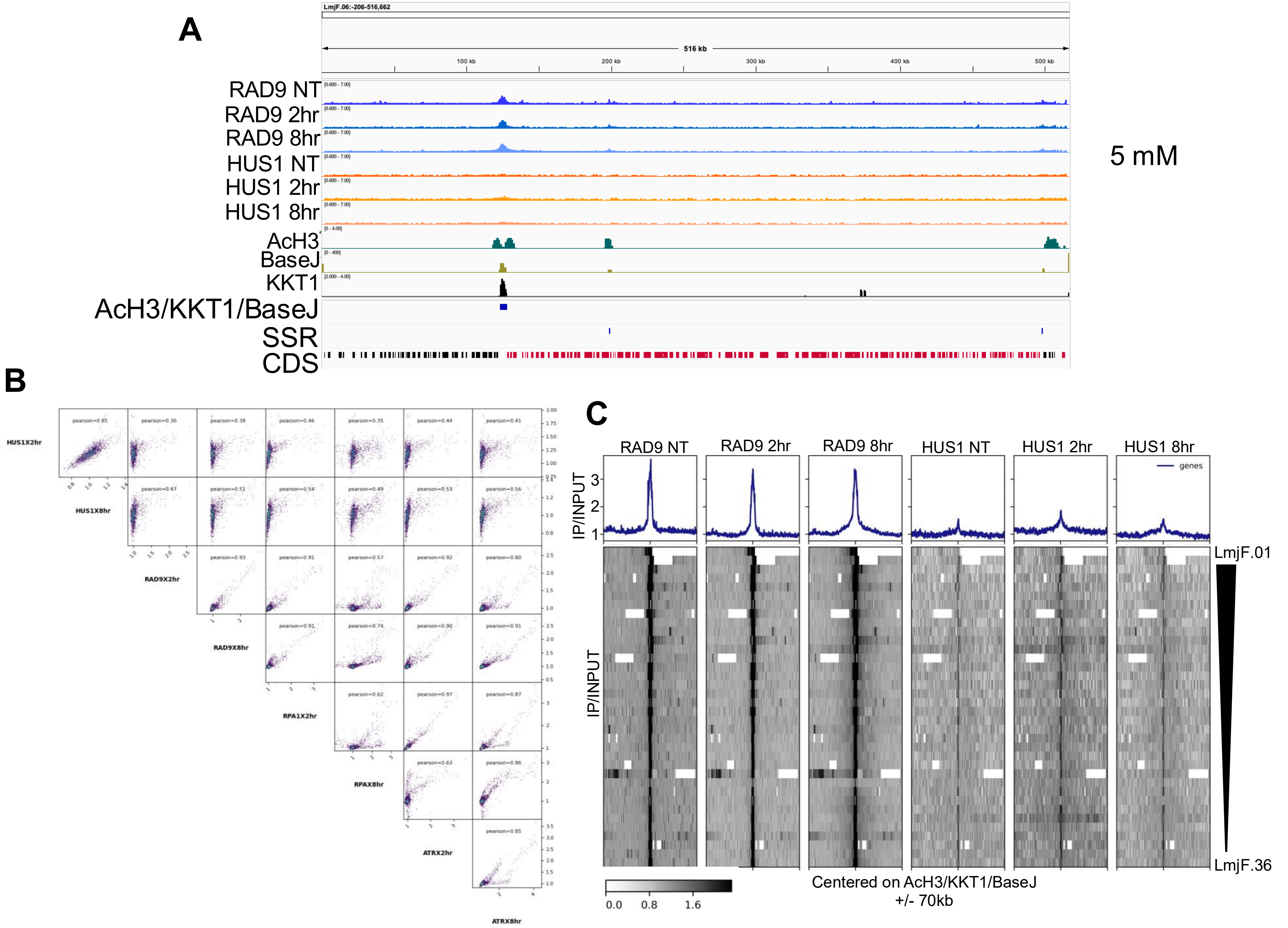

Data relating to Figure 3&4: RAD9 and HUS1

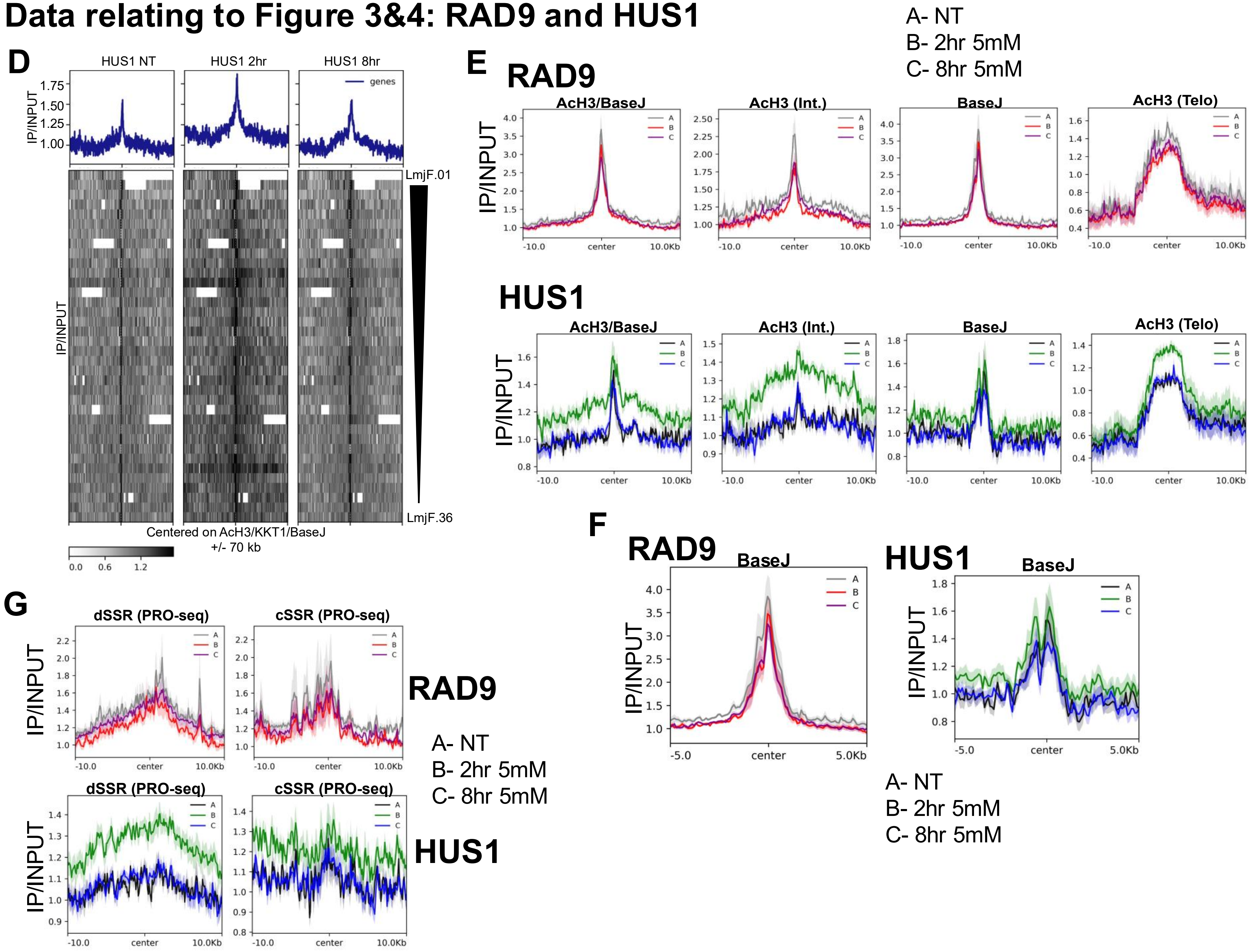

Data relating to Figure 3&4: RAD9 and HUS1

Legend

(A) IGV plot showing normalised ChIP-seq signal for RAD9 and HUS1 under acute RS. IP samples were normalized to their corresponding INPUT control samples. Scale 0.6-7.0 for HUS1 and RAD9, 0-4 AcH3, 0-400 BaseJ and 2-4 KKT1. LmjF.06 is shown as a representative chromosome.
(B) Spearman coefficient correlation analysis to assess the global comparison of RAD9, HUS1, RPA1 in ATR-competent cells and ATRΔC+/- cells ChIP-seq signals
(C) Heatmaps show the enriched signals for RAD9 and HUS1 centered on the early origin site +/- 70 kb.
(D) Heatmaps show the enriched signals for HUS1 centered on the early origin site +/- 70 kb.
(E) Metaplot analysis showing the enriched signals for RAD9 and HUS1 centered on AcH3, AcH3/BaseJ, AcH3 (Telo) and BaseJ sites +/- 10kb. ChIP-seq samples were normalised to their corresponding input controls
(F) Metaplot analysis showing the enriched signals for RAD9 and HUS1 centered on BaseJ sites +/- 5kb. ChIP-seq samples were normalised to their corresponding input controls
(G) Metaplot analysis showing the enriched signals for RAD9 and HUS1 centered on PRO-seq identified dSSRs and cSSRs +/- 10kb. ChIP-seq samples were normalised to their corresponding input controls

Data relating to Figure 5: RAD9 and HUS1

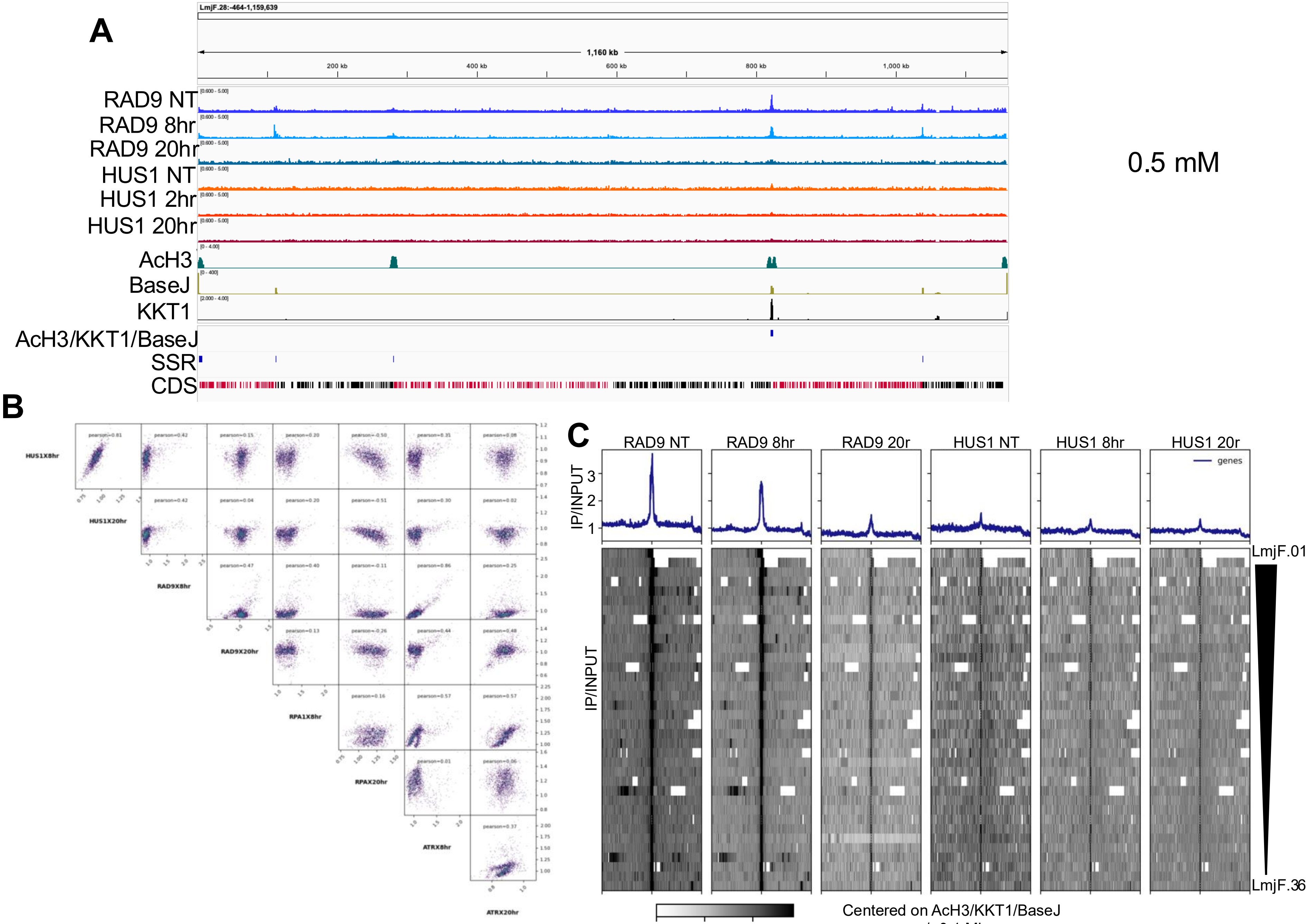

Data relating to Figure 5: RAD9 and HUS1

D

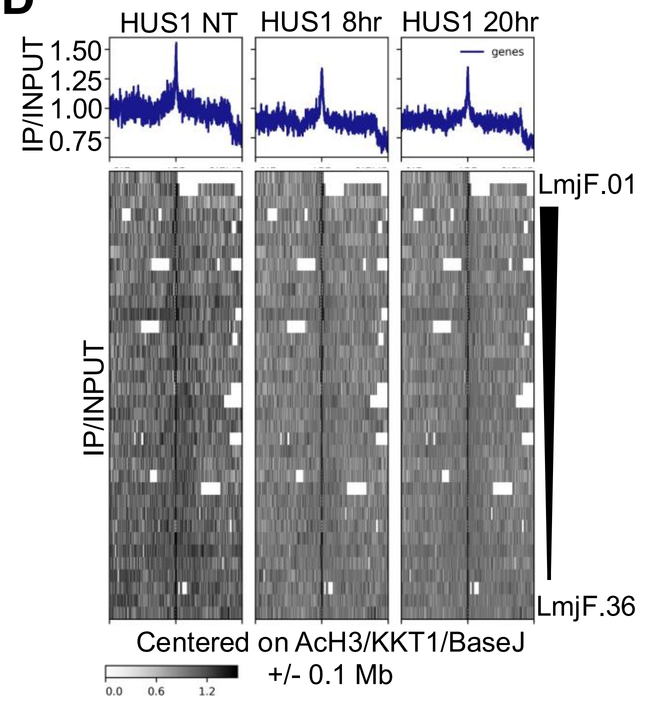

E

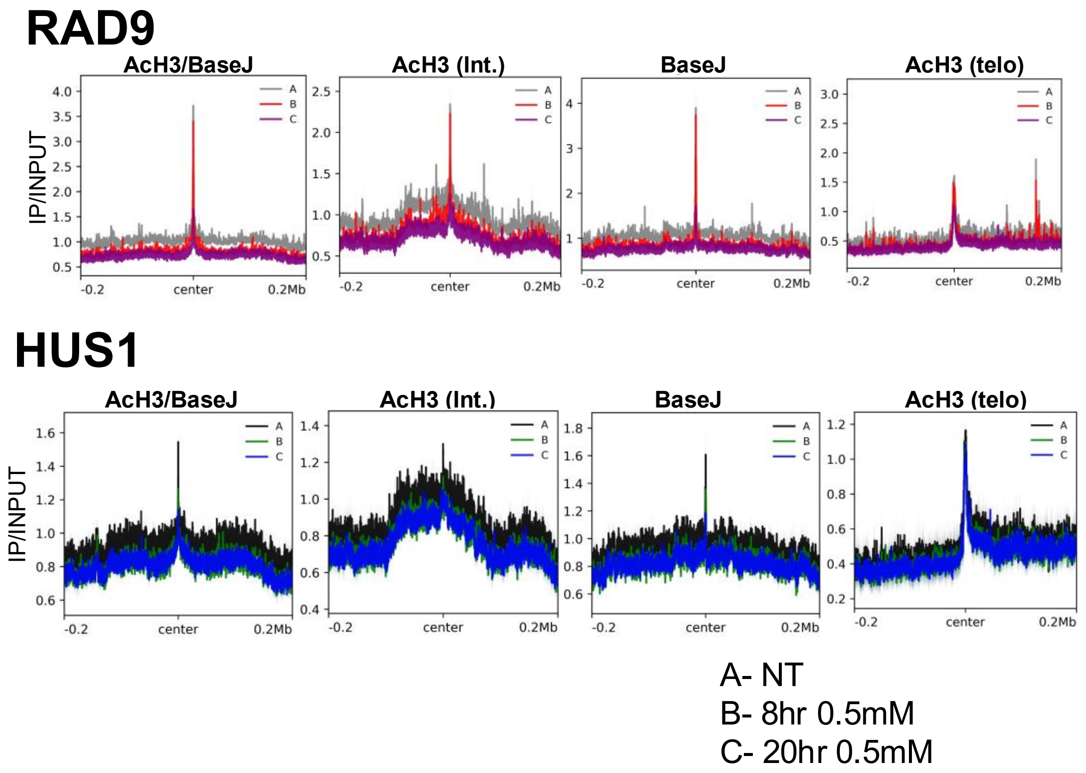

F

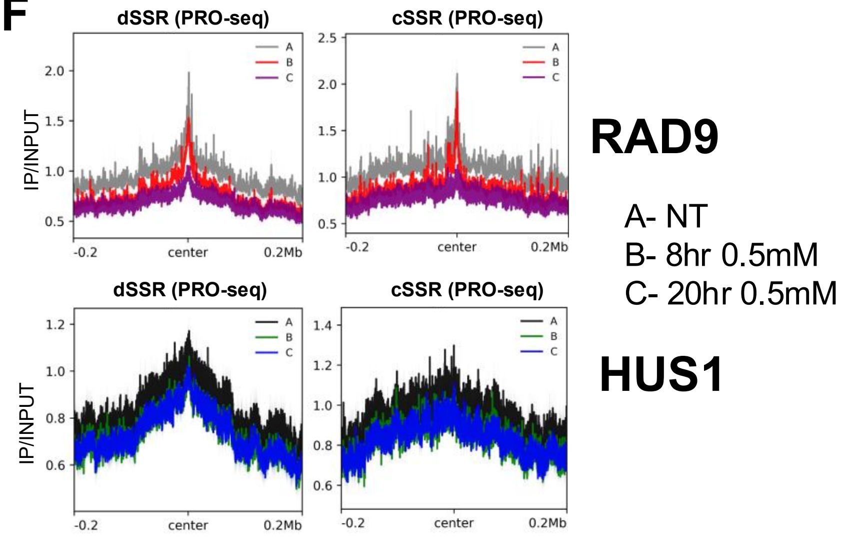

Data relating to Figure 5
Legend

(A) IGV plot showing normalised ChIP-seq signal for RAD9 and HUS1 under chronic RS. IP samples were normalized to their corresponding INPUT control samples. Scale 0.6-5.0 for HUS1 and RAD9, 0-4 AcH3, 0-400 BaseJ and 2-4 KKT1. LmjF.28 is shown as a representative chromosome.
(B) Spearman coefficient correlation analysis to assess the global comparison of RAD9, HUS1, RPA1 in ATR-competent cells and ATRΔC+/- cells ChIP-seq signals
(C) Heatmaps show the enriched signals for RAD9 and HUS1 centered on the early origin site +/- 0.1Mb.
(D) Heatmaps show the enriched signals for HUS1 centered on the early origin site +/- 0.1Mb.
(E) Metaplot analysis showing the enriched signals for RAD9 and HUS1 centered on AcH3, AcH3/BaseJ, AcH3 (Telo) and BaseJ sites +/- 0.2Mb. ChIP-seq samples were normalised to their corresponding input controls
(F) Metaplot analysis showing the enriched signals for RAD9 and HUS1 centered on PRO-seq identified dSSRs and cSSRs +/- 10kb. ChIP-seq samples were normalised to their corresponding input controls
