# Supplementary material for: Genome-wide mapping of replication stress response factors reveals the management of polycistronic transcription, replication initiation, and responses to replication stress in Leishmania
Source: Nucleic Acids Res. 2026 Aug 10;54(15):gkag754. doi: 10.1093/nar/gkag754 (PMC13454844; doi:10.1093/nar/gkag754)
Supplement: Supplementary file 1 [file gkag754_supplemental_files.zip › Supplementary_Data3.pdf]

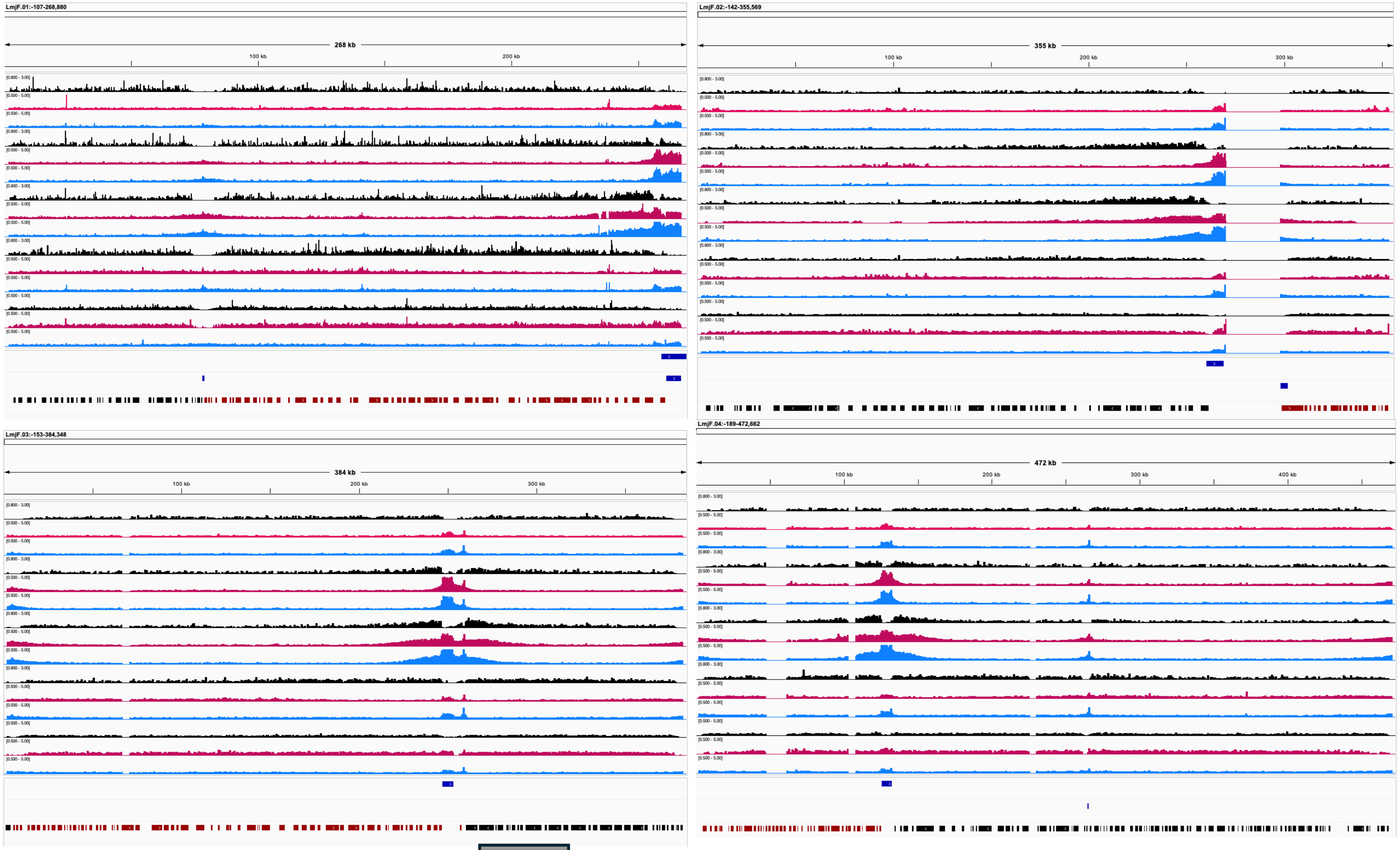

Order of Features (top-bottom):
Ac/KKT1/BaseJ
SSRs
CDSs

γH2A
RPA1
ATRΔ+/-

γH2A scale = [0.9-3.0]

RPA1+ATRΔ+/- scale = [0.5-5.0]

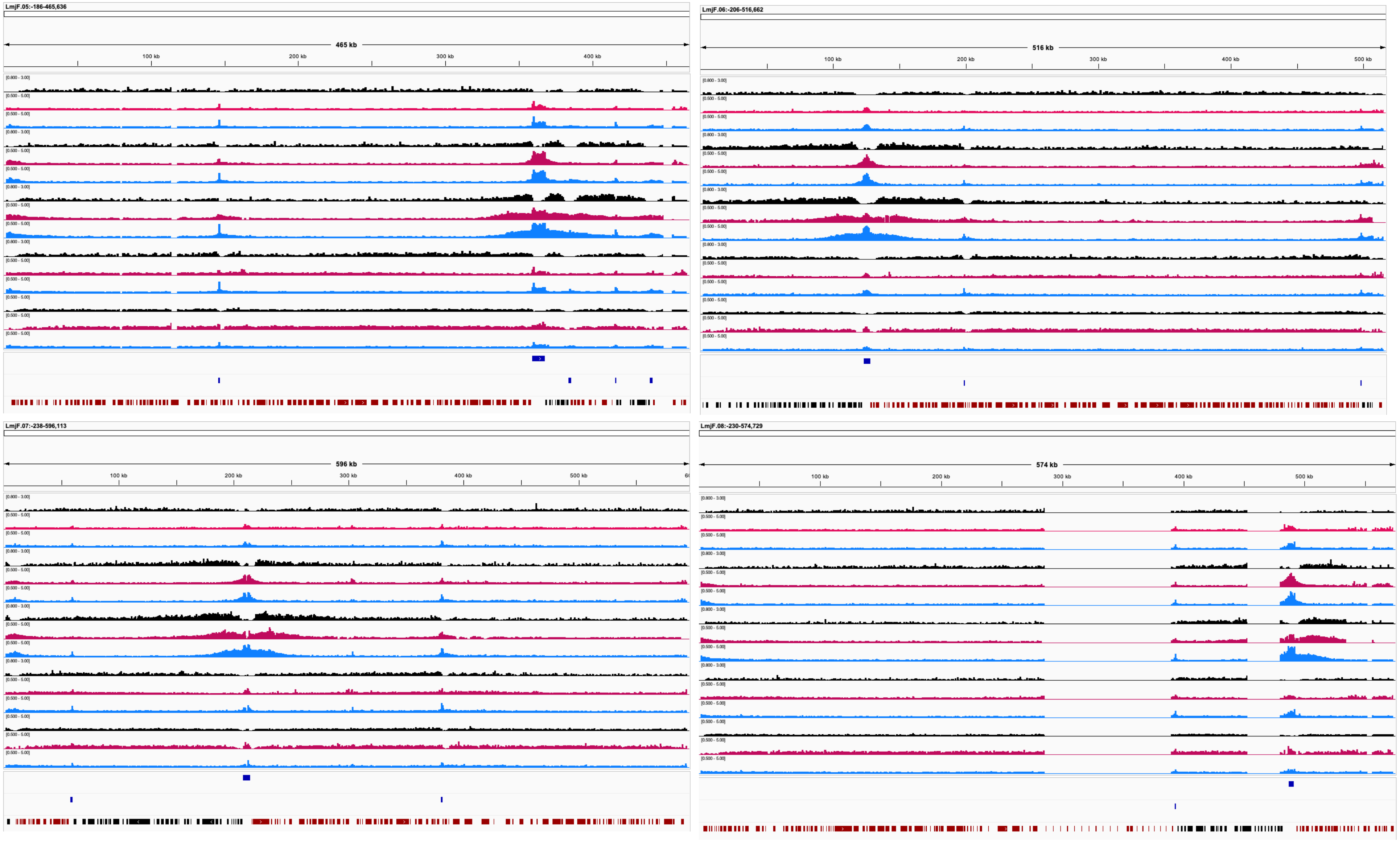

LmjF.05:-186-465,636
465 kb
100 kb
200 kb
300 kb
400 kb
[0.800 - 3.00]
[0.500 - 5.00]
LmjF.06:-206-516,662
516 kb
100 kb
200 kb
300 kb
400 kb
500 kb
LmjF.07:-238-596,113
596 kb
100 kb
200 kb
300 kb
400 kb
500 kb
LmjF.08:-230-574,729
574 kb
100 kb
200 kb
300 kb
400 kb
500 kb

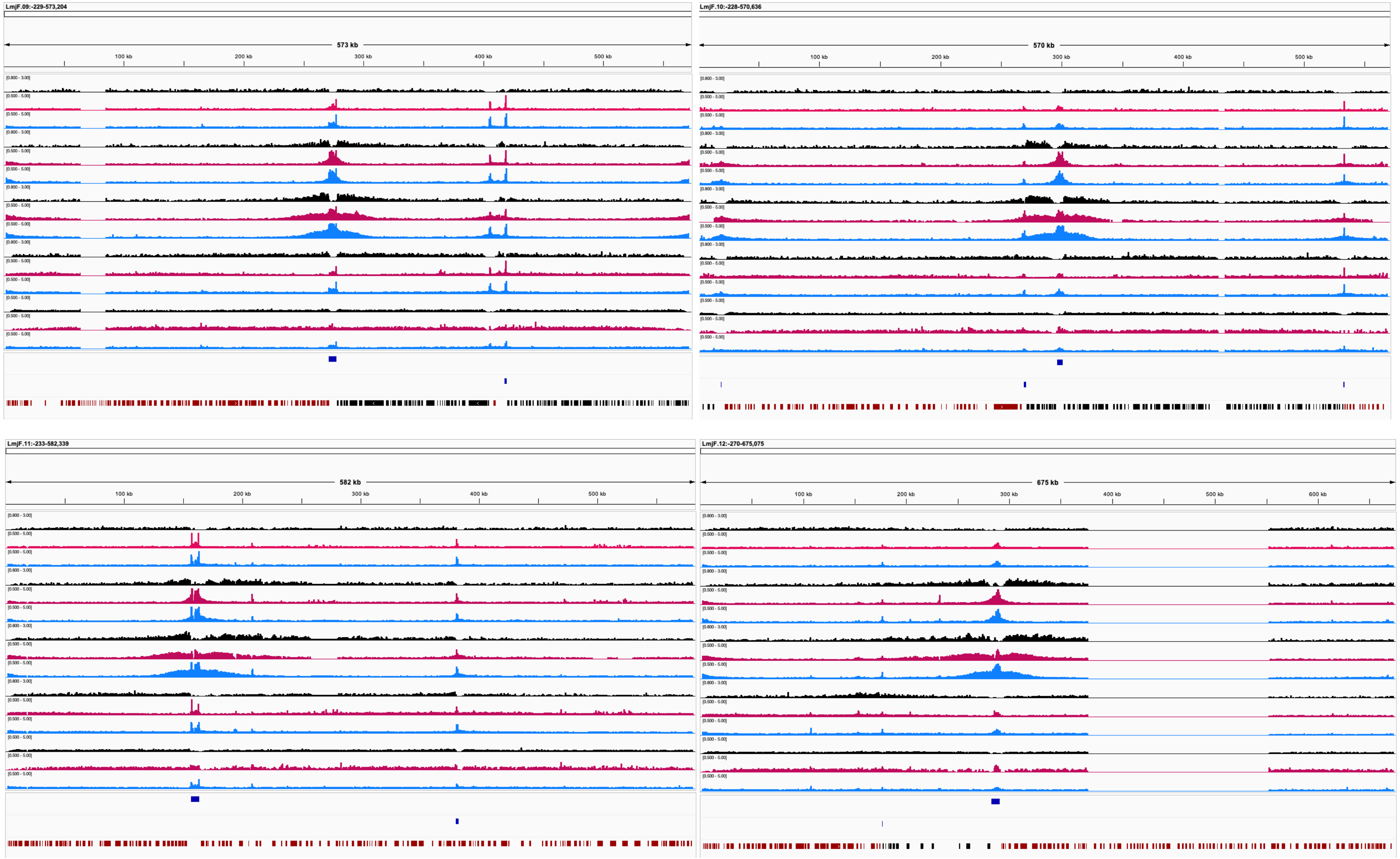

LmjF.09:-229-573,204
573 kb
100 kb
200 kb
300 kb
400 kb
500 kb
LmjF.10:-228-570,636
570 kb
100 kb
200 kb
300 kb
400 kb
500 kb
LmjF.11:-233-582,339
582 kb
100 kb
200 kb
300 kb
400 kb
500 kb
LmjF.12:-270-675,075
675 kb
100 kb
200 kb
300 kb
400 kb
500 kb
600 kb

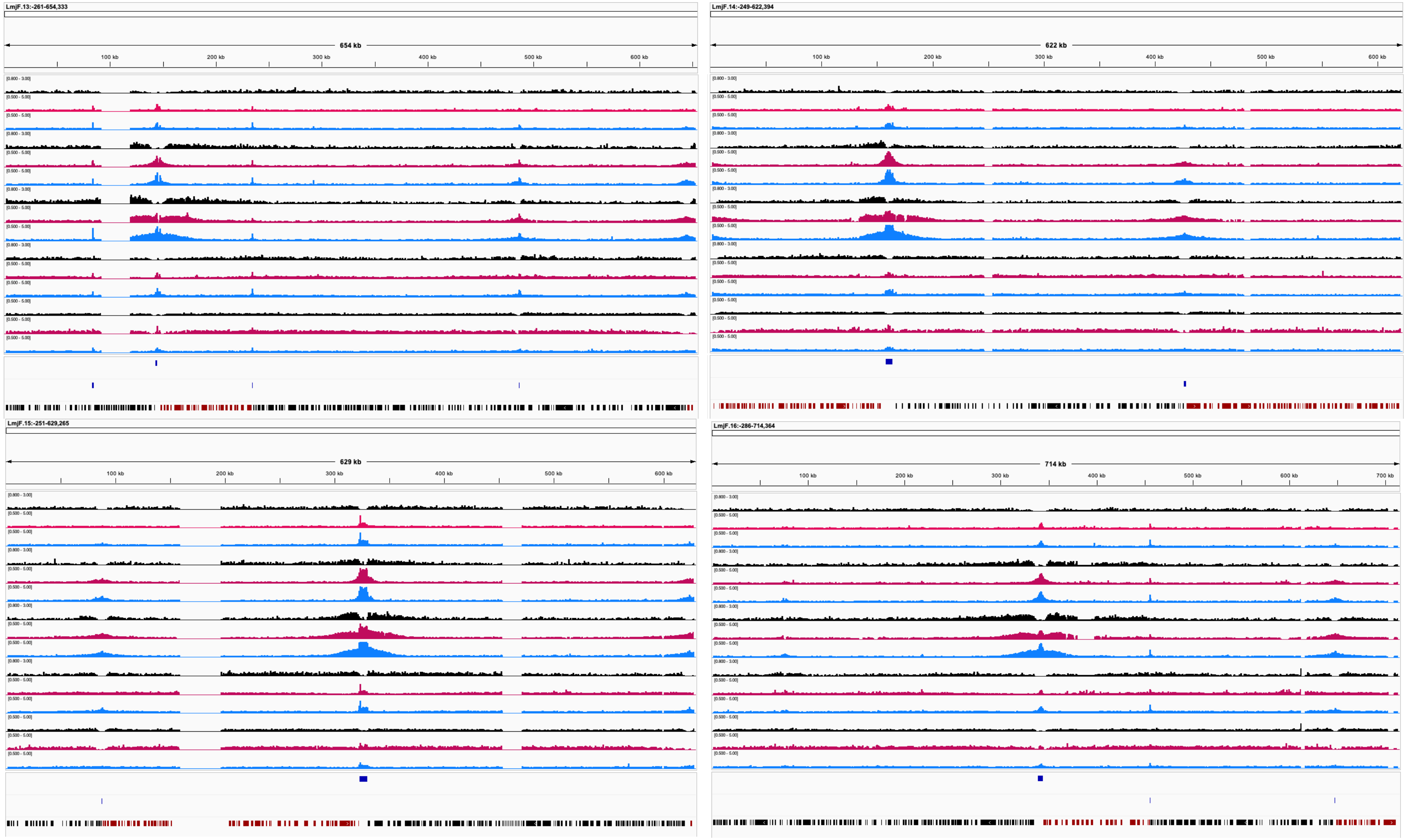

LmjF.13:-261-654,333
654 kb
100 kb
200 kb
300 kb
400 kb
500 kb
600 kb
LmjF.14:-249-622,394
622 kb
100 kb
200 kb
300 kb
400 kb
500 kb
600 kb
LmjF.15:-251-629,265
629 kb
100 kb
200 kb
300 kb
400 kb
500 kb
600 kb
LmjF.16:-286-714,364
714 kb
100 kb
200 kb
300 kb
400 kb
500 kb
600 kb
700 kb

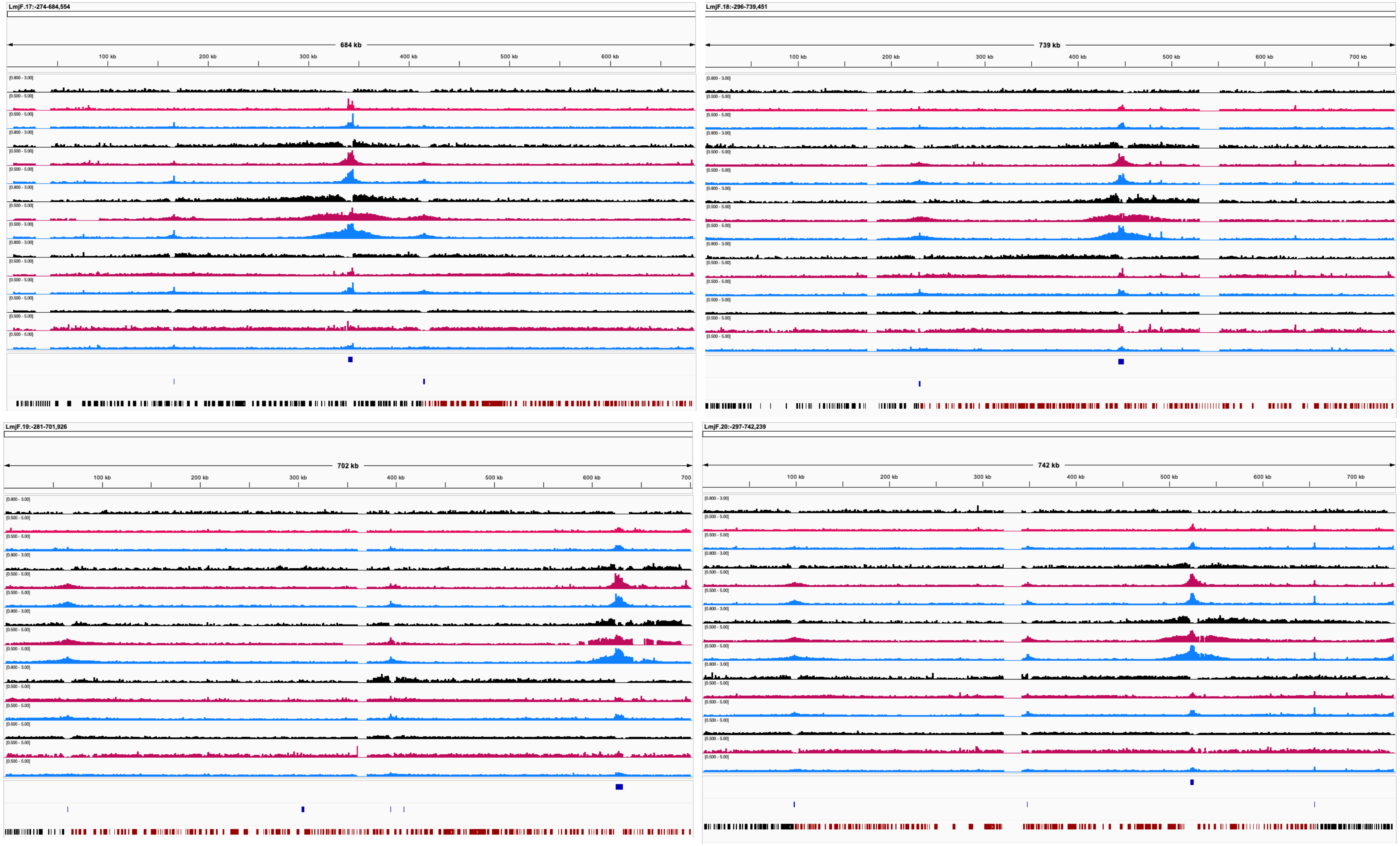
LmjF.17:-274-684,554
684 kb
100 kb
200 kb
300 kb
400 kb
500 kb
600 kb
LmjF.18:-296-739,451
739 kb
100 kb
200 kb
300 kb
400 kb
500 kb
600 kb
700 kb
LmjF.19:-281-701,926
702 kb
100 kb
200 kb
300 kb
400 kb
500 kb
600 kb
700
LmjF.20:-297-742,239
742 kb
100 kb
200 kb
300 kb
400 kb
500 kb
600 kb
700 kb

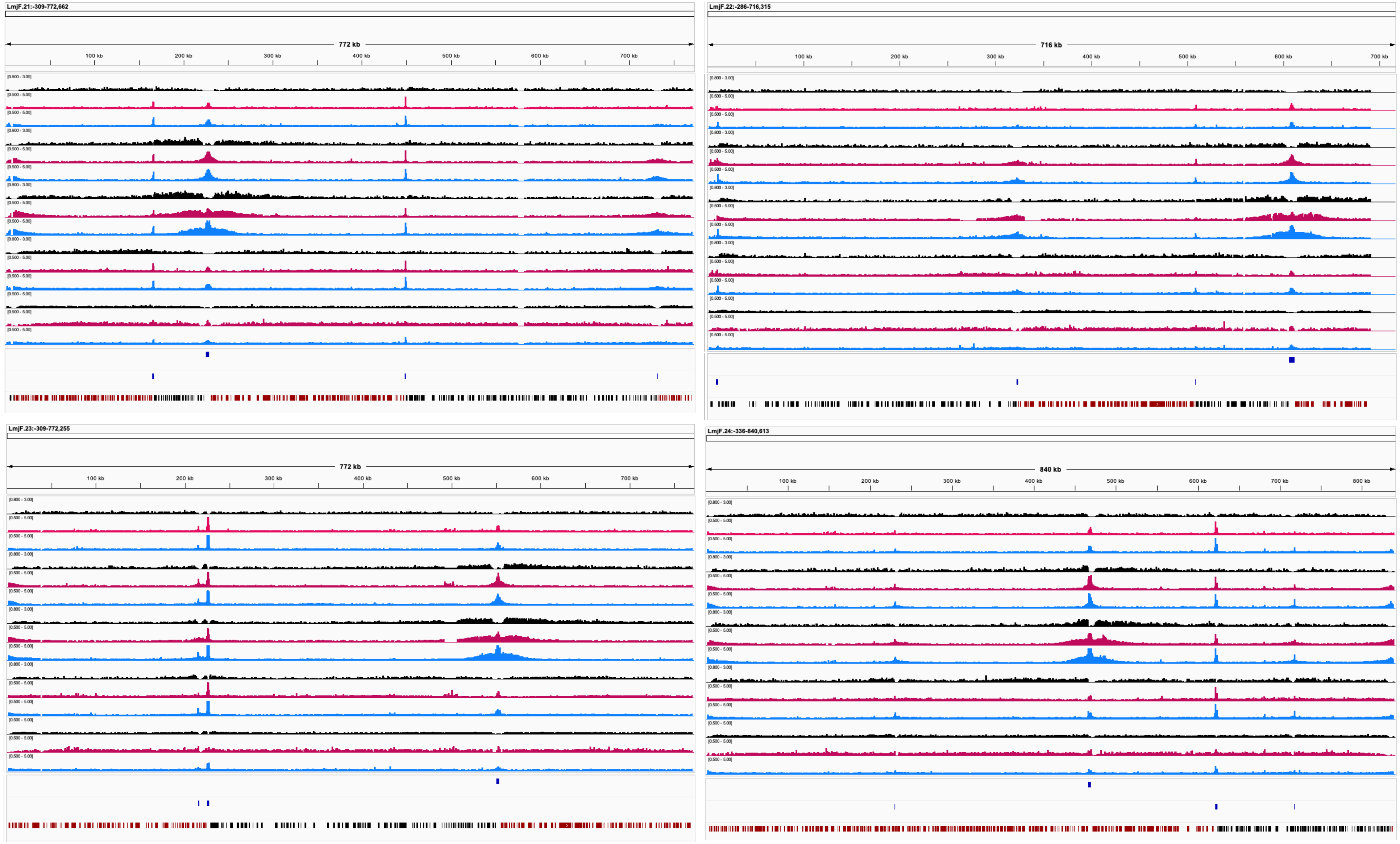
LmjF.21:-309-772,662
772 kb
100 kb
200 kb
300 kb
400 kb
500 kb
600 kb
700 kb
LmjF.22:-286-716,315
716 kb
100 kb
200 kb
300 kb
400 kb
500 kb
600 kb
700 kb
LmjF.23:-309-772,255
772 kb
100 kb
200 kb
300 kb
400 kb
500 kb
600 kb
700 kb
LmjF.24:-336-840,613
840 kb
100 kb
200 kb
300 kb
400 kb
500 kb
600 kb
700 kb
800 kb

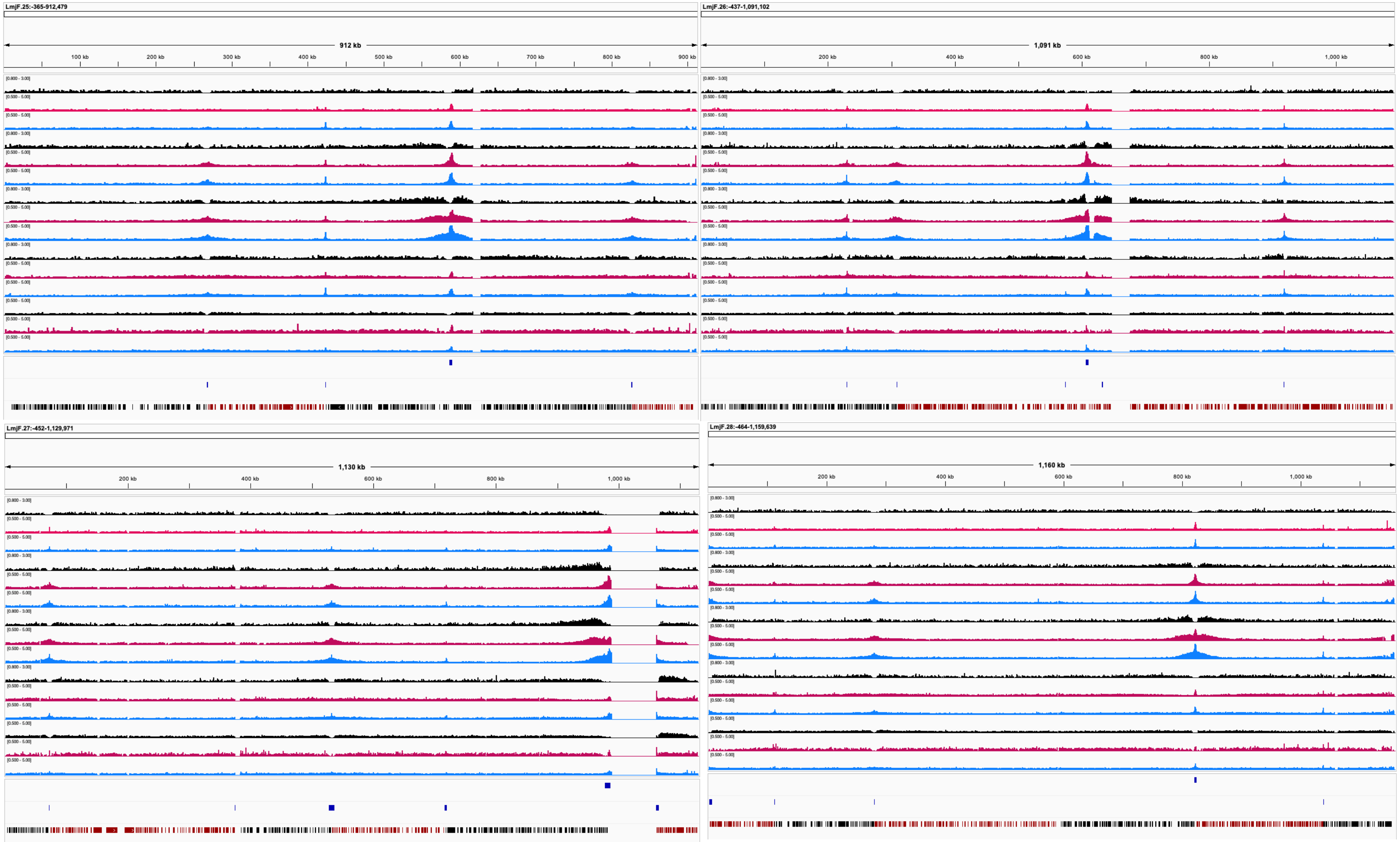
LmjF.25:-365-912,479
912 kb
100 kb
200 kb
300 kb
400 kb
500 kb
600 kb
700 kb
800 kb
900 kb
LmjF.26:-437-1,091,102
1,091 kb
200 kb
400 kb
600 kb
800 kb
1,000 kb
LmjF.27:-452-1,129,971
1,130 kb
200 kb
400 kb
600 kb
800 kb
1,000 kb
LmjF.28:-464-1,159,639
1,160 kb
200 kb
400 kb
600 kb
800 kb
1,000 kb

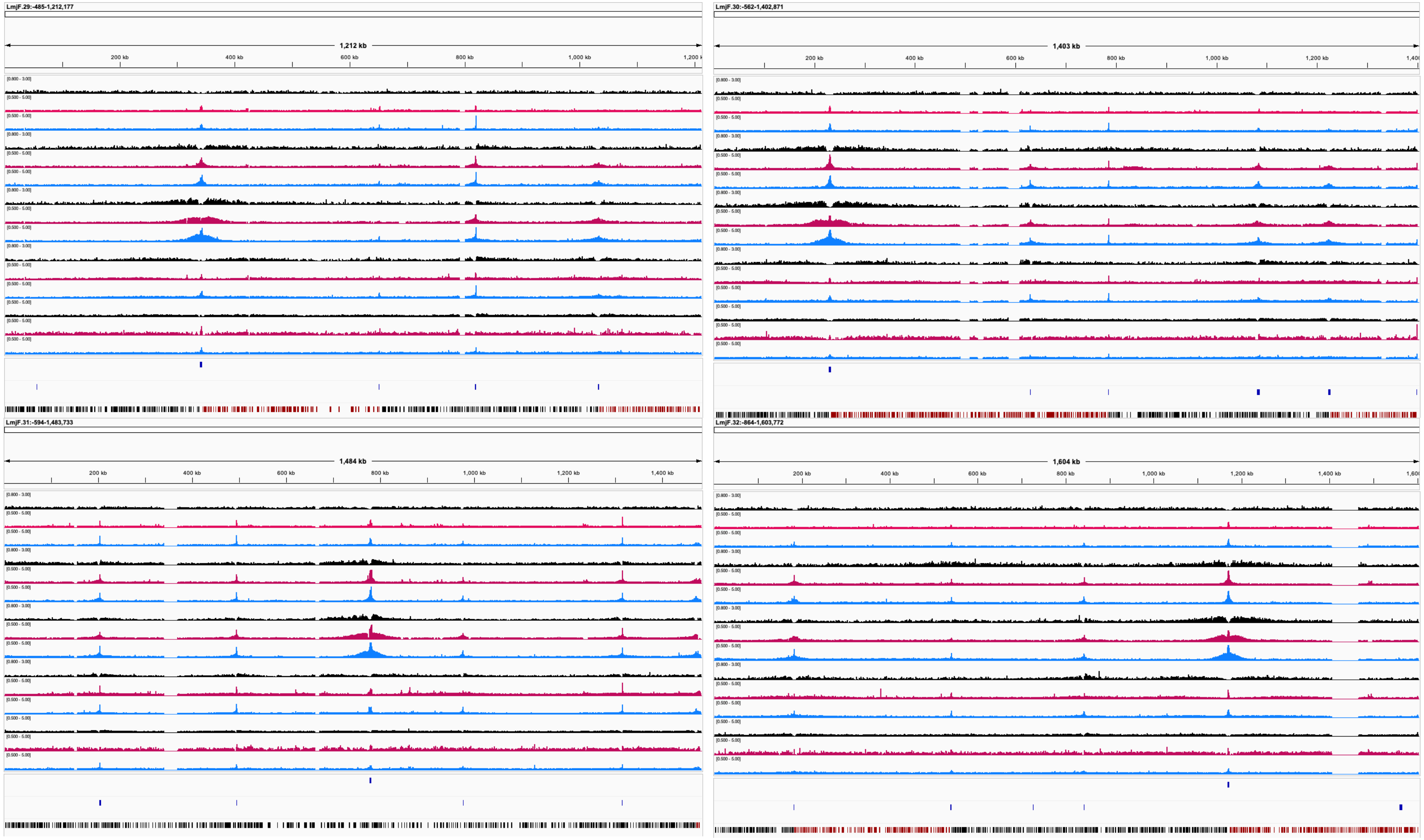
LmjF.29:-485-1,212,177
1,212 kb
200 kb
400 kb
600 kb
800 kb
1,000 kb
LmjF.30:-562-1,402,871
1,403 kb
LmjF.31:-594-1,483,733
1,484 kb
1,200 kb
1,400 kb
LmjF.32:-864-1,603,772
1,604 kb

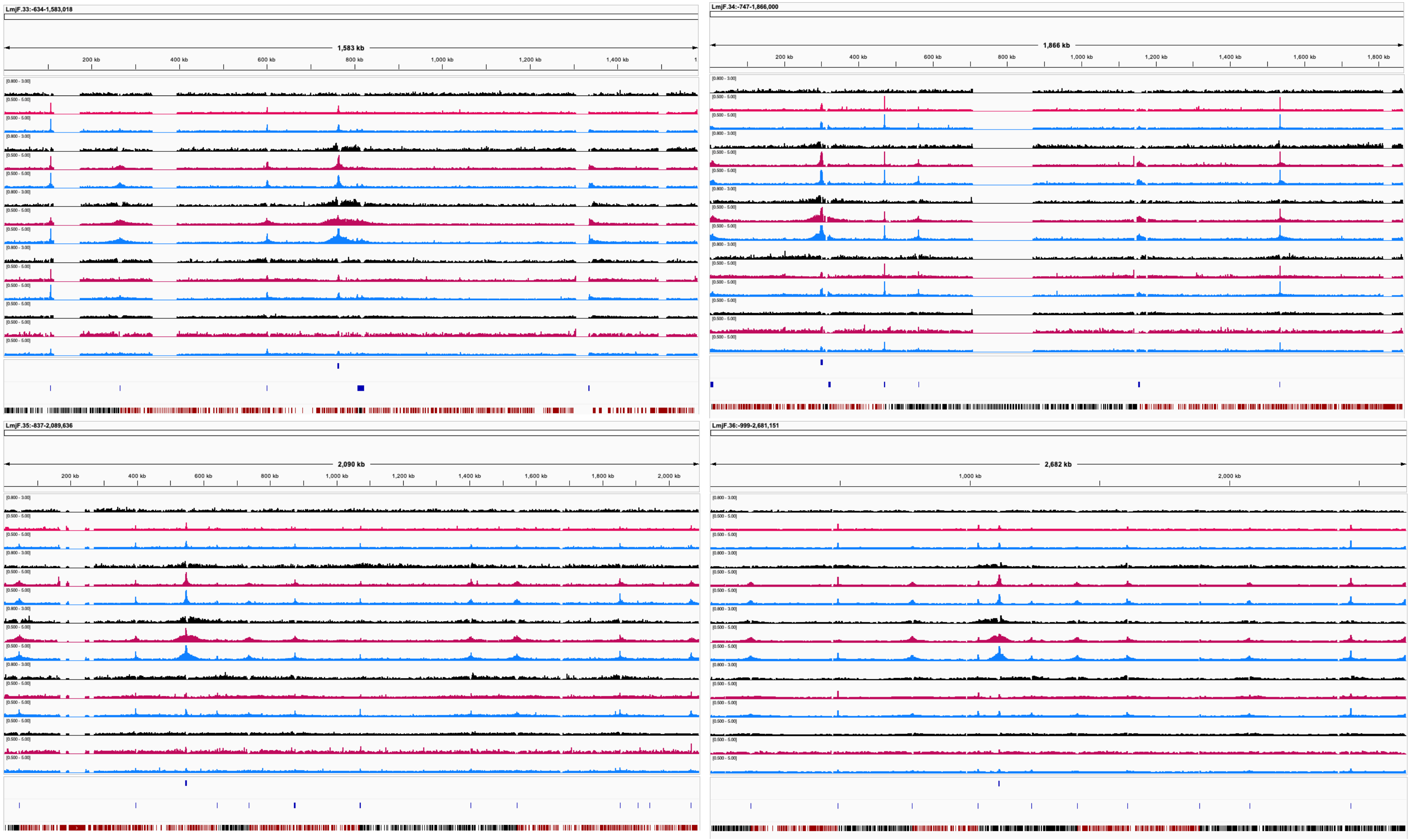
LmjF.33:-634-1,583,018
1,583 kb
LmjF.34:-747-1,866,000
1,866 kb
LmjF.35:-837-2,089,636
2,090 kb
LmjF.36:-999-2,681,151
2,682 kb
