# Supplementary material for: Genome-wide mapping of replication stress response factors reveals the management of polycistronic transcription, replication initiation, and responses to replication stress in Leishmania
Source: Nucleic Acids Res. 2026 Aug 10;54(15):gkag754. doi: 10.1093/nar/gkag754 (PMC13454844; doi:10.1093/nar/gkag754)
Supplement: Supplementary file 1 [file gkag754_supplemental_files.zip › Supplementary_Figures.pdf]

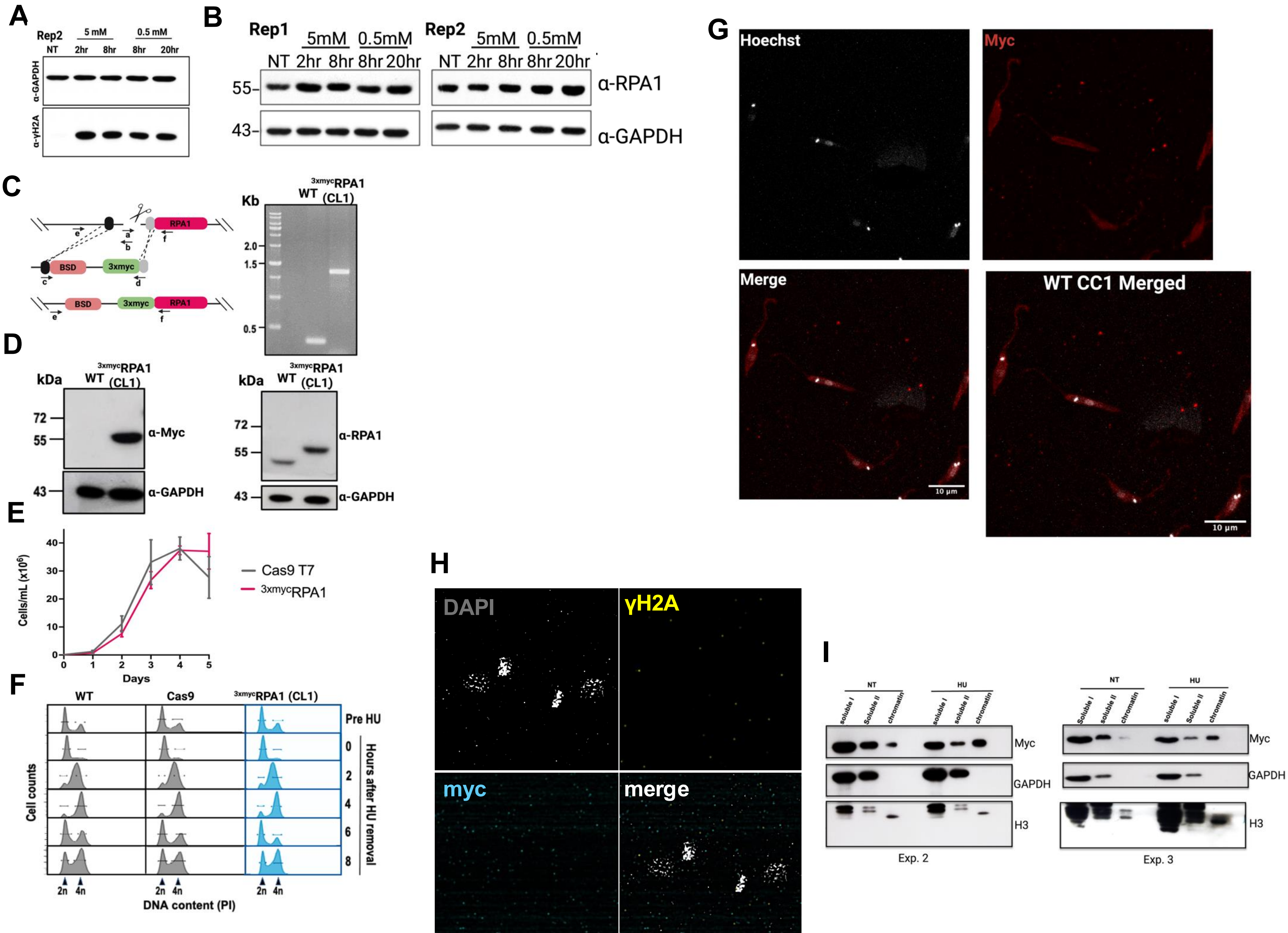
A
Rep2
5 mM
0.5 mM
NT
2hr
8hr
8hr
20hr
α-GAPDH
α-γH2A
B
Rep1
5mM
0.5mM
NT 2hr 8hr 8hr 20hr
Rep2
5mM
0.5mM
NT 2hr 8hr 8hr 20hr
55-
43-
α-RPA1
α-GAPDH
C
RPA1
BSD
3xmyc
Kb
WT
3xmycRPA1 (CL1)
2.0
1.5
0.5
D
kDa
WT
3xmycRPA1 (CL1)
72
55
43
α-Myc
α-GAPDH
α-RPA1
E
Cells/mL (x10^6)
Days
Cas9 T7
3xmycRPA1
F
WT
Cas9
3xmycRPA1 (CL1)
Pre HU
0
2
4
6
8
Hours after HU removal
Cell counts
2n
4n
DNA content (PI)
G
Hoechst
Myc
Merge
WT CC1 Merged
10 µm
H
DAPI
γH2A
myc
merge
5 µm
I
NT
HU
soluble I
soluble II
chromatin
Myc
GAPDH
H3
Exp. 2
Exp. 3

Figure S1

Figure S1. Endogenous tagging LmRPA1 does not affect cell cycle progression (A) Independent repeat 2 relating to Figure 1A showing yH2A signal after exposure to HU at two concentrations. (B) Independent repeats 2 and 3 showing RPA1 signal after exposure to HU at two concentrations over time. (C) Schematic representation of the method used to endogenously tag RPA1 with 3xmyc epitopes at the N-terminus of the protein using CRISPR/Cas9 genome engineering to generate mycRPA1 cells. (right) PCR confirmation of tag insertion into the endogenous locus. (D) Immunoblot analyses confirming tag expression are shown below using anti-myc (left) and anti-RPA1(right). (E) Growth of the endogenously tagged mycRPA1 cells were compared to Cas9 T7 expressing control cells. Growth was assessed every 24 hrs by quantifying cell density. Error bars = +/- SD, n = 3 independent biological experiments. (F) Cell cycle progression was blocked using 5.0 mM HU for 8 hrs and mycRPA1, WT CC1 and Cas9 T7 cells reseeded in HU-free medium. Cells were collected at regular intervals after HU release as shown; DNA content was examined by flow cytometry with 50,000 events collected for analysis. PI = propidium iodide, n = 3 independent replications/cell line. FACs data was analysed using FlowJo. (G) Representative field of view images from untagged control cells. DNA and kDNA were stained using Hoechst (grey), and anti-myc antiserum used to detect my signal (red). Images were taken on a Zeiss Multiphoton confocal microscope, scale bar = 10 µm. Enlarged and merged images are shown in H. Scale bar = = 10 µm. (H) Representative field of view images from untagged control cells. DNA and kDNA were stained using DAPI (grey), anti-myc antiserum used to detect myc signal (cyan) and Trypanosome anti- yH2A to detect yH2A (yellow). Images were taken on a Leica Stellaris STED microscope, scale bar = 5 µm. (I) Independent repeat 2 and 3 of immunoblots from the chromatin fractionation.

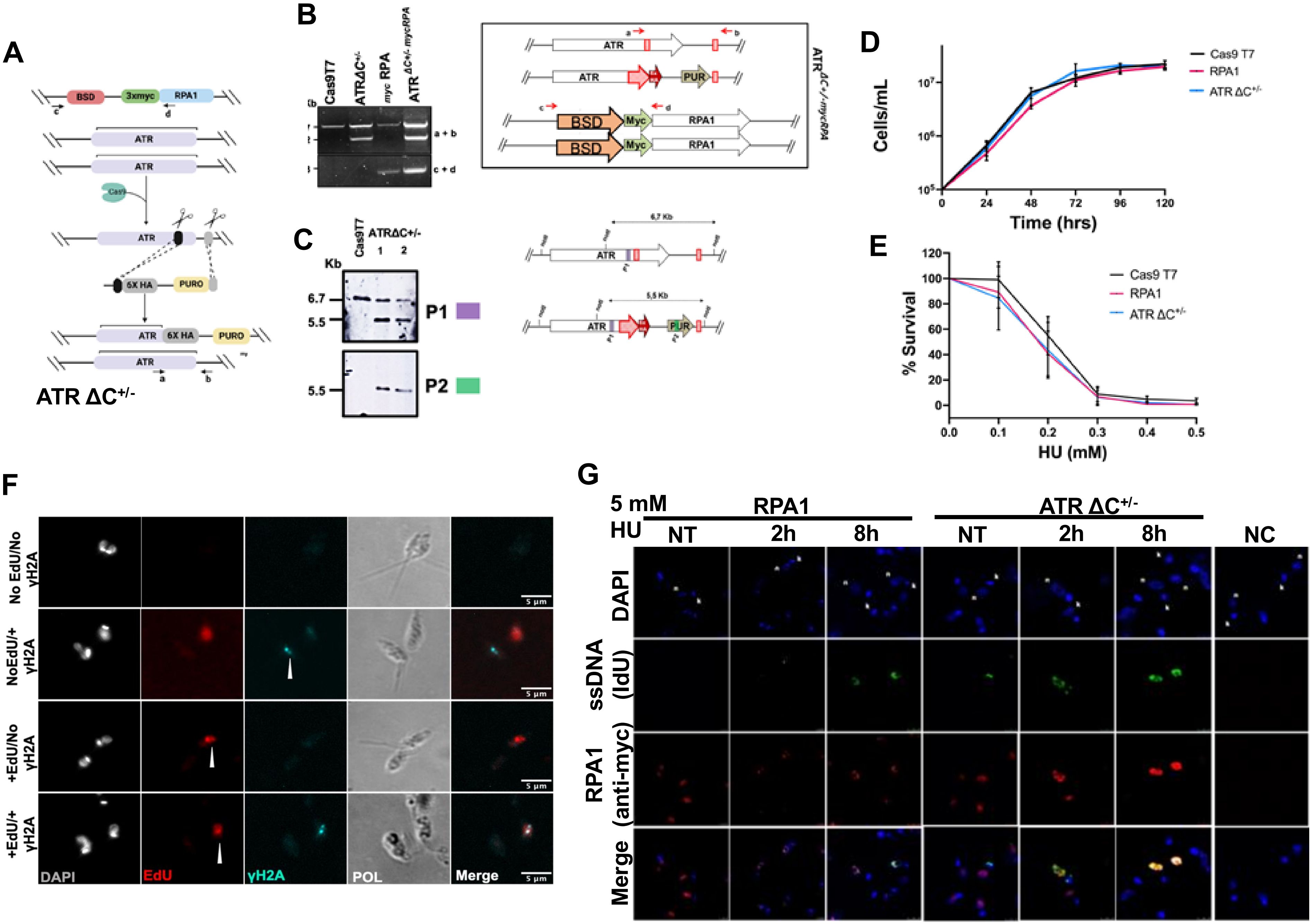
A
BSD
3xmyc
RPA1
ATR
Cas9
6X HA
PURO
ATR ΔC+/-
B
Cas9T7
ATRΔC+/-
myc RPA
ATR ΔC+/- mycRPA
a + b
c + d
ATR
PUR
BSD
Myc
C
Kb
6.7
5.5
P1
P2
6,7 Kb
5,5 Kb
D
Cells/mL
Time (hrs)
Cas9 T7
RPA1
ATR ΔC+/-
E
% Survival
HU (mM)
F
No EdU/No γH2A
NoEdU/+ γH2A
+EdU/No γH2A
+EdU/+ γH2A
DAPI
EdU
γH2A
POL
Merge
5 μm
G
5 mM HU
RPA1
ATR ΔC+/-
NT
2h
8h
NC
DAPI
ssDNA (IdU)
RPA1 (anti-myc)
Merge

Figure S2

Figure S2. ATR-deficiency enhances RS in *Leishmania* promastigotes (A) Schematic representation of the method used to truncate one allele of ATR in parasites expressing RPA1 fused to 3 myc epitopes at the N-terminus of the protein (mycRPA1) using CRISPR/Cas9 genome engineering. (B) PCR confirmation of the truncation of one allele of ATR and the schematic of primer locations used to confirm truncation (right). (C) Southern blotting reveals the cells possess one WT allele (6.7 kb) and one truncated allele (5.5 kb). Right shows schematic representing the Southern blotting strategy to confirm truncation of one allele of ATR. Location of probe 1 = grey square, location of probe 2 = green square. Cas9 T7 and ATRΔC+/- cells were digested with NotI for blotting. (D) Growth of the endogenously tagged mycRPA1 cells were compared to Cas9 T7 and ATRΔC+/- cells. Growth was quantified every 24 hrs by examining cell density. Error bars = +/- SD, n = 3 independent experiments. (E) Survival curve examining the percentage (%) survival of Cas9 T7, mycRPA1 and ATRΔC+/- cells after exposure to different concentrations of HU. Cells were counted 96 hrs after HU addition. Error bars = +/- SD, n = 3 independent experiments. (F) Representative images of the categories observed in Fig. 1G. EdU incorporation (red), yH2A (cyan) and DAPI (grey) are shown. Arrows indicate the applicable cell. Nuclear and kinetoplastid DNA is visualised by DAPI incorporation (grey). Images were captured on a Leica fluorescent microscope and processed in Fiji (ImageJ) and colours were enhanced for improved visualisation. Representative of n = 3 independent experiments. (G) IFA of ssDNA signal (green) and anti-myc signal (red; RPA1) in promastigotes in the absence and presence of HU treatment (5 mM; 2 hrs and 8 hrs) in control cells and cells expressing the truncation of one allele of ATR. Nuclear and kinetoplastid DNA is visualised by DAPI incorporation (blue). Negative control cells from the experiment are show in the last panel (NC, negative control).

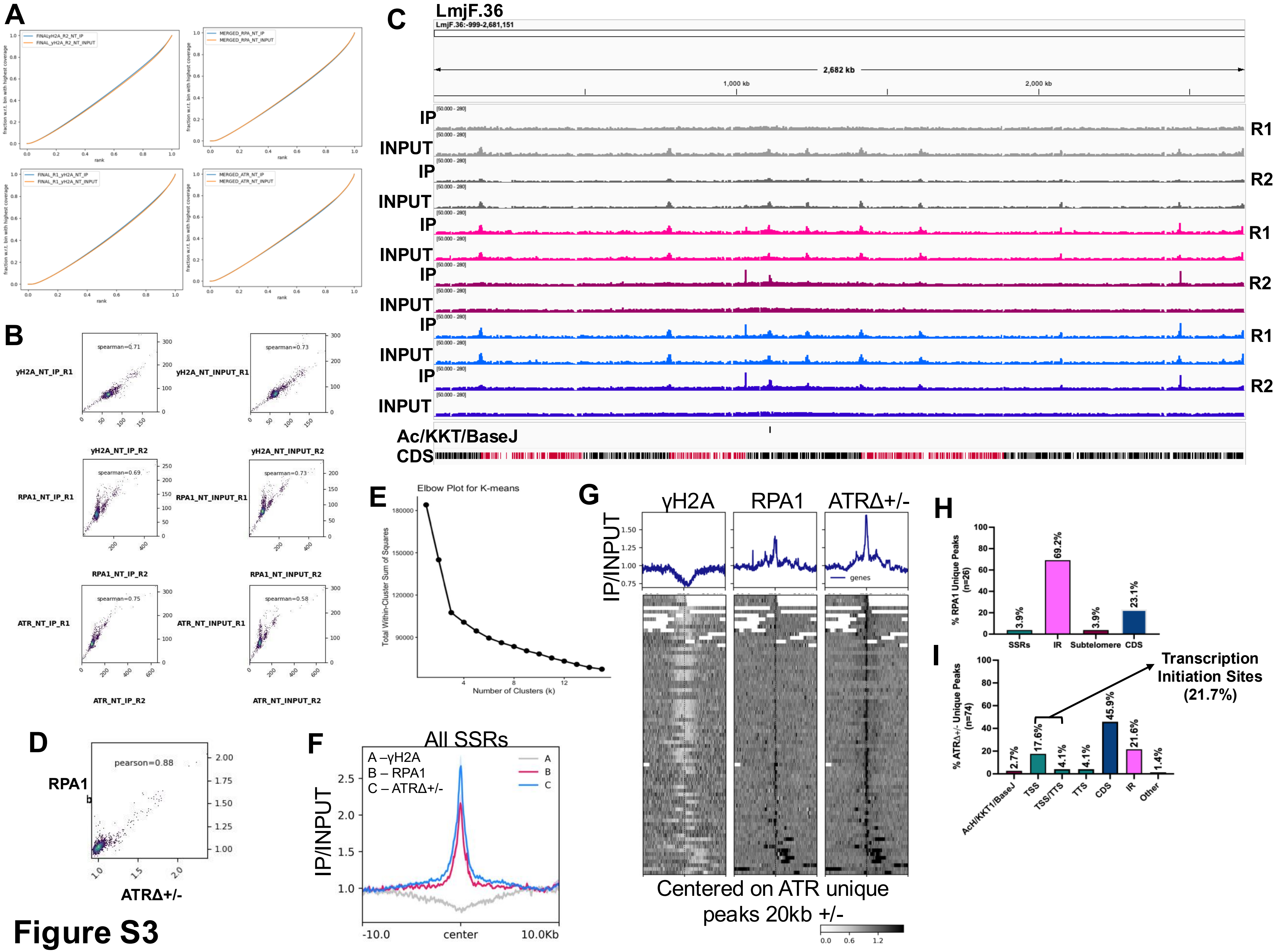

A
FINALyH2A_R2_NT_IP
FINAL_yH2A_R2_NT_INPUT
MERGED_RPA_NT_IP
MERGED_RPA_NT_INPUT
FINAL_R1_yH2A_NT_IP
FINAL_R1_yH2A_NT_INPUT
MERGED_ATR_NT_IP
MERGED_ATR_NT_INPUT
fraction w.r.t. bin with highest coverage
rank
B
spearman=0.71
spearman=0.73
yH2A_NT_IP_R1
yH2A_NT_INPUT_R1
yH2A_NT_IP_R2
yH2A_NT_INPUT_R2
spearman=0.69
spearman=0.73
RPA1_NT_IP_R1
RPA1_NT_INPUT_R1
RPA1_NT_IP_R2
RPA1_NT_INPUT_R2
spearman=0.75
spearman=0.58
ATR_NT_IP_R1
ATR_NT_INPUT_R1
ATR_NT_IP_R2
ATR_NT_INPUT_R2
C
LmjF.36
LmjF.36:-999-2,681,151
2,682 kb
1,000 kb
2,000 kb
IP
INPUT
R1
R2
Ac/KKT/BaseJ
CDS
D
pearson=0.88
RPA1
ATRΔ+/-
E
Elbow Plot for K-means
Total Within-Cluster Sum of Squares
Number of Clusters (k)
F
All SSRs
A –γH2A
B – RPA1
C – ATRΔ+/-
IP/INPUT
-10.0
center
10.0Kb
G
γH2A
RPA1
ATRΔ+/-
IP/INPUT
genes
Centered on ATR unique peaks 20kb +/-
H
% RPA1 Unique Peaks (n=26)
3.9%
69.2%
3.9%
23.1%
SSRs
IR
Subtelomere
CDS
I
% ATRΔ+/- Unique Peaks (n=74)
2.7%
17.6%
4.1%
4.1%
45.9%
21.6%
1.4%
AcH/KKT1/BaseJ
TSS
TSS/TTS
TTS
CDS
IR
Other
Transcription Initiation Sites (21.7%)

Figure S3

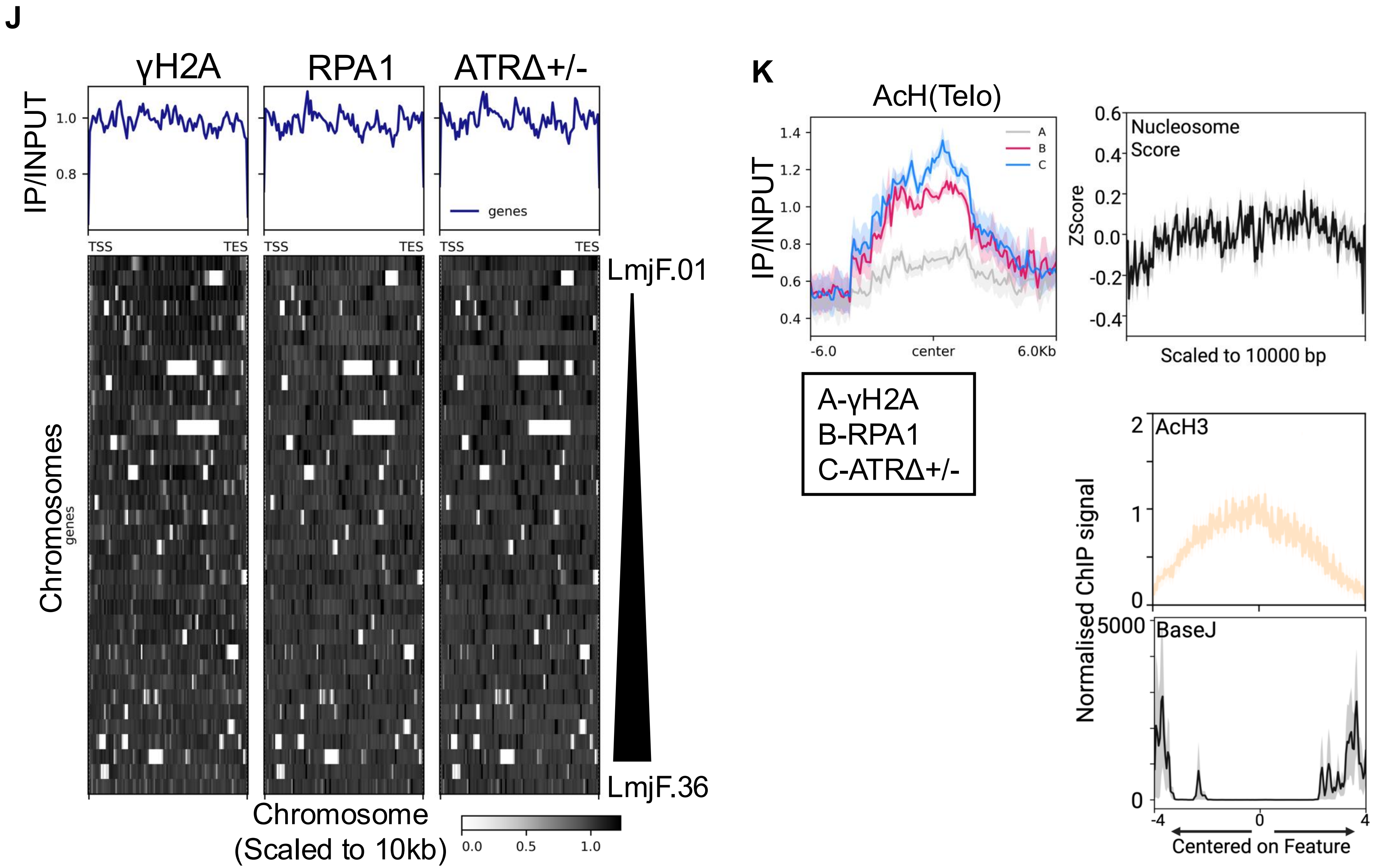

Figure S3 continued

Figure S3. Global distribution of yH2A and RPA1 in unperturbed cells and in unperturbed cells with one allele of ATR truncated (A) Fingerprint plots generated from bam files for each ChIP-seq dataset using DeepTools. (B) Spearman coefficient correlation analysis to assess the global comparison of ChIP-seq signal between biological duplicates for yH2A and RPA1 (in ATR-competent and ATRΔC+/- cells) revealed a strong correlation between replicates. (C) Normalised coverage files were prepared using bamCoverage (Deeptools) for yH2A and RPA1 signal from immunoprecipitated and input samples taken from unperturbed cells. Replicates (R1 & R2) are shown separately. ChIP-seq signals shown are mapped to a representative chromosome (LmjF.36). Samples are shown on a scale of 50-280. Tracks were viewed in IGV. Light and dark grey tracks are yH2A signal (R1 and R2 respectively), pink and red tracks are RPA1 signal in ATR-competent cells (R1 and R2 respectively), and blue and purple tracks are RPA1 in ATRΔC+/- cells (R1 and R2 respectively). CDSs are shown as black boxes (-ve strand transcription) or red boxes (+ve strand transcription). The AcH3/KKT1/BaseJ (early origin) is labelled by a blue box. (D) Pearson coefficient correlation analysis to assess the global comparison of RPA1 ChIP-seq signal between RPA1 signal in mycRPA1 and ATRΔC+/- cells in unperturbed cells uncovered strong correlations between ChIP-seq datasets. (E) Elbow plot used to determine K-means clustering relating to Figure 2C. (F) Metaplot analyses depicting enriched signal for yH2A, RPA1 and RPA1 in ATRΔC+/- cells centred on all non-origin associated SSRs +/- 10 kb. One representative experiment for yH2A is shown. For RPA1, signal reflects merged replicates. ChIP-seq samples were normalised to their corresponding input controls. Individual metaplots are shown for each protein. (G) Unique RPA1-bound sites in ATRΔC+/- cells identified using MACS2; the signals for yH2A, RPA1 and RPA1 (in ATRΔC+/-) were centered on these sites +/- 20 Kb up- and down-stream of the regions. The colour white on the heatmap indicates no signal. The genomic features associated with unique peaks of RPA1 (H) and RPA1 (in ATRΔC+/-) (I) shown as a percentage of the total number of common peaks. (J) Heatmap plot shows the signal for yH2A and RPA1 across the entirety of the chromosome from smallest (top) to largest (bottom) scaled to 10 Kb. (K) Metaplot analysis depicting enriched signal for yH2A and RPA1 in untreated cells around sites enriched for AcH3 within subtelomeric regions enriched for AcH3 (AcH3 Telo) +/- 6 kb. AcH3 (yellow) and BaseJ (black) signal are shown in the metaplots below. Nucleosome score is also provided as a normalised Z-score.

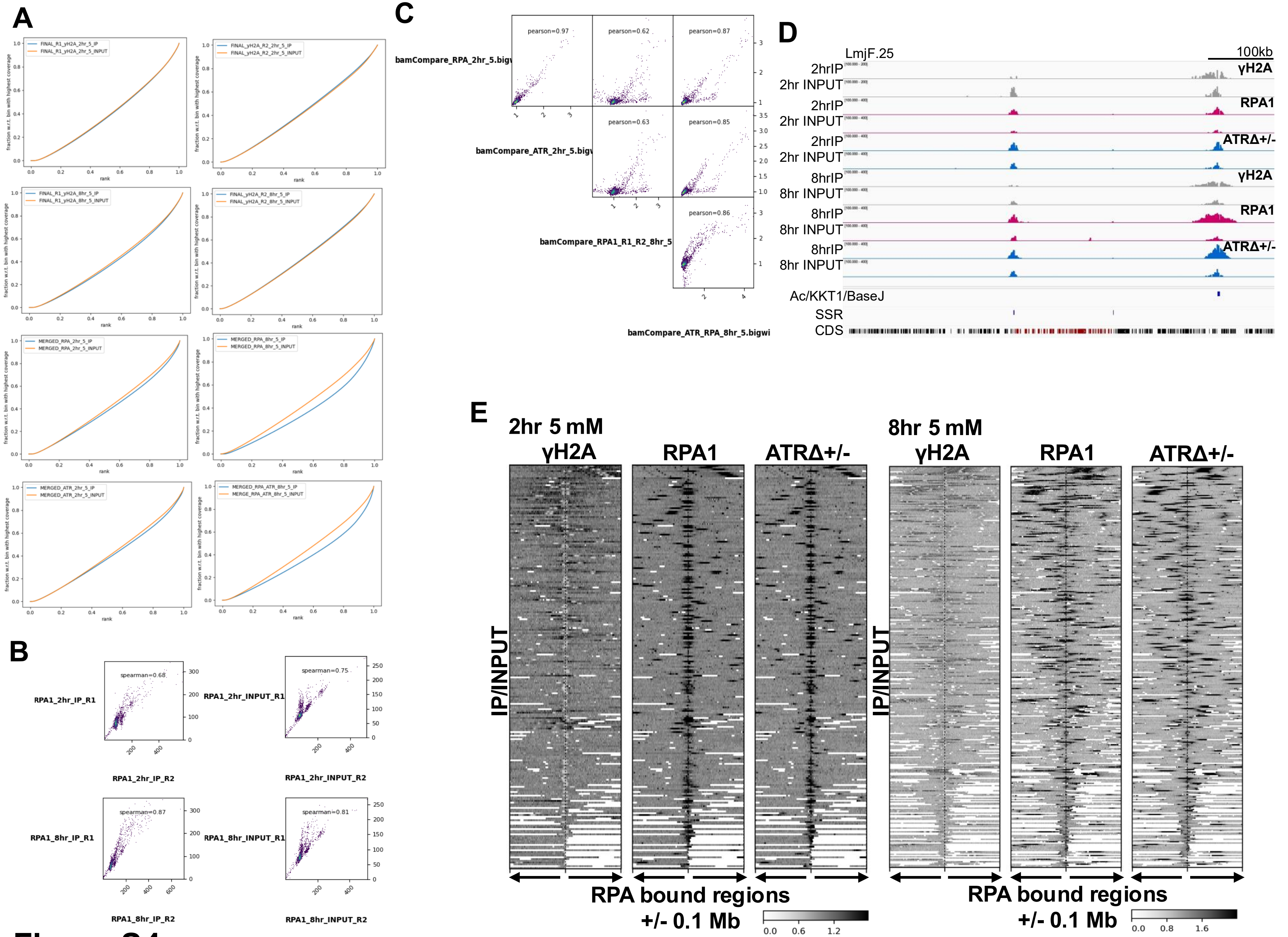
A
FINAL_R1_yH2A_2hr_5_IP
FINAL_R1_yH2A_2hr_5_INPUT
FINAL_yH2A_R2_2hr_5_IP
FINAL_yH2A_R2_2hr_5_INPUT
FINAL_R1_yH2A_8hr_5_IP
FINAL_R1_yH2A_8hr_5_INPUT
FINAL_yH2A_R2_8hr_5_IP
FINAL_yH2A_R2_8hr_5_INPUT
MERGED_RPA_2hr_5_IP
MERGED_RPA_2hr_5_INPUT
MERGED_RPA_8hr_5_IP
MERGED_RPA_8hr_5_INPUT
MERGED_ATR_2hr_5_IP
MERGED_ATR_2hr_5_INPUT
MERGED_RPA_ATR_8hr_5_IP
MERGE_RPA_ATR_8hr_5_INPUT
fraction w.r.t. bin with highest coverage
rank
B
spearman=0.68
RPA1_2hr_IP_R1
RPA1_2hr_IP_R2
spearman=0.75
RPA1_2hr_INPUT_R1
RPA1_2hr_INPUT_R2
spearman=0.87
RPA1_8hr_IP_R1
RPA1_8hr_IP_R2
spearman=0.81
RPA1_8hr_INPUT_R1
RPA1_8hr_INPUT_R2
C
pearson=0.97
pearson=0.62
pearson=0.87
pearson=0.63
pearson=0.85
pearson=0.86
bamCompare_RPA_2hr_5.bigw
bamCompare_ATR_2hr_5.bigw
bamCompare_RPA1_R1_R2_8hr_5
bamCompare_ATR_RPA_8hr_5.bigwi
D
LmjF.25
100kb
2hrIP
2hr INPUT
γH2A
RPA1
ATRΔ+/-
8hrIP
8hr INPUT
Ac/KKT1/BaseJ
SSR
CDS
E
2hr 5 mM
8hr 5 mM
γH2A
RPA1
ATRΔ+/-
IP/INPUT
RPA bound regions
+/- 0.1 Mb
0.0
0.6
1.2
0.8
1.6

Figure S4

Figure S4. Under acute RS, RSR factors accumulate around sites of DNA replication initiation (A) Fingerprint plots generated from bam files for each ChIP-seq dataset using DeepTools. (B) Spearman coefficient correlation analysis to assess the global comparison of ChIP-seq signal between biological duplicates for yH2A and RPA1 (in ATR-competent and ATRΔC+/- cells) revealed a strong correlation between replicates at 2 hrs and 8 hrs post exposure to 5 mM HU. (C) Pearson coefficient correlation analysis to assess the global comparison of RPA1 ChIP-seq signal between RPA1 signal in RPA1 and ATRΔC+/- cells. ChIP-seq signal at 2 hrs and 8 hrs uncovered strong correlations between RPA1 signal in ATR-competent and ATR-deficient cells. (D) Normalised coverage files were prepared using bamCoverage (Deeptools) for yH2A and RPA1 signal from immunoprecipitated and input samples taken from HU treated samples at 2hrs and 8hrs. Replicates (R1 & R2) are shown separately. ChIP-seq signals shown are mapped to a representative chromosome (LmjF.25). Samples are shown on a scale of 100-200 (yH2A) and 100-400 (RPA1). Tracks were viewed in IGV. CDSs are shown as black boxes (-ve strand transcription) or red boxes (+ve strand transcription). SSRs and the AcH3/KKT1/BaseJ (early origin) are labelled by blue boxes. (E) RPA1-bound sites identified using MACS2 were used to plot the signals for yH2A, RPA1 and RPA1 (in ATR-deficient cells) (left heatmap) and 8 hrs (right heatmap) post HU exposure. The enriched signals were centred on these sites and signal plotted +/- 0.1 Mb (2 hrs) and +/- 0.1 Mb (8 hrs) up- and down-stream of the regions. The colour white on the heatmap shows no binding.

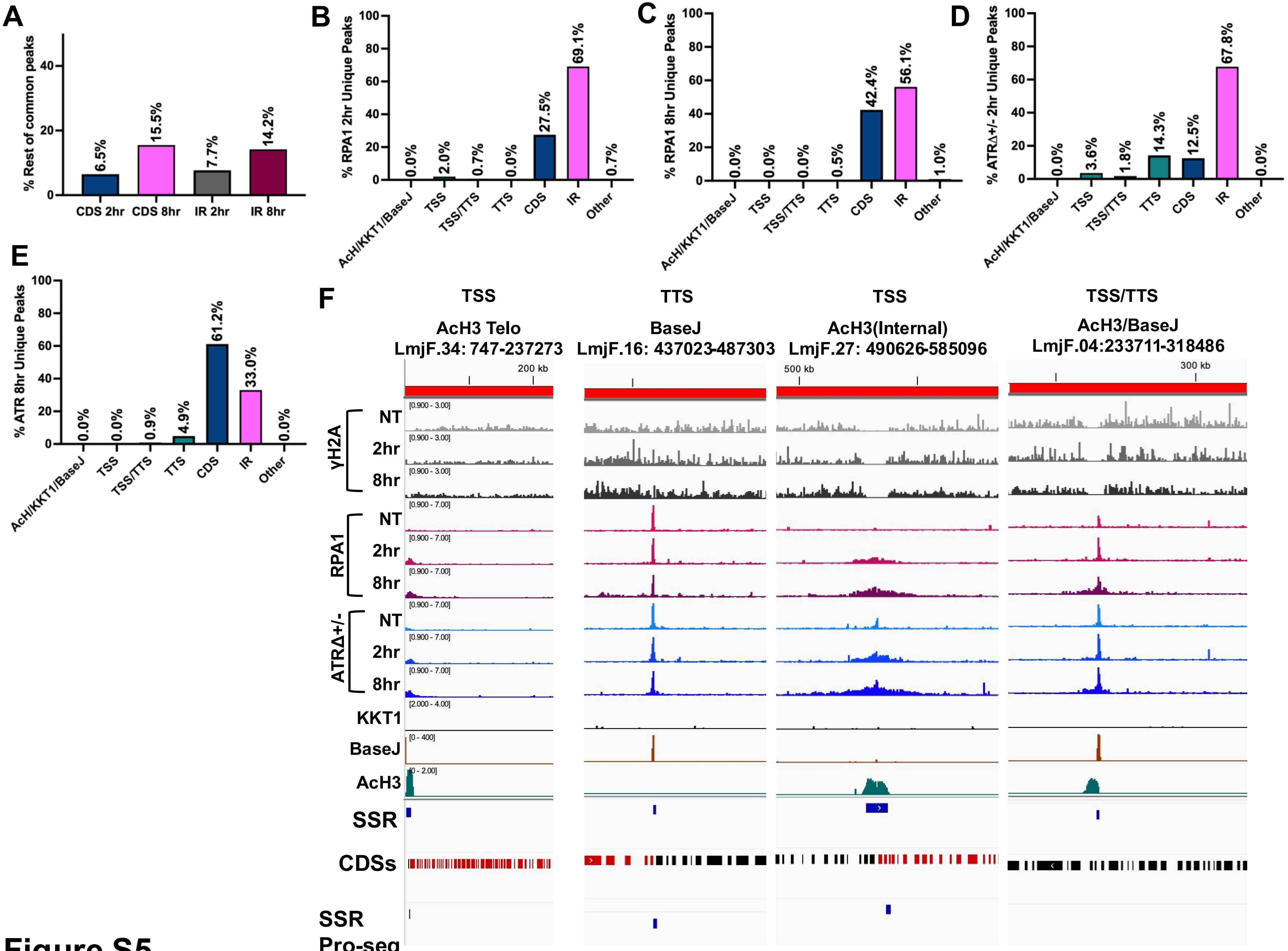
A
% Rest of common peaks
6.5%
15.5%
7.7%
14.2%
CDS 2hr
CDS 8hr
IR 2hr
IR 8hr
B
% RPA1 2hr Unique Peaks
0.0%
2.0%
0.7%
0.0%
27.5%
69.1%
0.7%
C
% RPA1 8hr Unique Peaks
0.0%
0.0%
0.0%
0.5%
42.4%
56.1%
1.0%
D
% ATRΔ+/- 2hr Unique Peaks
0.0%
3.6%
1.8%
14.3%
12.5%
67.8%
0.0%
AcH/KKT1/BaseJ
TSS
TSS/TTS
TTS
CDS
IR
Other
E
% ATR 8hr Unique Peaks
0.0%
0.0%
0.9%
4.9%
61.2%
33.0%
0.0%
F
TSS
AcH3 Telo
LmjF.34: 747-237273
TTS
BaseJ
LmjF.16: 437023-487303
TSS
AcH3(Internal)
LmjF.27: 490626-585096
TSS/TTS
AcH3/BaseJ
LmjF.04:233711-318486
γH2A
RPA1
ATRΔ+/-
NT
2hr
8hr
KKT1
BaseJ
AcH3
SSR
CDSs
SSR Pro-seq

Figure S5

Figure S5. Pattern of ChIP-seq signal enrichment at different SSR types in promastigote cells treated with 5 mM HU. (A) Genomic location of the rest of the common peaks of RPA1 and RPA1 in ATRΔC+/- in promastigote cells in the absence of HU and after 2 hrs and 8 hrs post HU exposure (5 mM). Unique RPA1 peaks associated with genomic features after 2hrs (B) and 8hrs (C) in control. Unique RPA1 peaks associated with genomic features after 2hrs (D) and 8hrs (E) in ATRΔC+/- cells. (F) ChIP-seq signals shown are mapped to representative SSRs on various chromosomes as detailed in the figure. ChIP-seq samples were normalised to input. Scale = 0.9 – 3.0 (yH2A) and 0.9 – 7.0 (RPA1. Tracks were viewed in IGV. Polycistronic unit negative (black), Polycistronic unit positive (red), SSR (blue box). Signal shown reflects repeats merged using mergeBam for RPA and a single representative repeat for yH2A.

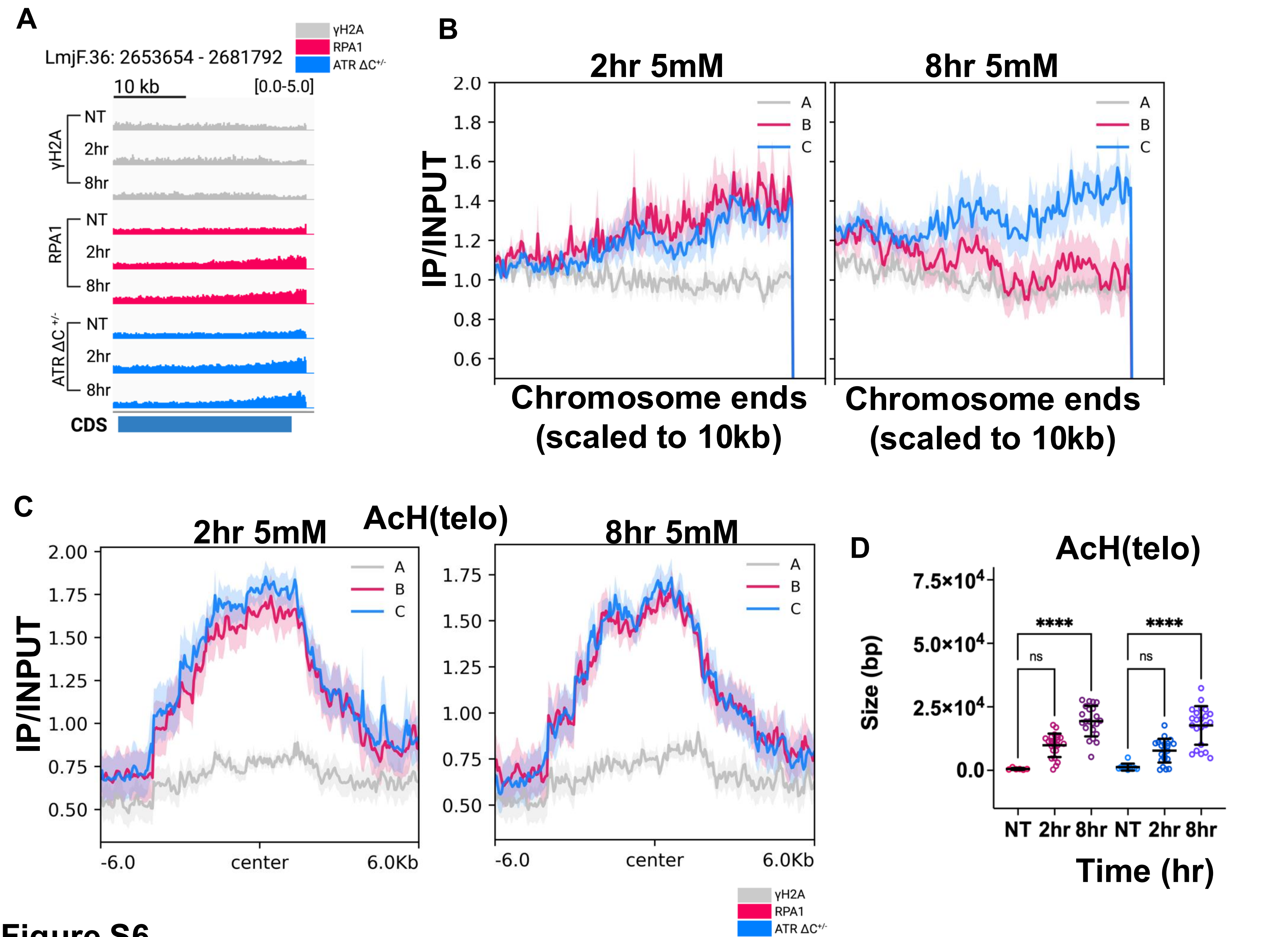

A
LmjF.36: 2653654 - 2681792
γH2A
RPA1
ATR ΔC+/-
10 kb
[0.0-5.0]
γH2A
NT
2hr
8hr
RPA1
NT
2hr
8hr
ATR ΔC +/-
NT
2hr
8hr
CDS
B
2hr 5mM
8hr 5mM
IP/INPUT
A
B
C
Chromosome ends (scaled to 10kb)
Chromosome ends (scaled to 10kb)
C
2hr 5mM
AcH(telo)
8hr 5mM
IP/INPUT
-6.0
center
6.0Kb
γH2A
RPA1
ATR ΔC+/-
D
AcH(telo)
Size (bp)
7.5×10^4
5.0×10^4
2.5×10^4
0.0
****
ns
NT 2hr 8hr NT 2hr 8hr
Time (hr)

Figure S6

Figure S6. Replication stress associated factors accumulate at subtelomeric sites in cells treated with HU (A) Location of yH2A and RPA1 in control cells and the location of RPA1 in ATRΔC+/- cells. ChIP-seq signals shown are mapped to a representative subtelomeric region on chromosome LmjF. 36. ChIP-seq samples were normalised to their corresponding input controls and shown on a scale of 0.0 - 5.0. Tracks were viewed in IGV. Polycistronic unit negative (blue). Signal shown reflects repeats merged using mergeBam except for yH2A for which a representative replicate is shown. (B) Metaplot analyses depicting enriched signals for yH2A, RPA1 and RPA1 in ATRΔC+/- within subtelomeric sites. Signals were scaled to 10kb. ChIP-seq samples were normalised to their corresponding input controls. (C) Metaplot analysis depicting enriched signal for yH2A and RPA1 at 2 hrs and 8 hrs centred on sites enriched for AcH3 within subtelomeric regions enriched for AcH3 (AcH3 Telo) +/- 6 kb. (D) Graph shows the sizes of peaks associated with AcH3 Telo sites were quantified. Significance was calculated using a Kruskal-Wallis test followed by Dunn's multiple comparisons test, ns = not significant, **** = < 0.0001.

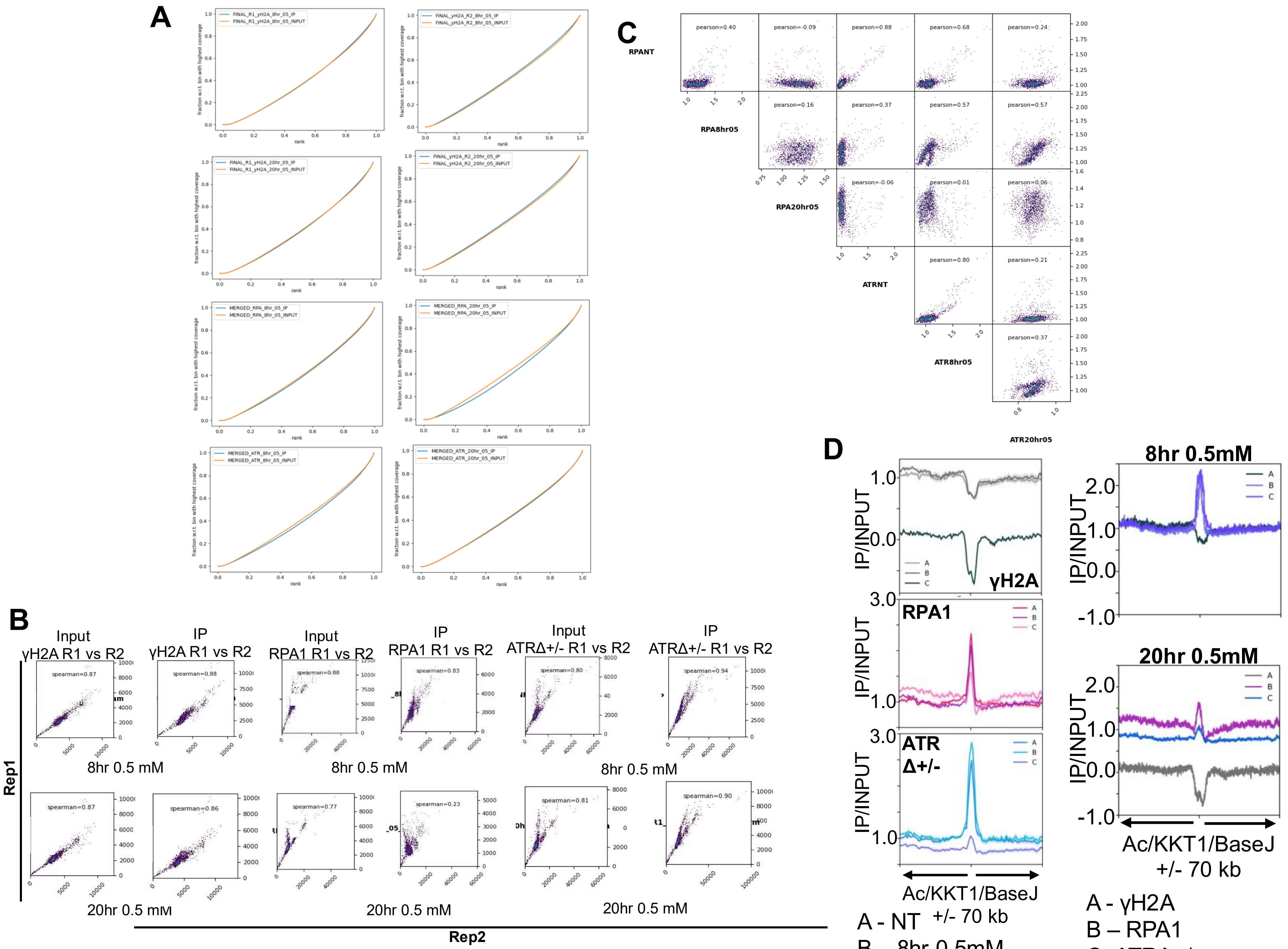
A
FINAL_R1_yH2A_8hr_05_IP
FINAL_R1_yH2A_8hr_05_INPUT
FINAL_yH2A_R2_8hr_05_IP
FINAL_yH2A_R2_8hr_05_INPUT
FINAL_R1_yH2A_20hr_05_IP
FINAL_R1_yH2A_20hr_05_INPUT
FINAL_yH2A_R2_20hr_05_IP
FINAL_yH2A_R2_20hr_05_INPUT
MERGED_RPA_8hr_05_IP
MERGED_RPA_8hr_05_INPUT
MERGED_RPA_20hr_05_IP
MERGED_RPA_20hr_05_INPUT
MERGED_ATR_8hr_05_IP
MERGED_ATR_8hr_05_INPUT
MERGED_ATR_20hr_05_IP
MERGED_ATR_20hr_05_INPUT
fraction w.r.t. bin with highest coverage
rank
B
Input γH2A R1 vs R2
IP γH2A R1 vs R2
Input RPA1 R1 vs R2
IP RPA1 R1 vs R2
Input ATRΔ+/- R1 vs R2
IP ATRΔ+/- R1 vs R2
spearman=0.87
spearman=0.88
spearman=0.88
spearman=0.83
spearman=0.80
spearman=0.94
8hr 0.5 mM
spearman=0.87
spearman=0.86
spearman=0.77
spearman=0.23
spearman=0.81
spearman=0.90
20hr 0.5 mM
Rep1
Rep2
C
RPANT
RPA8hr05
RPA20hr05
ATRNT
ATR8hr05
ATR20hr05
pearson=0.40
pearson=-0.09
pearson=0.88
pearson=0.68
pearson=0.24
pearson=0.16
pearson=0.37
pearson=0.57
pearson=0.57
pearson=-0.06
pearson=0.01
pearson=0.06
pearson=0.80
pearson=0.21
pearson=0.37
D
γH2A
RPA1
ATR Δ+/-
IP/INPUT
Ac/KKT1/BaseJ +/- 70 kb
A - NT
B – 8hr 0.5mM
C- 20hr 0.5mM
8hr 0.5mM
20hr 0.5mM
A - γH2A
B – RPA1
C- ATRΔ+/-

Figure S7

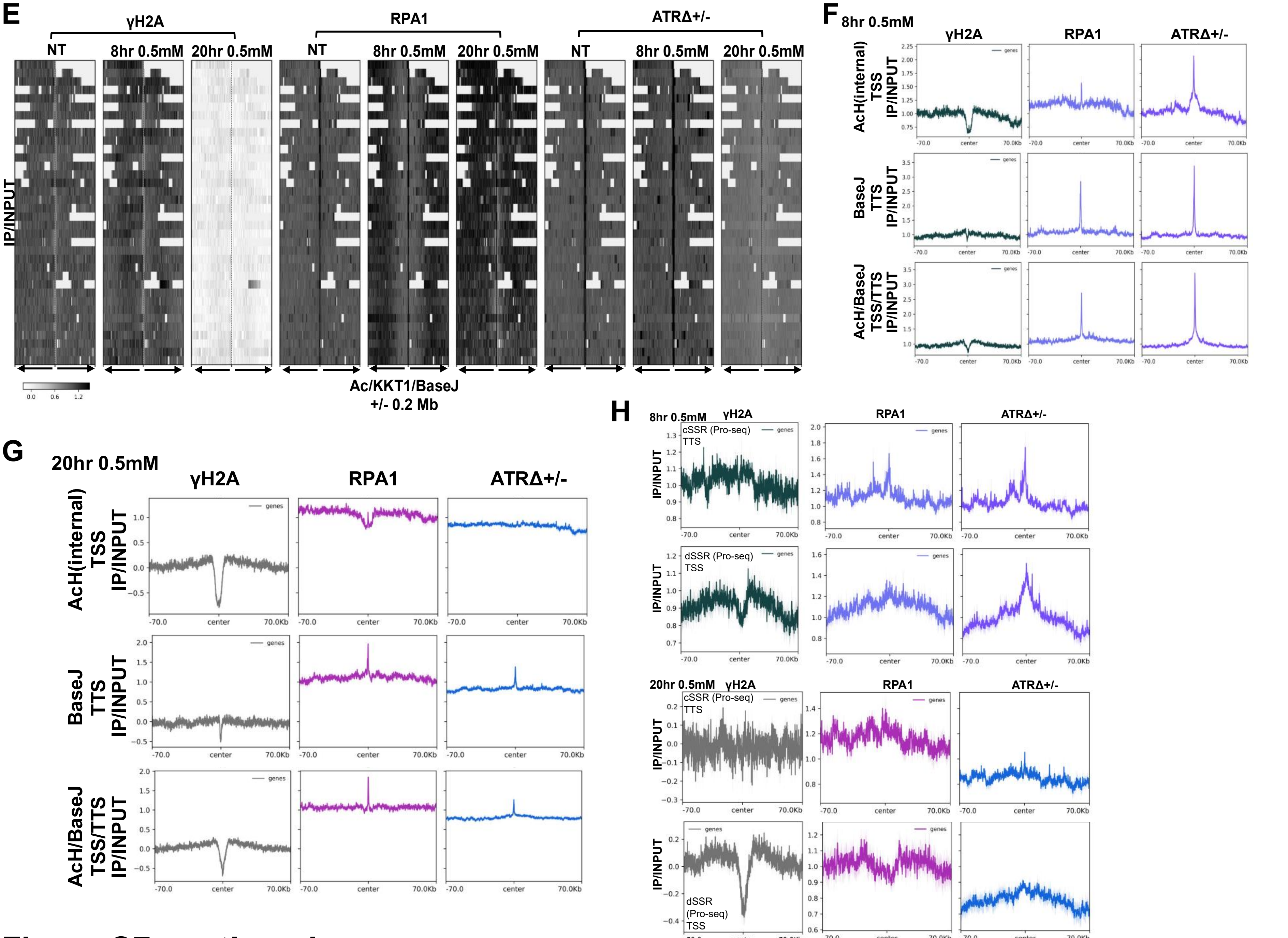

Figure S7 continued

Figure S7. Global distribution of yH2A and RPA1 signal following exposure to 0.5 mM HU (A) Fingerprint plots generated from bam files for each ChIP-seq dataset using DeepTools. (B) Spearman coefficient correlation analysis to assess the global comparison of yH2A, RPA1 ATR competent and ATRΔC+/- cells ChIP-seq signals uncovered a poor correlation between RPA1 signal in ATR-competent and ATR-deficient cells at both 8 and 20 hrs. (C) Pearson coefficient correlation analysis to assess the global comparison of RPA1 ChIP-seq signal between RPA1 signal in RPA1 and RPA1 in ATRΔC+/- cells at 8 hrs and 20 hrs, 0.5 mM HU exposure. (D) Metaplot analysis showing the enriched signals for yH2A, RPA1 (in control and ATRΔC+/- cells) centered on the early origin site (AcH3/KKT1/BaseJ) +/- 70,000 bp. ChIP-seq samples were normalised to their corresponding input controls. One representative experiment is shown for yH2A and merged replicates for RPA1. (E) Heatmaps show the enriched signals for yH2A and RPA1 (in control and ATR-deficient cells) centered on the early origin site +/- 0.2 Mb. Metaplot analysis showing the enriched signals for yH2A and RPA1 (in control and ATRΔC+/- cells) centered on AcH3, AcH3/BaseJ and BaseJ sites +/- 70kb. ChIP-seq samples were normalised to their corresponding input controls at 8hrs 0.5mM (F) and 20hrs 0.5mM (G). One representative experiment is shown for yH2A and merged replicates for RPA1. (H) Metaplot analysis showing the enriched signals for yH2A and RPA1 (in control and ATRΔC+/- cells) centered dSSR and cSSR sites as determined by PRO-seq nascent RNA sequencing +/- 70kb. ChIP-seq samples were normalised to their corresponding input controls at 8hrs 0.5mM (upper) and 20hrs 0.5mM (lower).

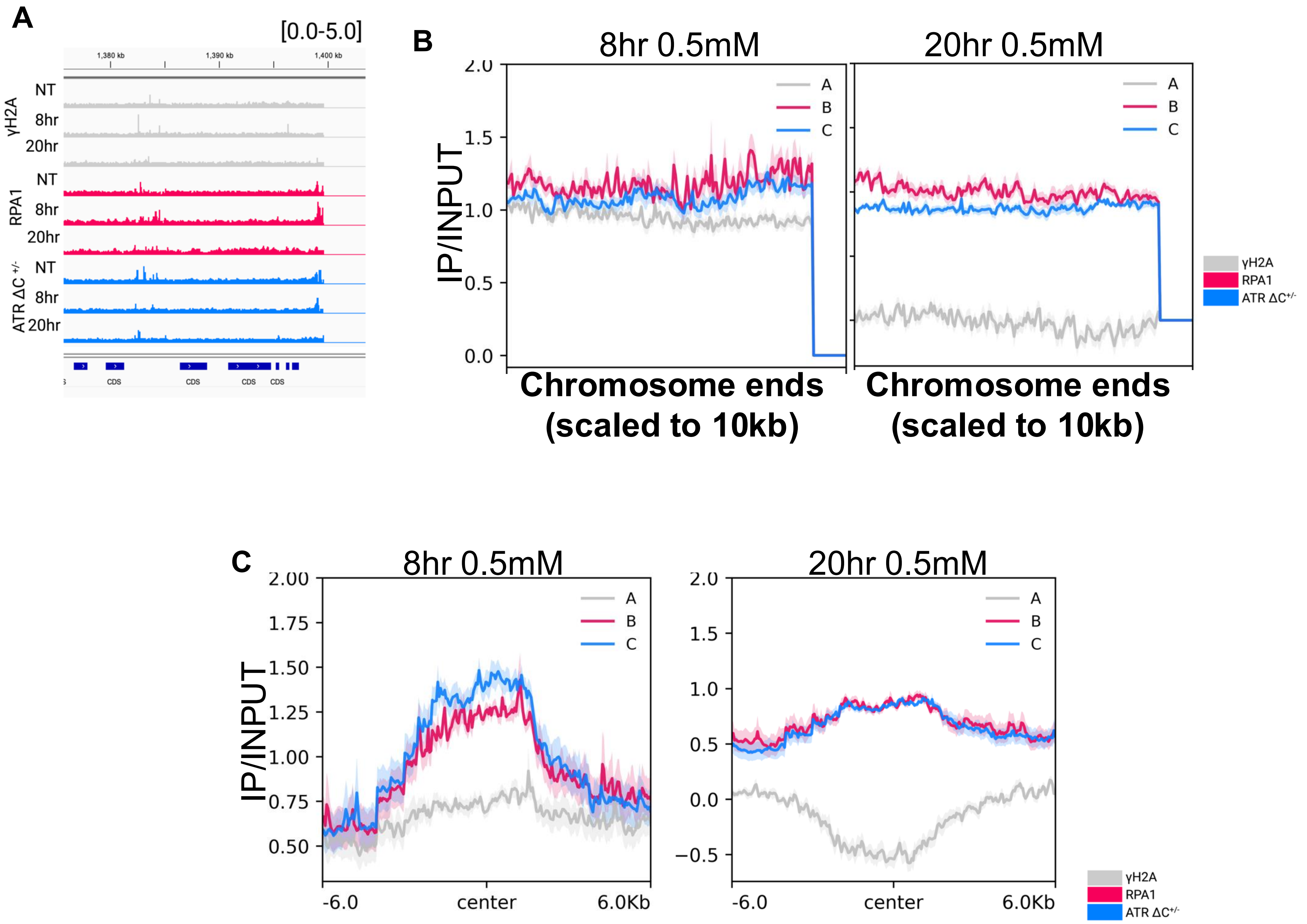
A
[0.0-5.0]
1,380 kb
1,390 kb
1,400 kb
γH2A
NT
8hr
20hr
RPA1
NT
8hr
20hr
ATR ΔC +/-
NT
8hr
20hr
CDS
CDS
CDS
CDS
B
8hr 0.5mM
20hr 0.5mM
IP/INPUT
2.0
1.5
1.0
0.5
0.0
A
B
C
Chromosome ends (scaled to 10kb)
Chromosome ends (scaled to 10kb)
γH2A
RPA1
ATR ΔC+/-
C
8hr 0.5mM
20hr 0.5mM
IP/INPUT
2.00
1.75
1.50
1.25
1.00
0.75
0.50
2.0
1.5
1.0
0.5
0.0
−0.5
-6.0
center
6.0Kb
γH2A
RPA1
ATR ΔC+/-

Figure S8

Figure S8. Subtelomeric accumulation of RS-associated factors in cells treated with 0.5 mM HU for 8 and 20 hrs. (A) Localisation of yH2A and RPA1 in control cells and the location of RPA1 in ATRΔC+/- cells. ChIP-seq signals shown are mapped to a representative subtelomeric region. ChIP-seq samples were normalised to input. Scale = 0.0 - 5.0. Tracks were viewed in IGV. Coding sequences (blue). Merged replicates are shown for RPA1, one replicate is shown for yH2A (B) Metaplot analyses showing the enriched signals for yH2A and RPA1 scaled to chromosome ends (10 kb). ChIP-seq samples were normalised to their corresponding input controls. (C) Metaplot analysis depicting enriched signal for yH2A and RPA1 at 8 hrs and 20 hrs centred on sites enriched for AcH3 within subtelomeric regions enriched for AcH3 (AcH3 Telo) +/- 6 kb. Colours are as per legend.

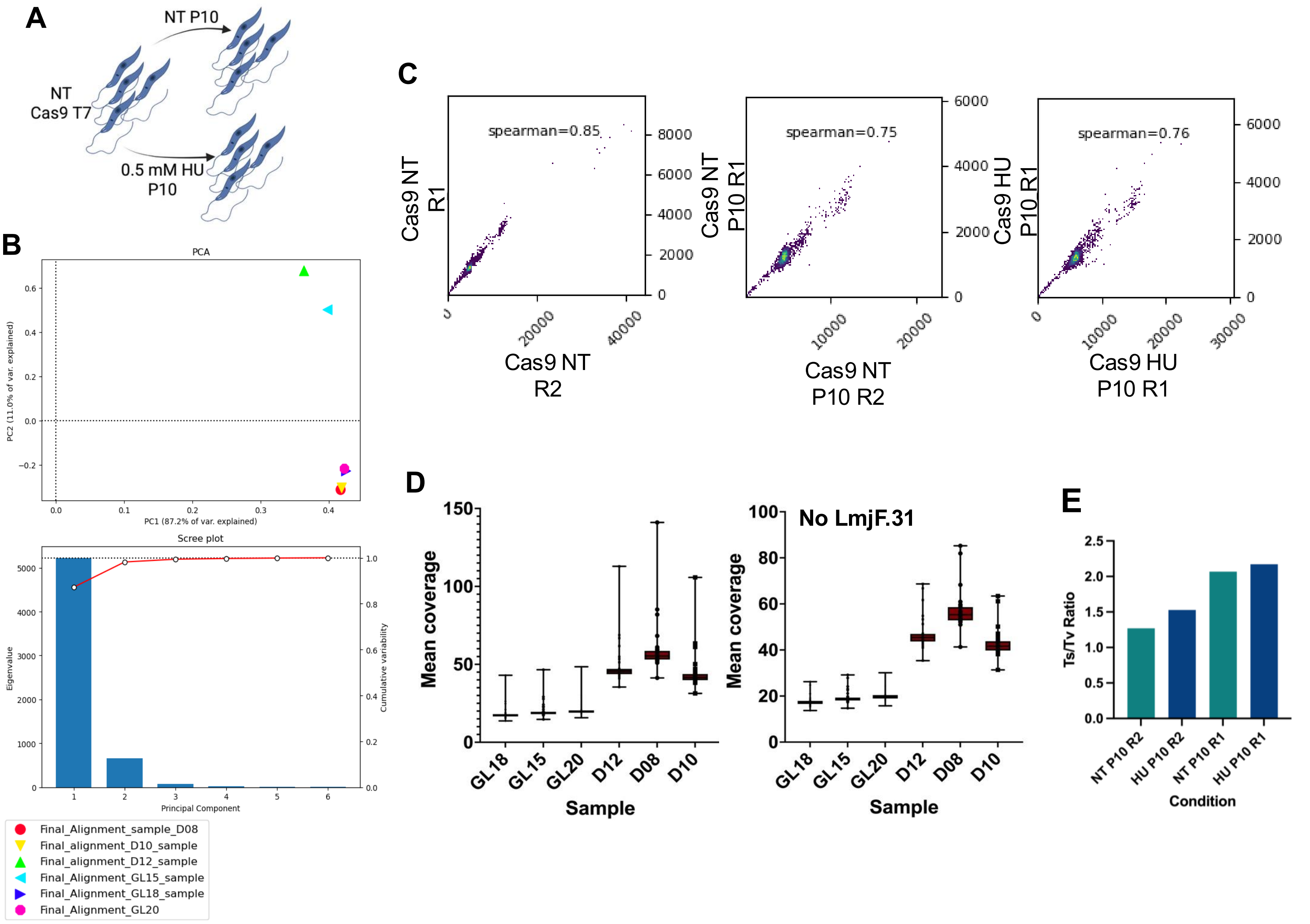
A
NT P10
NT
Cas9 T7
0.5 mM HU
P10
B
PCA
PC2 (11.0% of var. explained)
PC1 (87.2% of var. explained)
Scree plot
Eigenvalue
Cumulative variability
Principal Component
Final_Alignment_sample_D08
Final_alignment_D10_sample
Final_alignment_D12_sample
Final_Alignment_GL15_sample
Final_Alignment_GL18_sample
Final_Alignment_GL20
C
spearman=0.85
Cas9 NT R1
Cas9 NT R2
spearman=0.75
Cas9 NT P10 R1
Cas9 NT P10 R2
spearman=0.76
Cas9 HU P10_R1
Cas9 HU P10 R1
D
Mean coverage
Sample
GL18
GL15
GL20
D12
D08
D10
No LmjF.31
E
Ts/Tv Ratio
NT P10 R2
HU P10 R2
NT P10 R1
HU P10 R1
Condition

Figure S9

Figure S9. Whole genome sequencing analysis to test for chromosome copy number changes following RS exposure (A) Schematic illustrating the experimental approach. Untreated Cas9 T7 cells were serially passaged in the presence and absence of exposure to HU for 10 passages *in vitro*. HU exposed cells were grown for 20 hrs in 0.5 mM HU then washed and released into fresh M199. DNA was harvested from untreated cells, cells at passage 10 and cells at passage 10 exposed to HU and subject to next generation sequencing. Samples represent two independent biological replicates. (B) PCA analysis was performed to examine the variance across replicates and conditions. The grouping of samples along PC1 is highly suggestive of variance associated with HU treatment, as confirmed by the Scree plot below. (C) Spearman coefficient correlation analysis to assess the similarity between biological duplicates in untreated, passage 10 and passage 10 following treatment with 0.5 mM HU for 20 hrs. The spearman correlation revealed a strong correlation between replicates. (D) Mean coverage was calculated for each sample across each replicate. Coverage is shown with and without the inclusion of the pentaploid chromosome LmjF. 31. GL = replicate 1, D = replicate 2. (E) Ts/Tv ratios calculated from SNV data.

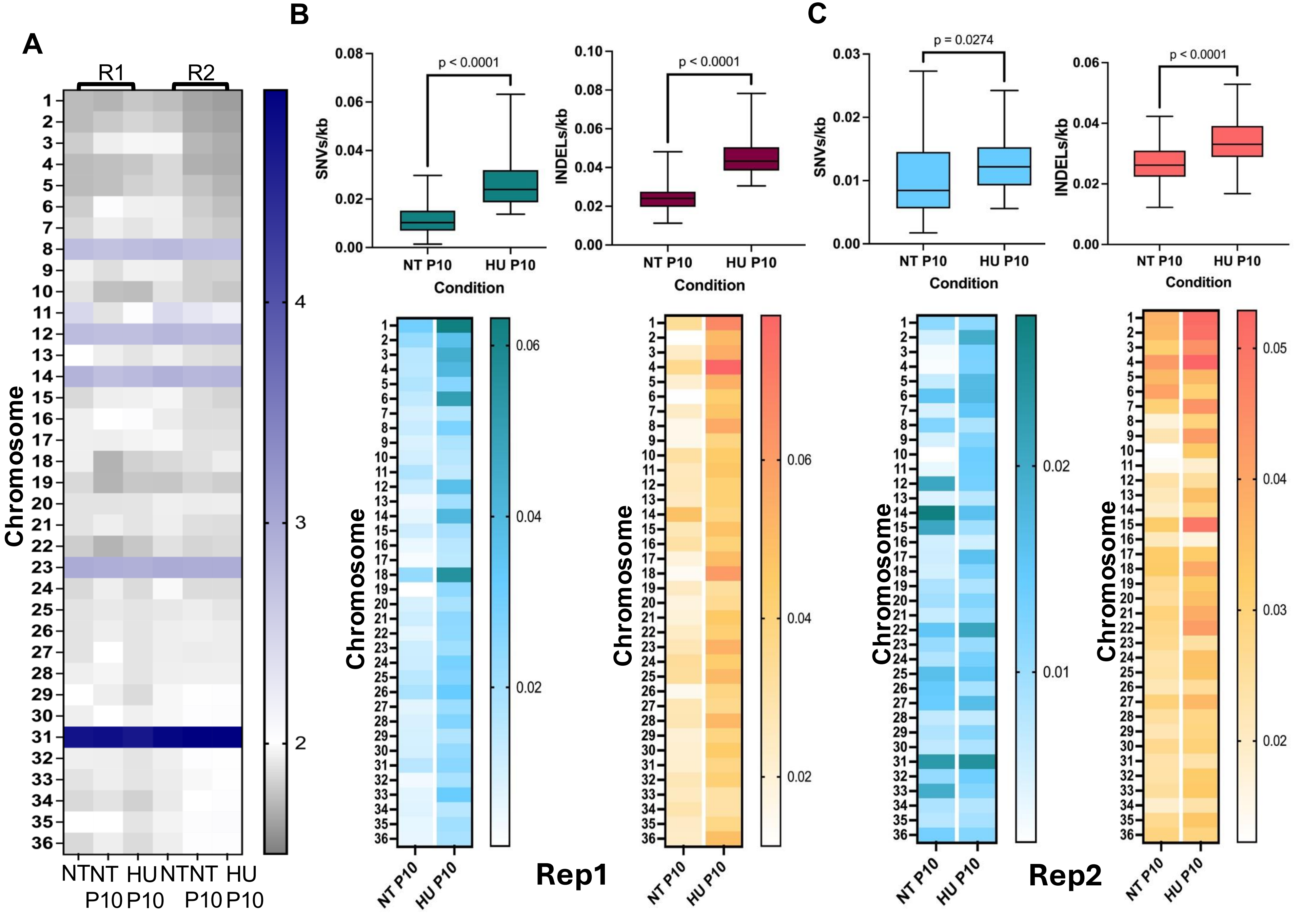

A
B
C
R1
R2
Chromosome
NT P10
HU P10
SNVs/kb
INDELs/kb
Condition
p < 0.0001
p = 0.0274
Rep1
Rep2

Figure S10

Figure S10. Ten passages following exposure to 20 hrs 0.5 mM HU does not significantly alter *Leishmania* chromosome copy number but enhances SNV and INDEL accumulation (A) Heatmap shows the ploidy count for each chromosome (smallest; top, to largest; bottom) of each chromosome in untreated cells (NT), cells passaged for 10 passages and cells passaged for 10 passages after exposure to 0.5 mM HU for 20 hrs. Both replicates are shown. R1 = replicate 1 and R2 = replicate 2. (B) Box and whiskers plot showing the mean SNV count per kb (left) and the mean INDEL count per kb (right) for replicate 1. Significance was examined using a Kruskal-Wallis test with Dunn's multiple comparisons. Below the heatmaps show this data plotted relative to the chromosome number (smallest to largest). (C) Box and whiskers plot showing the mean SNV count per kb (left) and the mean INDEL count per kb (right) for replicate 2. Significance was examined using a Kruskal-Wallis test with Dunn's multiple comparisons. Below the heatmaps show this data plotted relative to the chromosome number (smallest to largest)
